# Varietal Discrimination of Purple, Red, and White Rice Bran Oils Based on Physicochemical Properties, Bioactive Compounds, and Lipidomic Profiles

**DOI:** 10.3390/molecules31020308

**Published:** 2026-01-15

**Authors:** Peng Zheng, Yuyue Qin, Xiaoyu Yin, Jianxin Cao, Shujie Wang, Guiguang Cheng

**Affiliations:** 1 Faculty of Food Science and Engineering, Kunming University of Science and Technology, Kunming 650550, China; zp2440209573@163.com (P.Z.); 13313129@kust.edu.cn (Y.Q.); yinxiaoyu1234@163.com (X.Y.); jxcao321@hotmail.com (J.C.); 2 Yunnan International Joint Laboratory of Green Food Processing, Kunming 650500, China

**Keywords:** rice bran oil, pigmented rice varieties, varietal discrimination, bioactive compounds, lipidomics

## Abstract

Rice bran oil (RBO) is increasingly valued for its bioactive constituents and associated health benefits. This study presents a comprehensive comparative analysis of RBOs derived from purple (PRBO), red (RRBO), and white (WRBO) rice bran, focusing on their physicochemical properties, fatty-acid profiles, bioactive components, antioxidant activity, oxidative stability, and lipidomics. Our results demonstrate that PRBO consistently exhibited a more favorable fatty-acid composition, characterized by a higher proportion of unsaturated fatty acids and significantly greater concentrations of bioactive compounds (including tocopherols/tocotrienols, *γ*-oryzanol, phytosterols, and squalene). Accordingly, PRBO showed the highest radical-scavenging activity and storage oxidative stability, followed by RRBO and WRBO. Additionally, untargeted lipidomics using UPLC–MS–MS identified 2908 lipid species spanning 57 subclasses and revealed distinct variety-specific lipid signatures. PRBO was uniquely enriched in lipid species such as ceramide phosphate (CerP) and monogalactosyldiacylglycerol (MGDG). RRBO was characterized by a distinct abundance of sitosteryl esters (SiE), phosphatidic acid (PA), and cardiolipin (CL), while WRBO was distinguished by phosphatidylethanol (PEt), lysodimethylphosphatidylethanolamine (LdMePE), and sphingomyelin (SM). Overall, PRBO possessed not only a broader repertoire of lipid species but also higher relative abundances of nutritionally significant lipids. These results enable quality evaluation and varietal authentication of colored RBOs and guide their targeted use in health-oriented foods and nutritional interventions.

## 1. Introduction

Rice is one of the most important cereal crops worldwide, ranking among the top in both cultivation area and yield. During rice milling, rice bran accounts for approximately 8–10% of the total grain weight and is rich in lipids, proteins, dietary fiber, and various bioactive compounds, making it a valuable by-product with great potential for high-value utilization. Among these components, rice bran oil (RBO) has been recognized as a “healthy oil” due to its well-balanced fatty acid composition and abundance of functional constituents [1]. RBO contains appropriate proportions of monounsaturated and polyunsaturated fatty acids and is enriched with *γ*-oryzanol, tocopherols, tocotrienols, phytosterols, and squalene [2]. These compounds contribute to lipid regulation, cardiovascular protection, and anti-aging effects, as well as enhancing oxidative stability, thereby broadening the application potential of RBO in edible oils, functional foods, and nutraceuticals [3,4].

In recent years, pigmented rice varieties, including black, purple, and red rice, have gained increasing attention due to their distinctive nutritional profiles and functional properties. Their characteristic colors are primarily attributed to flavonoid compounds such as anthocyanins and proanthocyanidins [5]. Compared with white rice, pigmented rice is remarkably rich in anthocyanins, phenolic acids, flavonoids, *γ*-oryzanol, and proanthocyanidins, which not only confer distinct coloration to the grains but also impart stronger antioxidant activity and multiple health benefits [6]. Previous studies have reported that the anthocyanin content in black rice can be up to 35 times higher than that in red rice, with cyanidin-3-glucoside identified as the predominant component [7]. The antioxidant activity of pigmented rice contributes to anti-inflammatory effects, primarily through the downregulation of the redox-sensitive NF-κB signaling pathway [8]. These bioactive compounds play crucial roles in alleviating oxidative stress, regulating glucose metabolism, and preventing chronic diseases [9].

Research focusing on RBO derived from pigmented rice remains limited. The literature has largely examined the influence of extraction and processing on white RBO quality [10,11], with only a few investigations assessing how different extraction methods modulate bioactive compounds in pigmented versus white RBO [12,13]. However, these investigations largely relied on samples collected from a single region in Thailand. China is a major producer and consumer of rice, and pigmented rice is widely cultivated in regions such as Mojiang (Yunnan, China). Yet, systematic evidence on the physicochemical properties, bioactive profiles, and lipidomic features of pigmented RBOs from China is still scarce, which hampers comprehensive quality evaluation, functional interpretation, and broader utilization. Therefore, the present study selected purple, red, and white rice varieties cultivated in Mojiang, Yunnan Province, China. The physicochemical properties, bioactive components, and oxidative stability of their rice bran oils were systematically analyzed and compared, and lipidomic profiling was conducted to elucidate lipid composition in depth. This study aims to reveal the differences in nutritional and functional attributes among rice bran oils from different varieties, thereby providing a scientific basis for the application of pigmented rice bran oil in functional food development and nutritional interventions, as well as offering new insights into its high-value utilization and varietal discrimination.

## 2. Results and Discussion

### 2.1. Physicochemical Parameters of the RBOs

The physicochemical properties of the three RBO samples were analyzed, and the results are presented in Table 1. On a dry bran basis, the extractable oil yield of the three rice brans ranged from 6.03% to 7.90%. It should be noted that reported oil yields can vary substantially depending on rice variety, bran pretreatment/stabilization, and extraction strategy and conditions. Our yields are comparable to the 9.89% obtained by supercritical CO_2_ extraction (40 °C, 150 bar) reported previously [14]. In contrast, an ultrasound-enhanced supercritical CO_2_ process increased the extraction yield from 9.94 wt% to 12.65 wt% (≈27% improvement) under optimized conditions (160 W, 40 min; 40 °C, 25 MPa), indicating that assisted extraction techniques and operating parameters can markedly affect oil recovery [15].

Iodine value (IV) is commonly used to characterize the degree of unsaturation of oils, and a higher IV generally indicates a higher abundance of C=C double bonds (i.e., a higher unsaturation level) in fatty acids. In this study, the IVs of WRBO and PRBO were 137.46 and 139.00 g I_2_/100 g oil, respectively, with PRBO showing the highest value among the three RBOs. These values are higher than those reported for RBO obtained by ultrasound-assisted cold-press extraction (100.20 g I_2_/100 g oil) [16], and also higher than the ranges reported for mixed-solvent extraction (105.41–106.73 g I_2_/100 g oil) or n-hexane/ethanol solvent extraction (88.51–90.40 g I_2_/100 g oil) [17,18], suggesting that differences in rice variety as well as extraction/processing conditions may markedly influence the unsaturation level of RBO.

Acid value (AV) and peroxide value (POV) are fundamental physicochemical indicators used to evaluate the edibility and oxidative stability of oil. AV (mg KOH/g oil) reflects the extent of hydrolytic rancidity by indicating the amount of free fatty acids released from triacylglycerol hydrolysis, whereas POV (mmol/kg) assesses primary lipid oxidation by quantifying hydroperoxides [14]. As presented in Table 1, the AVs of PRBO, RRBO, and WRBO were 3.68, 9.26, and 9.45 mg KOH/g oil, respectively. The elevated AVs observed in RRBO and WRBO, compared to PRBO, are likely due to higher endogenous lipase activity in the rice bran prior to oil extraction. Lipases catalyze the hydrolysis of triacylglycerols into free fatty acids, especially under conditions of delayed stabilization or improper storage, resulting in increased acid values. The POVs of PRBO, WRBO, and RRBO were 1.04, 1.61, and 1.45 mmol/kg, respectively, all of which are well below the maximum limit of 5.00 mmol/kg established by Codex Stan 210–1999 (Codex Standard for Named Vegetable Oils) [19], indicating that the oxidative quality of all three RBO samples meets acceptable standards.

The color characteristics (L*, a*, and b*) of RBOs derived from different rice varieties are presented in Table 1. These parameters represent lightness (L*), red–green (a*), and yellow–blue (b*) chromatic components, with positive a* indicating redness and positive b* indicating yellowness. The L* values of the samples ranged from 18.98 to 60.12, reflecting marked visual differences among the three RBO types. Notably, PRBO exhibited substantially higher a* and b* values than WRBO and RRBO, which is likely attributable to its higher levels of pigments, such as carotenoids and chlorophylls, that were extracted from purple rice bran by the organic solvent during oil processing. Pigmented RBO is enriched with chlorophyll (32.58 µg/g) and carotenoids (9.82 µg/g), which are considered the main determinants of its coloration [9].

### 2.2. Fatty Acid Composition of RBOs

The fatty acid composition of the different RBOs is summarized in Table 2, comprising nine predominant fatty acids. While the types of fatty acids detected were largely consistent across the RBOs, notable differences were observed in their relative proportions. Oleic acid (C18:1) was the most abundant fatty acid, followed by linoleic acid (C18:2) and palmitic acid (C16:0). This distribution is consistent with the fatty-acid composition of seven RBOs obtained by ultrasound-assisted n-hexane extraction [20]. Nevertheless, the relative abundance of these major fatty acids varies among rice types/varieties in the literature. For example, indica rice bran oil has been reported to show a higher proportion of C18:2 (41.47 ± 0.33%) than C18:1 (36.31 ± 0.16%), with C16:0 at 18.93 ± 0.03% [20]. Such shifts in the C18:1/C18:2 balance and the level of C16:0 further support that the relative percentages of these fatty acids differ significantly among rice varieties (*p* < 0.05). The content of saturated fatty acids (20.51~23.17%) was lower than that of polyunsaturated fatty acids (32.81~35.77%), which in turn was lower than that of monounsaturated fatty acids (43.72~44.11%). The overall ratio of saturated, monounsaturated, and polyunsaturated fatty acids (0.6:1.1:1) approximates the “golden ratio” recommended by the World Health Organization (WHO), highlighting RBO as a nutritionally favorable edible oil. Among the different RBO types, RRBO and WRBO contained significantly higher levels of saturated fatty acids compared with PRBO, whereas PRBO exhibited a significantly greater proportion of polyunsaturated fatty acids. Considering the well-documented role of polyunsaturated fatty acids in lowering serum cholesterol and triglyceride concentrations, PRBO may therefore be regarded as having superior nutritional value.

### 2.3. Bioactive Compounds of RBOs

RBOs have garnered considerable attention owing to their abundance of bioactive constituents, including *γ*-oryzanol, phytosterols, tocopherols, tocotrienols, and squalene. The contents of these compounds in different RBO varieties are summarized in Table 3.

Vitamin E, a well-recognized natural antioxidant in RBO, consists primarily of tocopherols and tocotrienols. In this study, the total vitamin E content was calculated as the sum of *α*-tocopherol, *β*-tocopherol, *γ*-tocopherol, and *α*-tocotrienol. PRBO exhibited the greatest diversity and significantly higher concentrations (*p* < 0.05) of tocopherols and tocotrienols compared with RRBO and WRBO. The elevated levels of these compounds may contribute to the superior oxidative stability of PRBO. Notably, *α*-tocotrienol was only detected in PRBO at 71.99 ± 3.18 mg/kg and was not detected in RRBO or WRBO. Tocotrienols are reported to possess stronger antioxidant activity than tocopherols, as well as additional physiological functions such as cholesterol reduction, hypolipidemic effects, and neuroprotection. In particular, α-tocotrienol demonstrates antioxidant activity in liver microsomes that is 40–60 times higher than that of *α*-tocopherol [21].

*γ*-Oryzanol, another characteristic component of RBO, is a ferulic acid ester mixture that exerts antioxidant activity primarily through its ferulic acid moiety. Beyond its antioxidative role, *γ*-oryzanol has been associated with multiple pharmacological functions, including inhibition of cholesterol accumulation in blood vessels, cardioprotective effects, and the promotion of muscle growth via hormonal stimulation [22]. In the present study, *γ*-oryzanol levels varied across cultivars, with PRBO containing 2.52 ± 0.03 g/100 g, compared with 1.86 ± 0.04 g/100 g and 0.83 ± 0.02 g/100 g in both RRBO and WRBO. This result is consistent with the findings of Pattananandecha, Thanawat et al., who reported that the *γ*-oryzanol content of rice bran oil obtained by cold pressing was 3.37 g/100 g [23].

Squalene, a triterpenoid compound serving as a key precursor in plant sterol biosynthesis, was also detected at substantially higher levels in PRBO (500.17 mg/kg) than in RRBO (211.25 mg/kg) and WRBO (90.59 mg/kg). Owing to its multiple isoprene double bonds, squalene exhibits a wide range of biological activities, including immunomodulatory, antitumor, and circulation-promoting effects [24].

Phytosterols, another important class of bioactive lipids in RBO, are structurally similar to cholesterol and are known for their cholesterol-lowering, antioxidant, and antitumor properties [25]. Among these, *β*-sitosterol was identified as the predominant sterol. PRBO and RRBO exhibited significantly higher phytosterol contents than WRBO, consistent with previous reports.

Taken together, PRBO demonstrated a greater diversity and higher concentrations of bioactive compounds, particularly tocotrienols, *γ*-oryzanol, squalene, and phytosterols, compared with RRBO and WRBO. These findings suggest that PRBO possesses enhanced nutritional and health-promoting potential, underscoring its value as a functional edible oil.

### 2.4. Radical-Scavenging Capacity of RBOs

**Figure 1:**
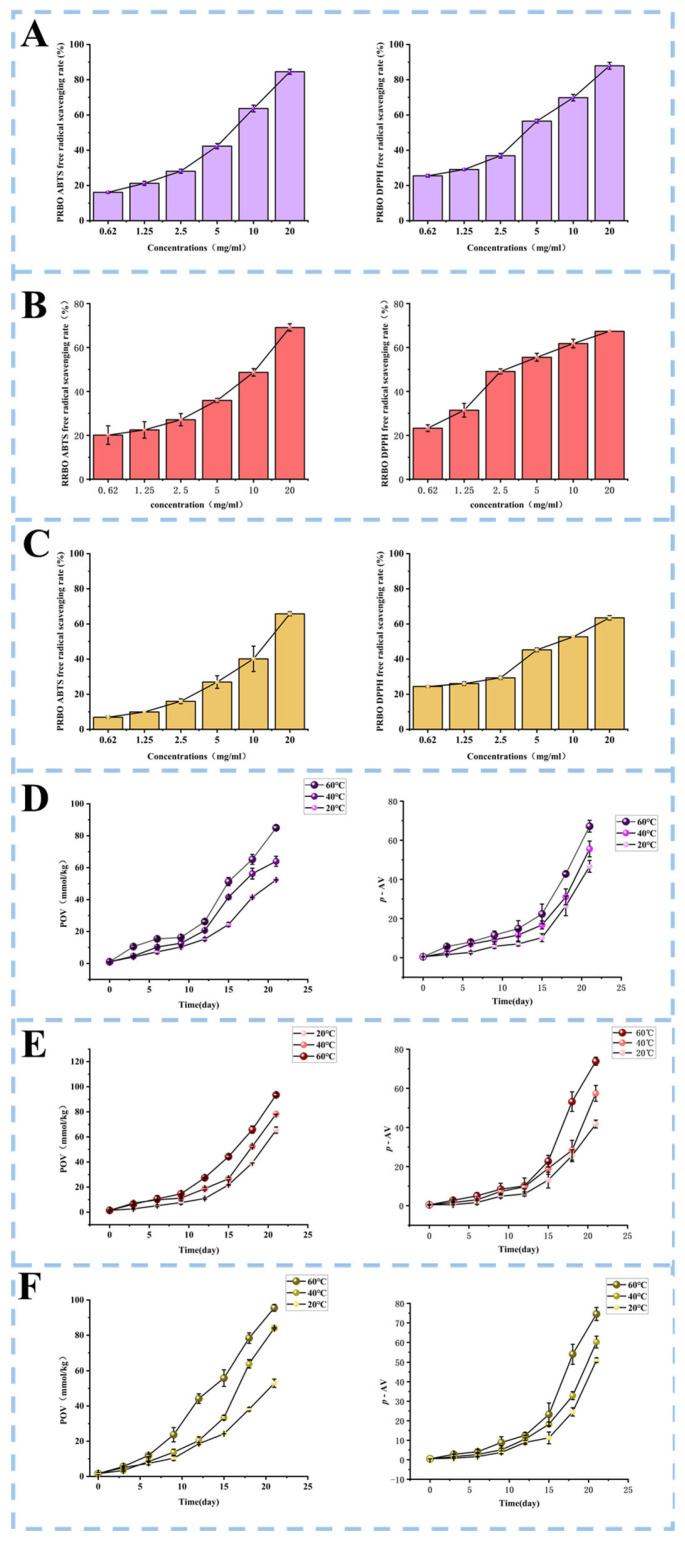
Antioxidant activities and oxidative stability of three types of rice bran oil (WRBO, RRBO, and PRBO). (**A**–**C**) ABTS and DPPH radical scavenging activities; (**D**–**F**) changes in POV and *p*-AV at different storage temperatures.

The radical-scavenging capacity antiradical scavenging activity of crude RBOs was assessed using in vitro assays, and the results are shown in Figure 1. Both DPPH and ABTS radical scavenging activities increased in a concentration-dependent manner across all three RBOs. Antioxidant capacity was further quantified by calculating IC_50_ values, defined as the concentration required to scavenge 50% of free radicals. For DPPH radical scavenging, the IC_50_ values were 3.50 mg/mL for PRBO, 4.12 mg/mL for RRBO, and 7.73 mg/mL for WRBO. Similarly, the IC_50_ values for ABTS radical scavenging were 5.55 mg/mL, 9.31 mg/mL, and 12.46 mg/mL for PRBO, RRBO, and WRBO, respectively. Since lower IC_50_ values indicate stronger antioxidant activity, these results clearly demonstrate that PRBO possesses the highest radical scavenging capacity among the three oils. The superior antioxidant activity of PRBO is likely attributable to its elevated levels of bioactive compounds, including tocopherols, phytosterols, squalene, and *γ*-oryzanol, which are known to effectively terminate free radical chain reactions [26].

### 2.5. Oxidation Stability of RBOs

Oxidative stability was determined based on POV and *p*-anisidine value (*p*-AV) to reflect primary and secondary oxidation, respectively. As illustrated in Figure 1, both POV and *p*-AV increased progressively in all oil samples during 21 d of storage at different temperatures, reflecting continuous lipid oxidation. After 21 d at 60 °C, the POV reached 85.00 mmol/kg in PRBO, 95.56 mmol/kg in WRBO, and 93.52 mmol/kg in RRBO, while the corresponding *p*-AV values were 74.57 ± 3.35, 73.85 ± 2.05, and 67.17 ± 3.02, respectively. Elevated temperatures accelerate oxidative deterioration by enhancing free radical formation and promoting the generation as well as decomposition of hydroperoxides, thereby intensifying lipid oxidation [27]. Notably, PRBO showed the lowest increases in both POV and *p*-AV, indicating superior oxidative stability compared with WRBO and RRBO. This finding is consistent with the higher radical-scavenging capacity previously observed in PRBO.

### 2.6. Lipidomics Profiling of RBOs

Lipidomics analysis was conducted to systematically characterize and compare the lipid molecular species among the three RBO samples. As summarized in Table S1, a total of 2908 identified lipids were detected under both positive and negative ionization modes, including 2358 in positive mode and 550 in negative mode, reflecting the remarkable molecular diversity of RBO.

**Figure 2:**
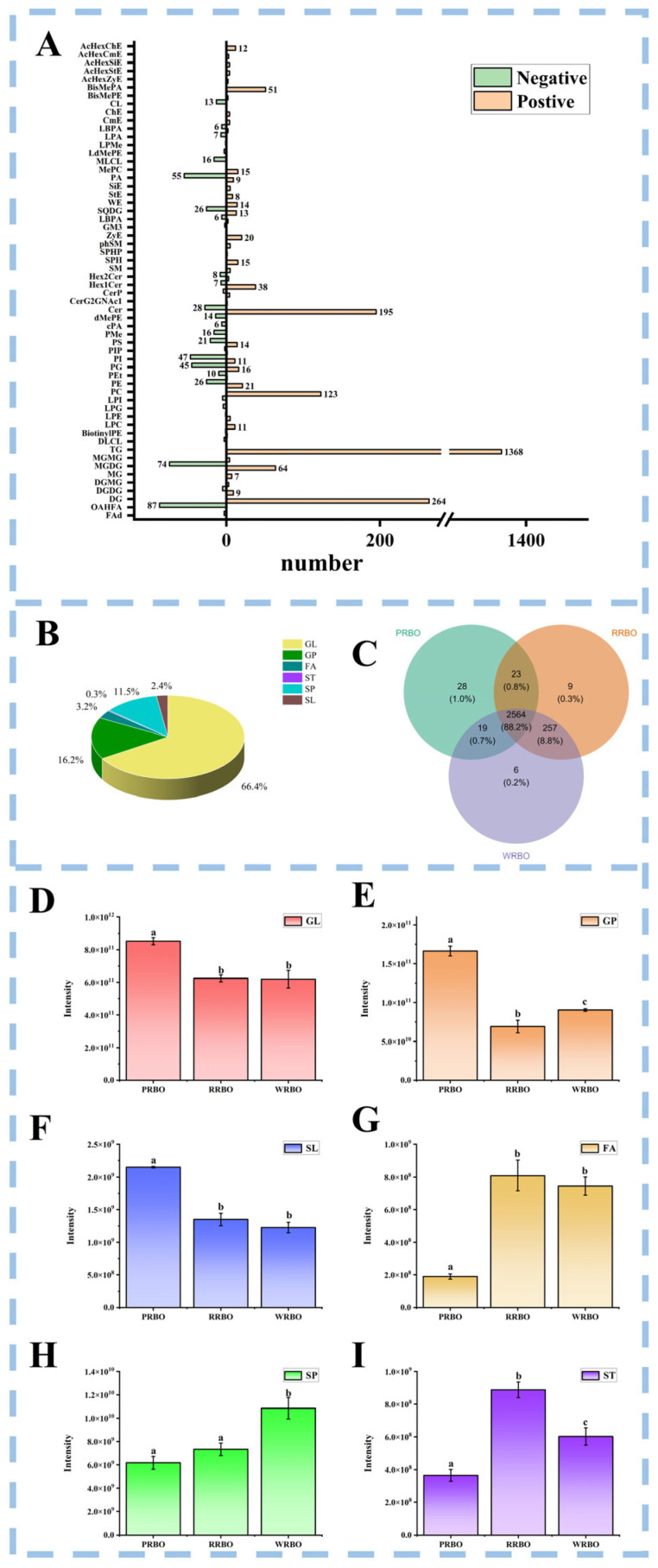
Lipid profiles of RBOs and comparative analysis of major lipid categories among different RBO types. (**A**) Number of lipid subclasses identified in RBOs. (**B**) Pie chart showing the distribution of major lipid categories in RBOs. (**C**) Venn diagram illustrating shared and unique lipid species among the three RBO types. (**D**–**I**) Abundance of major lipid categories, including GL, GP, SP, SL, FA, and ST. Values labeled with different lowercase letters indicate significant differences at *p* < 0.05.

In the positive ion mode (Figure 2A), 2358 lipids were classified into 6 categories covering 44 subclasses. Triacylglycerols (TG) constituted the dominant class (n = 1368), followed by diacylglycerols (DG, n = 264), ceramides (Cer, n = 195), and phosphatidylcholines (PC, n = 123). Major ion adducts included [M + NH_4_]^+^, [M + Na]^+^ and [M + H]^+^, consistent with the preferential ionization of neutral and zwitterionic lipids. By contrast, negative ion mode facilitated the detection of 550 lipids distributed across 6 categories and 31 subclasses. Among them, O-acyl-1-hydroxy fatty acids (OAHFA, n = 87), monogalactosyldiacylglycerols (MGDG, n = 74), phosphatidic acids (PA, n = 55), phosphatidylinositols (PI, n = 47), and phosphatidylglycerols (PG, n = 45) were particularly prominent. The predominant ion type was [M − H]^−^, reflecting the enhanced ionization of acidic and polar lipids.

Altogether, the identified lipids were grouped into 6 major categories—glycerolipids (GL), glycerophospholipids (GP), fatty acyls (FA), sphingolipids (SP), saccharolipids (SL), and sterol lipids (ST)—spanning 57 subclasses (Figure 2B). Glycerolipids (GL) were the dominant lipid class, comprising 66.40% of the total lipids, with TG (46.90%) as the major constituents. GP accounted for 16.20% of total lipids and was the most structurally diverse, including 26 subclasses such as cardiolipin (CL), PC, PA, phosphatidylethanolamine (PE), PG, PI, lysophosphatidylcholine (LPC), lysophosphatidylethanolamine (LPE), lysophosphatidylglycerol (LPG), lysophosphatidic acid (LPA), and phosphatidylserine (PS). SP contributed 11.50% of the total and encompassed 10 subclasses, including Cer, ceramide phosphate (CerP), monosialodihexosylganglioside (GM3), dihydroxyceramides (Hex2Cer), monohexosylceramides (Hex1Cer), plant sphingosine (phSM), sphingomyelins (SM), sphingosine-1-phosphate (SPHP), ceramide glycosides (CerG2GNAc1), and sphingosines (SPH). FA accounted for 3.20%, including fatty acids (FAd), OAHFA, and wax esters (WE). SL represented 2.40%, consisting of 5 subclasses such as digalactosyldiacylglycerols (DGDG), MGDG, monogalactosyl monoacylglycerols (MGMG), digalactosyl monoacylglycerols (DGMG), and sulfoquinovosyldiacylglycerols (SQDG). STs were the least abundant (0.30%), but included nine subclasses such as cholesteryl ester (ChE), campesteryl ester (CmE), sitosteryl ester (SiE), acylhexosyl campesteryl ester (AcHexCmE), acylhexosyl cholesteryl ester (AcHexChE), acylhexosyl sitosteryl ester (AcHexSiE), acylhexosyl zymosterol (AcHexZyE), acylhexosyl sterol ester (AcHexStE), sterol ester (StE), and zymosterol (ZyE). Overall, RBOs were dominated by GL, particularly TG, but also displayed remarkable diversity across GP and SP, providing a biochemical basis for their nutritional and functional properties.

To further explore variety-dependent differences, lipid classes were quantified by summing the peak areas of individual lipids within each class (Figure 2D–I). GL exhibited the highest abundance (>7 × 10^11^), with PRBO containing the greatest proportion, while no significant difference was observed between RRBO and WRBO (*p* > 0.05). GL was mainly composed of TG, DG, and MG. TGs serve as the primary energy storage molecules in plant oils, though certain TG species are also known to exert bioactivities beneficial to human health [28]. GP levels were significantly higher in PRBO than in WRBO and RRBO, which may suggest a greater presence of amphiphilic phospholipids with potential interfacial activity (i.e., natural emulsifier potential) in food systems [29]. SP showed marked variation, with PRBO reaching levels of ~5.7 × 10^9^, substantially lower than WRBO and RRBO. Due to their amphiphilic structure, SPs can self-assemble into micelles/vesicles; therefore, differences in SP abundance among RBOs may imply different potential contributions to interfacial properties in relevant systems [30]. STs were significantly more abundant in RRBO compared with PRBO and WRBO, and their physiological relevance is underscored by evidence linking insufficient ST intake to metabolic and cardiovascular disorders such as hypercholesterolemia, cerebrovascular disease, and hypertension [31]. FA levels also varied, with WRBO showing the highest abundance and PRBO the lowest. These observations collectively indicate that rice variety exerts a significant impact on lipid composition, shaping both nutritional value and potential functional applications of RBO. These findings indicated that PRBO contains more lipids overall and is particularly enriched in functional glycerophospholipids, conferring advantages in nutritional quality and potential applications.

**Figure 3:**
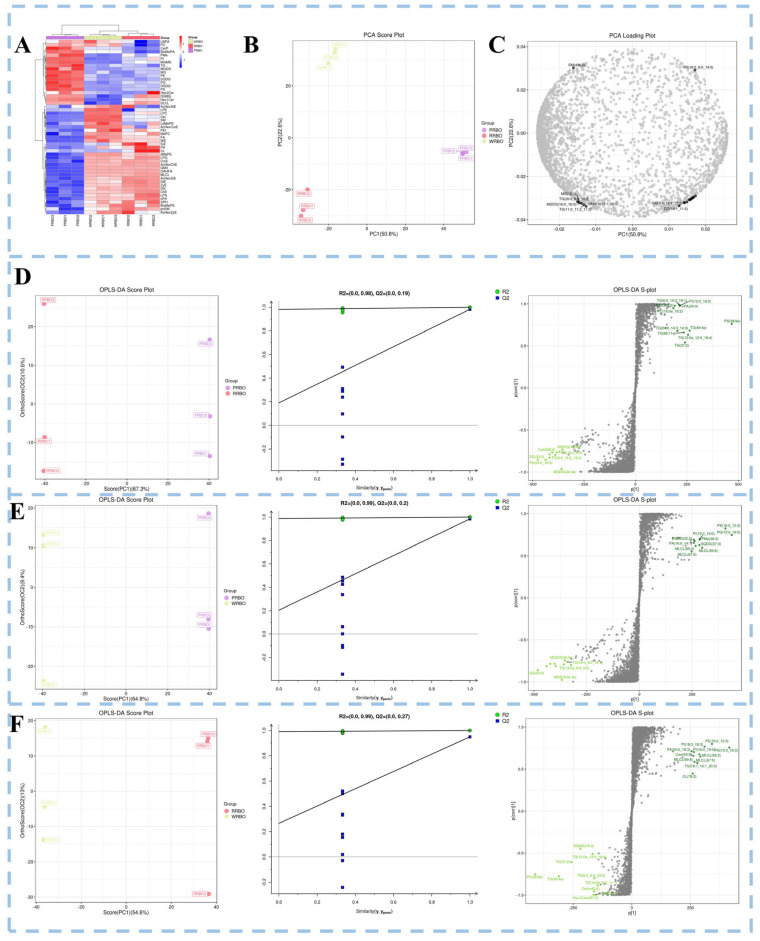
The differences in lipid subtypes and molecules in different RBOs. (**A**) Heat map of differential lipid clustering of different RBOs, with red representing high abundance and blue representing low abundance. (**B**) Score graphs of PCA of three types of RBO. (**C**) Load diagrams for PCA of three types of RBO, pairwise comparative analysis of RBOs based on OPLS-DA models. (**D**–**F**) Score plots, permutation test plots, and S-plots of the OPLS-DA models for PRBO vs. RRBO, PRBO vs. WRBO, and RRBO vs. WRBO, respectively. In the S-plots, green dots indicate lipid species with VIP values > 1, whereas gray dots represent lipid species with VIP values ≤ 1.

Analysis of lipid subclasses revealed further variety-specific distinctions (Figure 3A). PRBO was enriched in CerP and MGDG. CerP is a bioactive sphingolipid metabolite involved in inflammatory regulation, cell survival, and tissue repair [32], while MGDG has been implicated in immune modulation and inflammatory signaling, with reported anticancer and antiviral properties [33]. In RRBO, SiE, PA, and cardiolipin (CL) were predominant. SiE is essential in sterol homeostasis and lipid metabolism, PA acts as a key mediator of membrane biogenesis and intracellular signaling, and CL is critical for mitochondrial structure and energy metabolism [34,35]. By contrast, WRBO was characterized by enrichment of phosphatidylethanol (PEt), lysodimethylphosphatidylethanolamine (LdMePE), and SM. SM is notable for its roles in brain and neural development, gut microbiota regulation, skin barrier enhancement, and lipid metabolism [36].

Multivariate statistical analysis further supported the distinct lipidomic profiles. PCA models (Figure 3B) showed tight clustering of RBO, confirming analytical stability, and clear separation of the three RBO groups, with PC1 and PC2 explaining 50.80% and 22.60% of the variance, respectively. Loading plots (Figure 3C) revealed that SM (d32:2) contributed strongly to WRBO, while OAHFA (18:1/12:0) and DG (18:1/11:2) were more characteristic of PRBO. Characteristic lipids of RRBO included MG (18:1), TG (20:0/6:0/12:2), MGDG (16:0/16:0), OAHFA (18:1/26:0) and TG (11:0/11:2/11:2). Complementary OPLS-DA analyses (Figure 3) confirmed clear separation among RBO groups and robustness of the models, as validated by 200-permutation tests. Based on VIP > 1 and *p* < 0.05, 1747, 1640, and 1257 differential lipids were identified in PRBO vs. RRBO, PRBO vs. WRBO, and WRBO vs. RRBO, respectively. For the PRBO–RRBO comparison, the differential lipidome was dominated by TG (n = 815), DG (n = 179), and Cer (n = 157), chiefly within GL, GP, and SL. PRBO was distinguished by TG (6:0/10:2/18:1), PI (12:0/18:2), MLCL (69:7), cPA (24:0), PC (18:0e/18:2), PS (38:6e), TG (24:0/14:3/14:3), TG (49:4e), TG (66:11e), TG (12:0e/12:0/18:4), and TG (37:2); by contrast, RRBO was characterized by MGDG (36:3e), Cer (d35:2), PA (16:0/8:0), DG (33:0), TG (9:0/18:2/18:2), PA (24:0/18:3), and MGDG (34:3e) (Figure 3D). PRBO compared with WRBO, the differential set comprised TG (n = 733), Cer (n = 168), and DG (n = 140). Discriminant species enriched in PRBO included DGDG (32:2), PA (16:0/14:1), MLCL (65:2; 67:5; 69:8), PI (16:0/19:0), PMe (38:3), SQDG (37:3), PE (16:0/12:3), PG (15:0/16:0), DG (33:0), MGDG (36:3e; 34:3e), and TG (14:0e/8:0/9:0; 16:0/9:0/16:1) (Figure 3E). For RRBO vs. WRBO, the differential lipidome was dominated by TG (n = 524), Cer (n = 156), and DG (n = 118). Distinctive markers for WRBO comprised PE (16:0/12:3), PI (18:3/18:3), PA (24:0/18:3), PI (16:0/19:0), PG (15:0/16:0), Cer (t43:5), MLCL (65:2; 67:5; 69:8), TG (16:1/16:1/20:3), CL (78:2), DGMG (18:2), TG (12:0e/12:0/18:4), TG (37:2), PS (38:6e), TG (49:4e), TG (6:0/6:0/23:0), TG (18:0e/6:0/11:1), Cer (m42:6), LPA (15:0), and Hex1Cer (d37:2) (Figure 3F). Most differential lipids were concentrated in GL and GP subclasses, consistent with previous reports that subclass-specific TG, DG, and GP species serve as discriminant markers in plant oils such as mango kernel oil [37]. In summary, although the lipidomes of RBOs are dominated by glycerolipids, they exhibit pronounced variety-specific differences. Distinct patterns of subclass enrichment and discriminant markers were identified for PRBO, RRBO, and WRBO. These variety-specific lipidomic fingerprints not only delineate their unique nutritional and functional characteristics but also provide potential biomarkers for the quality-based discrimination of RBOs.

## 3. Materials and Methods

### 3.1. Materials

Red rice bran and white rice bran were provided by Yuanyang County (Yunnan Province, China), while purple rice bran was sourced from Mojiang Hani Autonomous County (Yunnan Province, China). Phytosterol standards (*β*-sitosterol, stigmasterol and cholestanol), eight tocopherol homologues (*α*-tocopherol, *β*-tocopherol, *γ*-tocopherol, *δ*-tocopherol, *α*-tocotrienol, *β*-tocotrienol, *γ*-tocotrienol and *δ*-tocotrienol), squalene, squalane, 2,2′-azino-bi (3-ethylbenzothiazoline-6-sulfonic acid) (ABTS), and 2,2-diphenyl-1-picrylhydrazyl (DPPH) were purchased from Sigma-Aldrich Co. (Shanghai, China). Chromatographic-grade methanol, n-heptane, isopropanol, n-hexane, acetonitrile, and ethanol were obtained from Merck KGaA (Darmstadt, Germany). Ultrapure water was prepared using a Milli-Q water purification system (Millipore Corporation, Bedford, MA, USA). All other chemicals were of analytical grade.

### 3.2. Sample Preparation

The rice bran stabilization was conducted as previously described [38], with minor modifications. Rice bran samples were evenly spread in a domestic microwave oven (WD800G, 800 W, Galanz Co., Foshan, China) and subjected to microwave heating for 2.5 min per cycle. This treatment was repeated three times, with 5 min intervals between cycles to allow for natural cooling. The microwave-treated bran was subsequently mixed with methyl tert-butyl ether (MTBE) at a solid-to-liquid ratio of 1:7 (*w*/*v*). Extraction was carried out using ultrasonic treatment at 40 kHz for 30 min in an ultrasonic bath (SB-4200DTD, Ningbo Scientz Biotechnology Co., Ningbo, China), followed by mechanical agitation with a digital overhead stirrer at 800 rpm for 2 h. The resulting mixture was centrifuged at 4000 rpm for 15 min in a benchtop centrifuge (CLT55, Hunan Xiangyi Laboratory Instrument Development Co., Ltd., Changsha, China). The supernatant was concentrated by rotary evaporation at 35 °C to yield PRBO, RRBO, and WRBO. All oils were stored at −20 °C until further analysis.

### 3.3. Physicochemical Analysis

The AV of RBO was determined according to the American Oil Chemists’ Society (AOCS) Official Method Cd 3d-63 [39], using ethanol–potassium hydroxide titration. The IV was measured following the AOCS Official Method Cd 1-87 [40], based on the Wijs method. The POV was determined in accordance with the AOCS Official Method Cd 8-53 [41], using sodium thiosulfate titration. The color was determined by CR-400/410 chromaticity meter (Konica Minolta, Japan), and the results are reported in CIELAB parameters (L*, a*, b*).

### 3.4. Fatty Acid Composition

Fatty acid methyl esters (FAMEs) were prepared by BF_3_-methanol derivatization according to the AOCS official method Ce 2-66 [42]. The analyses were performed using a gas chromatography–flame ionization detector (GC-FID) system (7890B, Agilent, Santa Clara, CA, USA) equipped with a DB-FastFAME column (30 m × 0.25 mm × 0.25 μm). The oven temperature program was set as follows: the initial temperature was 80 °C with a 0.5 min hold, increased to 165 °C at 40 °C/min and held for 1 min, followed by a ramp to 230 °C at 4 °C/min with a final hold of 4 min. Nitrogen was employed as the carrier gas at a constant flow rate of 1.0 mL/min. Samples were injected in split mode (20:1) at 250 °C. The detector temperature was maintained at 300 °C, with hydrogen and air flow rates of 40 mL/min and 450 mL/min, respectively. FAMEs were identified by comparing retention times with those of a certified FAME standard mixture. Quantification was based on peak-area normalization, and results were expressed as the relative percentage of each fatty acid in total fatty acids.

### 3.5. Bioactive Compound Analysis

#### 3.5.1. Oryzanol

According to the method described by Wang et al. [18], a 0.2 g sample was dissolved in heptane and transferred into a 10 mL quartz cuvette. The absorbance of the resulting solution was measured at 315 nm using a UV–Vis spectrophotometer (Beijing Pusa General Instrument Co., Ltd., Beijing, China).

#### 3.5.2. Phytosterols

Phytosterols were quantified according to a published method with minor modifications [20]. Samples (0.1 g) were mixed with 2 mL of 1 mg/mL internal standard solution (cholestanol in n-hexane) and 10 mL of ethanolic KOH (2 M), followed by saponification at 80 °C for 1.5 h in an oil bath shaker (350 rpm). The reaction was subsequently quenched with 10 mL of deionized water and extracted three times with n-hexane (3 × 5 mL). The combined organic phases were concentrated using rotary evaporation, derivatized with 200 μL of BSTFA (containing 1% TMCS) and 200 μL of pyridine at 60 °C for 1 h, and reconstituted in n-hexane. The resulting solution was dehydrated over anhydrous Na_2_SO_4_, filtered through a 0.22 μm PTFE membrane, and subjected to GC analysis.

GC analysis was performed on a 7890B gas chromatograph (Agilent, Santa Clara, CA, USA) equipped with a CP-TAP CB capillary column (25 m × 250 μm × 0.1 μm) and a flame ionization detector (FID) maintained at 361 °C. The injector temperature was set at 280 °C with a split ratio of 1:10. The oven temperature program was as follows: initial temperature at 100 °C (held for 1 min), ramped at 15 °C/min to 310 °C (held for 2 min), followed by an increase at 1.5 °C/min to a final temperature of 315 °C. Hydrogen and air were supplied to the detector at flow rates of 30 mL/min and 300 mL/min, respectively. A 1 μL sample was injected in constant flow mode using helium as the carrier gas. Sterol peaks were identified by comparing their relative retention times with those of authentic standards. Quantification was performed using cholestanol (1 mg/mL) as the internal standard, and phytosterol contents were reported as mg/kg oil.

#### 3.5.3. Tocopherols and Tocotrienols

The tocopherol and tocotrienols content were determined according to AOCS Official Method Ce 8-89 [43] using HPLC (Alliance 2695 HPLC system, Waters, Milford, MA, USA) equipped with a SunFire™ Prep Silica column (4.6 × 250 mm, 5 μm). The mobile phase consisted of n-hexane and isopropanol (99.2:0.8, *v*/*v*) and was delivered isostatically at a flow rate of 0.8 mL/min. The column temperature was maintained at 30 °C. Fluorescence detection was performed using a Waters 2475 detector, with excitation and emission wavelengths set at 290 nm and 330 nm, respectively. The injection volume was 10 μL. Tocopherols and tocotrienols were identified against reference standards and quantified via standard calibration curves, with concentrations expressed as mg kg^−1^ oil.

#### 3.5.4. Squalene

The squalene content was analyzed according to a literature procedure [44]. Samples (2.0 g) were mixed with 1 mg/mL squalane internal standard solution in round-bottom flasks. Saponification was carried out with 20 mL of ethanolic KOH (2 M) under reflux at 80 °C for 50 min. After cooling, the mixtures were sequentially extracted with n-hexane (3 × 15 mL), washed with ethanol (2 × 10 mL), concentrated using a rotary evaporator (40 °C, 200 mbar), and reconstituted to 10 mL with n-hexane.

Analysis was performed on a GC-2010 Pro gas chromatograph (Shimadzu, Kyoto, Japan) equipped with a DB-1ht capillary column (30 m × 0.25 mm × 0.1 μm) and a flame ionization detector (FID) maintained at 380 °C. The injector temperature was set at 350 °C with a split ratio of 10:1. The oven temperature program was as follows: initial temperature of 50 °C for 1 min, increased to 180 °C at 25 °C/min, ramped to 230 °C at 7 °C/min, and finally raised to 360 °C at 45 °C/min, where it was held for 14 min. Hydrogen fuel gas and compressed air were supplied to the detector at flow rates of 30 mL/min and 300 mL/min, respectively, while helium was used as the carrier gas at 1.5 mL/min. Samples (1 μL) were injected automatically using a precision syringe. Squalene was quantified by GC–FID using an internal standard method with squalane (1 mg/mL). Quantification was based on peak-area ratios relative to the internal standard, and the results were expressed as mg squalene per kg oil.

### 3.6. Radical-Scavenging Capacity

#### 3.6.1. DPPH Radical Scavenging Assay

The DPPH radical scavenging activity was determined based on a previously described method with minor modifications [45]. In brief, 0.5 mL of the sample was mixed with 2 mL of DPPH solution prepared in ethanol and incubated in the dark for 30 min. The absorbance was subsequently measured at 517 nm using a microplate reader (SpectraMax M5, Molecular Devices, San Jose, CA, USA). The scavenging activity was expressed as the half-maximal inhibitory concentration (IC_50_), which reflects the efficiency of free radical quenching. The percentage of DPPH scavenging activity was calculated using the following equation:

(1) $$DPPH\;Scavenging\;activity\left(\%\right)=\left(1-\frac{A_{2}-A_{1}}{A_{0}}\right)\times 100$$

where *A*_0_, *A*_1_ and *A*_2_ represent the absorbance of DPPH-ethanol solution, samples without DPPH-ethanol solution, and samples with DPPH-ethanol solution, respectively.

#### 3.6.2. ABTS Radical Scavenging Assay

The ABTS radical-scavenging activity was determined as previously described [46], with slight modifications. The ABTS stock solution was diluted to an absorbance of 0.70 ± 0.02 at 734 nm. Subsequently, 0.5 mL of sample solution (20, 10, 5, 2.5, 1.25, 0.625 mg/mL) was mixed with 4.0 mL of the ABTS working solution, followed by incubation in the dark at 25 °C for 6 min. The absorbance of the reaction mixture was then recorded at 734 nm. The radical scavenging activity was calculated using the following equation:

(2) $$ABTS\;Scavenging\;activity\left(\%\right)=\left(1-\frac{A_{2}-A_{1}}{A_{0}}\right)\times 100$$

where *A*_0_ represents the absorbance of the ABTS solution without sample, *A*_2_ is the absorbance after addition of the sample, and *A*_1_ is the absorbance of the blank (sample with ethanol instead of ABTS). The half-maximal inhibitory concentration (IC_50_) values were obtained from the dose–response curves.

### 3.7. Oxidative Stability Evaluation

The oxidative stability of RBO was assessed through the Schaal oven method. Approximately 10 g of each oil sample was transferred into a brown ground-neck bottle and stored in a forced-draft oven maintained at 20, 40, and 60 °C for a total of 21 d. Samples were withdrawn at predetermined time points (0, 3, 6, 9, 12, 15, 18, and 21 d) to analyze the POV, and *p*-anisidine value (*p*-AV). POV were determined every three days using the titration method described in Section 3.3. The *p*-AV were determined using the Cd 18–90 official methods.

### 3.8. Lipidomics Analysis

Lipidomic profiling was performed based on a published protocol with minor modifications [37]. Lipids were extracted twice using a chloroform/methanol mixture (2:1, *v*/*v*) through sequential vortexing (30 s), incubation on ice (40 and 10 min), and centrifugation at 12,000 rpm for 5 min at 25 °C. The organic phases (300 μL and 400 μL, respectively) from each extraction were collected and combined. The pooled extracts were concentrated under vacuum, reconstituted in 200 μL of isopropanol, and filtered through 0.22 μm nylon membranes. Lipidomic analysis was performed using UPLC-MS-MS equipped with an ACQUITY UPLC^®^ BEH C18 column (2.1 × 100 mm, 1.7 µm, Waters Milford, MA, USA), employing acetonitrile/isopropanol as the mobile phase supplemented with 10 mM ammonium formate and 0.1% formic acid. LC separation was carried out on a Vanquish UHPLC System (Thermo Fisher Scientific, Waltham, MA, USA), and mass spectrometric detection was conducted on a Q Exactive mass spectrometer (Thermo Fisher Scientific, Waltham, MA, USA).

The raw data files were imported into LipidSearch (v4.2.28) for lipid annotation on a sample-by-sample basis. Peak alignment and filtering were performed in LipidSearch with bR.T.Tolerance = 0.25 and m-Score Threshold = 3. Identification was based on MS/MS spectral matching/scoring implemented in LipidSearch, and quantification was obtained from the aligned peak response (peak intensity/peak area) values. To enable comparison across samples, the intensities were corrected by sum peak normalization.

### 3.9. Statistical Analysis

All experiments were conducted in triplicate, and results were expressed as mean ± standard deviation (SD). Statistical differences among groups were analyzed using one-way analysis of variance (ANOVA), followed by Duncan’s multiple range test at a significance level of *p* < 0.05, performed in SPSS version 22.0 (SPSS Inc., Chicago, IL, USA). Data visualization, including linear regression fitting, was carried out using Origin 2021 (OriginLab, One Roundhouse Plaza, Northampton, MA, USA). Multivariate statistical analyses, including principal component analysis (PCA), partial least squares discriminant analysis (PLS-DA), and orthogonal PLS-DA (OPLS-DA), were conducted using the ropls package (version 1.36.0) in R (version 4.3.1).

## 4. Conclusions

This study systematically characterized the physicochemical properties, fatty acid composition, bioactive compounds, antioxidant activities, oxidative stability, and lipidomic profiles of purple, red, and white rice bran oils. Significant compositional and functional differences were observed among the three varieties, with purple RBO exhibiting higher levels of unsaturated fatty acids and bioactive compounds, accompanied by superior antioxidant capacity and oxidative stability. Lipidomic identified 2908 lipid species spanning 57 subclasses across six major categories—glycerolipids (GL), glycerophospholipids (GP), fatty acyls (FA), sphingolipids (SP), saccharolipids (SL), and sterol lipids (ST)—revealing clear variety specificity. PRBO possessed a richer repertoire of lipid species with higher relative abundances and was enriched in ceramide phosphate (CerP) and monogalactosyldiacylglycerol (MGDG); RRBO was characterized by sitosteryl esters (SiE), phosphatidic acid (PA), and cardiolipin (CL); WRBO was enriched in phosphatidylethanol (PEt), lysodimethylphosphatidylethanolamine (LdMePE), and sphingomyelin (SM). Pairwise comparisons yielded 1747, 1640, and 1257 differential lipids for PRBO vs. RRBO, PRBO vs. WRBO, and WRBO vs. RRBO, respectively, corroborating systematic divergence at the molecular level. Notably, this study presents the first comprehensive lipidomic comparison of pigmented RBOs, delivering novel insights into their compositional diversity and potential health benefits. Collectively, the findings provide a solid theoretical basis for the quality evaluation and functional characterization of RBOs, while offering practical guidance for their utilization in health-oriented foods and other functional applications.

## Figures and Tables

**Table 1:** Physicochemical characteristics of three types of RBO.

	PRBO	RRBO	WRBO
Extraction ratio (%)	7.90 ± 0.30 ^a^	6.90 ± 0.46 ^b^	6.03 ± 0.29 ^c^
Acid value (mg KOH/g oil)	3.68 ± 0.04 ^a^	9.26 ± 0.01 ^b^	9.45 ± 0.06 ^c^
Iodine value (g I_2_/100 g oil)	139.00 ± 2.40 ^a^	127.35 ± 4.50 ^b^	124.46 ± 2.47 ^b^
Peroxide value (POV) (mmol/kg)	1.04 ± 0.17 ^a^	1.45 ± 0.54 ^b^	1.61 ± 0.62 ^c^
L*	18.98 ± 0.09 ^a^	53.16 ± 0.02 ^b^	60.12 ± 0.01 ^c^
a*	9.35 ± 0.15 ^a^	1.31 ± 0.03 ^b^	1.43 ± 0.06 ^c^
b*	36.35 ± 0.34 ^a^	38.81 ± 0.04 ^b^	14.98 ± 0.06 ^c^

L*, luminosity; a*, green to red; b*, blue to yellow. All data are presented as mean ± standard deviation (*n* = 3). Values within the same row followed by different letters (a, b, c) indicate significant differences at *p* < 0.05.

**Table 2:** Fatty acid profiles (% of total fatty acids) of various RBO types.

Sample	PRBO	RRBO	WRBO
C16:0	15.47 ± 0.03 ^a^	16.78 ± 0.03 ^b^	16.79 ± 0.03 ^b^
C18:0	1.77 ± 0.02 ^a^	2.43 ± 0.01 ^b^	2.43 ± 0.01 ^b^
C18:1	43.12 ± 0.39 ^a^	43.59 ± 0.14 ^a^	43.51 ± 0.15 ^a^
C18:2	34.17 ± 0.18 ^a^	31.75 ± 0.08 ^b^	31.59 ± 0.30 ^b^
C18:3	1.61 ± 0.01 ^a^	1.20 ± 0.02 ^b^	1.23 ± 0.06 ^b^
C20:0	0.86 ± 0.02 ^a^	0.93 ± 0.02 ^b^	0.92 ± 0.01 ^b^
C20:1	0.60 ± 0.02 ^a^	0.53 ± 0.03 ^b^	0.51 ± 0.03 ^b^
C22:0	0.56 ± 0.02 ^a^	0.37 ± 0.01 ^b^	0.38 ± 0.01 ^b^
C24:0	1.86 ± 0.03 ^a^	2.42 ± 0.09 ^b^	2.65 ± 0.14 ^b^
SFA	20.51 ± 0.05 ^a^	22.93 ± 0.02 ^b^	23.17 ± 0.03 ^b^
MUFS	43.72 ± 0.38 ^a^	44.11 ± 0.17 ^a^	44.02 ± 0.17 ^a^
PUFA	35.77 ± 0.18 ^a^	32.95 ± 0.08 ^b^	32.81 ± 0.24 ^b^

All data are presented as mean ± standard deviation (*n* = 3). Values within the same row followed by different letters (a, b) indicate significant differences at *p* < 0.05.

**Table 3:** Comparative analysis of bioactive compound contents among the three types of RBO.

Sample	PRBO	RRBO	WRBO
*α*-tocopherol (mg/kg)	220.82 ± 5.94	ND	ND
*β*-tocopherol (mg/kg)	290.32 ± 0.94 ^a^	42.18 ± 1.59 ^b^	10.19 ± 0.28 ^c^
*γ*-tocopherol (mg/kg)	691.57 ± 5.59 ^a^	105.2 ± 2.34 ^b^	ND
*α*-tocotrienol (mg/kg)	71.99 ± 3.18	ND	ND
Squalene (mg/kg)	500.17 ± 7.62 ^a^	211.25 ± 0.94 ^b^	90.59 ± 1.15 ^c^
Phytosterol (mg/g)	0.20 ± 0.02 ^a^	0.20 ± 0.06 ^a^	0.14 ± 0.01 ^b^
*γ*-oryzanol (g/100 g)	2.52 ± 0.03 ^a^	1.86 ± 0.04 ^b^	0.83 ± 0.02 ^c^

All data are presented as mean ± standard deviation (*n* = 3). ND: not detected. Values within the same row followed by different letters (a, b, c) indicate significant differences at *p* < 0.05.

## Data Availability

The data that support the findings of this study are available from the corresponding author upon reasonable request.

## Supplementary Materials

The following supporting information can be downloaded at: https://www.mdpi.com/article/10.3390/molecules31020308/s1, Table S1: Lipid molecules identified in three RBOs.

## Abbreviations

The following abbreviations are used in this manuscript:

AV	Acid value
ABTS	2,2′-azino-bi (3-ethylbenzothiazoline-6-sulfonic acid)
AcHexChE	Acylhexosyl cholesteryl ester
AcHexCmE	Acylhexosyl campesteryl ester
AcHexSiE	Acylhexosyl sitosteryl ester
AcHexStE	Acylhexosyl sterol ester
AcHexZyE	Acylhexosyl zymosterol
ANOVA	Analysis of variance
AOCS	American Oil Chemists’ Society
Cer	Ceramides
CerG2GNAc1	Ceramide glycosides
CerP	Ceramide phosphate
ChE	Cholesteryl ester
CL	Cardiolipin
CmE	Campesteryl ester
DG	Diacylglycerols
DGDG	Digalactosyldiacylglycerols
DGMG	Digalactosyl monoacylglycerols
DPPH	2,2-diphenyl-1-picrylhydrazyl
FA	Fatty acyls
FAd	Fatty acids
FAMEs	Fatty acid methyl esters
FID	Flame ionization detector
GC-FID	Gas chromatography–flame ionization detector
GL	Glycerolipids
GM3	Monosialodihexosylganglioside
GP	Glycerophospholipids
Hex1Cer	Monohexosylceramides
Hex2Cer	Dihydroxyceramides
HPLC	High Performance Liquid Chromatography
IV	Iodine value
LC-MS	Liquid chromatography–mass spectrometry
LdMePE	Lysodimethylphosphatidylethanolamine
LPA	Lysophosphatidic acid
LPC	Lysophosphatidylcholine
LPE	Lysophosphatidylethanolamine
LPG	Lysophosphatidylglycerol
MGDG	Monogalactosyldiacylglycerols
MGMG	Monogalactosyl monoacylglycerols
MTBE	Methyl tert-butyl ether
OAHFA	O-acyl-1-hydroxy fatty acids
OPLS-DA	Orthogonal PLS-DA
PA	Phosphatidic acid
*p*-AV	*P*-anisidine value
PC	Phosphatidylcholines
PCA	Principal component analysis
PE	Phosphatidylethanolamine
PEt	Phosphatidylethanol
PG	Phosphatidylglycerols
phSM	Plant sphingosine
PI	Phosphatidylinositols
POV	Peroxide value
PRBO	Purple rice bran oil
PS	Phosphatidylserine
RBO	Rice bran oil
RRBO	Red rice bran oil
SD	Standard deviation
SiE	Sitosteryl esters
SL	Saccharolipids
SM	sphingomyelin
SP	Sphingolipids
SPH	Sphingosines
SPHP	Sphingosine-1-phosphate
SQDG	Sulfoquinovosyldiacylglycerols
ST	Sterol lipids
StE	Sterol ester
TG	Triacylglycerols
WE	Wax esters
WHO	World Health Organization
WRBO	White rice bran oil
ZyE	Zymosterol
