# Supplementary material for: Varietal Discrimination of Purple, Red, and White Rice Bran Oils Based on Physicochemical Properties, Bioactive Compounds, and Lipidomic Profiles
Source: Molecules. 2026 Jan 15;31(2):308. doi: 10.3390/molecules31020308 (PMC12843884; doi:10.3390/molecules31020308)
Supplement: Supplementary file 1 [file molecules-31-00308-s001.zip › molecules-4030869-supplementary.pdf]

Table S1. Lipid molecules identified in three RBOs

Lipid molecules	Class	Adduct	Formula	RT (min)	MS (*m/z*)	Type
AcHexChE(16:0)	AcHexChE	M+NH4	C49 H90 O7 N1	17.168	804.67118 05	POS
AcHexChE(18:1)	AcHexChE	M+H	C51 H89 O7	15.86	813.66028 15	POS
AcHexChE(18:2)	AcHexChE	M+H	C51 H87 O7	15.065267 03	811.64463 15	POS
AcHexChE(18:3)	AcHexChE	M+H	C51 H85 O7	14.146	809.62898 15	POS
AcHexChE(22:0)	AcHexChE	M+NH4	C55 H102 O7 N1	19.154210 79	888.76508 05	POS
AcHexChE(24:0)	AcHexChE	M+NH4	C57 H106 O7 N1	21.101	916.79638 05	POS
AcHexChE(24:1)	AcHexChE	M+NH4	C57 H104 O7 N1	19.084965 38	914.78073 05	POS
AcHexChE(24:3)	AcHexChE	M+H	C57 H97 O7	17.671725 84	893.72288 15	POS
AcHexChE(24:4)	AcHexChE	M+H	C57 H95 O7	16.931935 06	891.70723 15	POS
AcHexChE(32:0)	Ac • HexC hE	M+NH4	C65 H122 O7 N1	21.383	1028.9215 8	POS
AcHexChE(37:4)	AcHexChE	M+H	C70 H121 O7	19.795	1073.9106 81	POS
AcHexChE(6:0)	AcHexChE	M+H	C39 H67 O7	7.921	647.48813 15	POS
AcHexCmE(13:1)	AcHexCmE	M+NH4	C47 H84 O7 N1	15.32	774.62423 05	POS
AcHexCmE(16:0)	AcHexCmE	M+NH4	C50 H92 O7 N1	19.038849 85	818.68683 05	POS
AcHexCmE(33:2)	AcHexCmE	M+H	C67 H119 O7	19.683	1035.8950 31	POS
AcHexSiE(16:0)	AcHexSiE	M+NH4	C51 H94 O7 N1	19.217696 99	832.70248 05	POS
AcHexSiE(18:1)	AcHexSiE	M+NH4	C53 H96 O7 N1	17.934	858.71813 05	POS
AcHexSiE(18:2)	AcHexSiE	M+NH4	C53 H94 O7 N1	17.051	856.70248 05	POS
AcHexSiE(20:0)	AcHexSiE	M+NH4	C55 H102 O7 N1	20.324	888.76508 05	POS
AcHexStE(22:3)	AcHexStE	M+H	C57 H95 O7	15.486	891.70723 15	POS

AcHexStE(28:0)	AcHexStE	M+NH4	C63 H116 O7 N1	20.636	998.8746305	POS
AcHexStE(30:3)	AcHexStE	M+NH4	C65 H114 O7 N1	19.498	1020.85898	POS
AcHexStE(38:1)	AcHexStE	M+H	C73 H131 O7	22.002	1119.988931	POS
AcHexZyE(24:0)	AcHexZyE	M+H	C57 H101 O7	19.04570055	897.7541815	POS
AcHexZyE(32:0)	AcHexZyE	M+H	C65 H117 O7	20.027	1009.879381	POS
BiotinylPE(33:3)	BiotinylPE	M-H	C48 H83 O10 N3 S1 P1	3.039	924.5542305	NEG
BiotinylPE(39:0)	BiotinylPE	M+H	C54 H103 O10 N3 S1 P1	10.173	1016.709633	POS
BisMePA(16:0/22:3)	BisMePA	M+NH4	C43 H83 O8 N1 P1	13.789	772.5850835	POS
BisMePA(16:1/18:1)	BisMePA	M+NH4	C39 H77 O8 N1 P1	16.108	718.5381335	POS
BisMePA(16:1/18:2)	BisMePA	M+NH4	C39 H75 O8 N1 P1	13.80876864	716.5224835	POS
BisMePA(17:1/18:2)	BisMePA	M+NH4	C40 H77 O8 N1 P1	13.79630147	730.5381335	POS
BisMePA(18:1/14:0)	BisMePA	M+Na	C37 H71 O8 N0 P1 Na1	9.272	697.4778795	POS
BisMePA(18:1/18:1)	BisMePA	M+NH4	C41 H81 O8 N1 P1	14.823	746.5694335	POS
BisMePA(18:1/18:2)	BisMePA	M+NH4	C41 H79 O8 N1 P1	13.108	744.5537835	POS
BisMePA(18:2/18:2)	BisMePA	M+NH4	C41 H77 O8 N1 P1	13.866	742.5381335	POS
BisMePA(18:3/18:2)	BisMePA	M+NH4	C41 H75 O8 N1 P1	9.653	740.5224835	POS
BisMePA(18:3/18:3)	BisMePA	M+NH4	C41 H73 O8 N1 P1	11.67587646	738.5068335	POS
BisMePA(18:4/18:2)	BisMePA	M+NH4	C41 H73 O8 N1 P1	8.595	738.5068335	POS
BisMePA(18:4/23:0)	BisMePA	M+NH4	C46 H87 O8 N1 P1	11.267	812.6163835	POS
BisMePA(20:0/18:2)	BisMePA	M+NH4	C43 H85 O8 N1 P1	17.019	774.6007335	POS
BisMePA(20:0e/18:1)	BisMePA	M+NH4	C43 H89 O7 N1 P1	12.92	762.6371185	POS

BisMePA(20:1/12:1)	BisMePA	M+Na	C37 H69 O8 N0 P1 Na1	13.035	695.4622295	POS
BisMePA(22:0/18:2)	BisMePA	M+NH4	C45 H89 O8 N1 P1	17.992	802.6320335	POS
BisMePA(22:3/18:2)	BisMePA	M+NH4	C45 H83 O8 N1 P1	13.25969447	796.5850835	POS
BisMePA(22:4/18:2)	BisMePA	M+NH4	C45 H81 O8 N1 P1	12.226	794.5694335	POS
BisMePA(24:2/11:3)	BisMePA	M+NH4	C40 H73 O8 N1 P1	8.312	726.5068335	POS
BisMePA(28:0/18:1)	BisMePA	M+NH4	C51 H103 O8 N1 P1	16.941	888.7415835	POS
BisMePA(28:0/18:2)	BisMePA	M+NH4	C51 H101 O8 N1 P1	17.045	886.7259335	POS
BisMePA(28:1/11:3)	BisMePA	M+NH4	C44 H83 O8 N1 P1	7.555	784.5850835	POS
BisMePA(30:0/18:4)	BisMePA	M+NH4	C53 H101 O8 N1 P1	15.37876446	910.7259335	POS
BisMePA(30:1/18:2)	BisMePA	M+NH4	C53 H103 O8 N1 P1	15.34302632	912.7415835	POS
BisMePA(30:1/18:4)	BisMePA	M+NH4	C53 H99 O8 N1 P1	13.147	908.7102835	POS
BisMePA(31:0/18:4)	BisMePA	M+NH4	C54 H103 O8 N1 P1	13.949	924.7415835	POS
BisMePA(32:0/11:3)	BisMePA	M+NH4	C48 H93 O8 N1 P1	14.49792346	842.6633335	POS
BisMePA(32:0/18:2)	BisMePA	M+NH4	C55 H109 O8 N1 P1	16.35915826	942.7885335	POS
BisMePA(32:0/18:4)	BisMePA	M+Na	C55 H101 O8 N0 P1 Na1	14.486	943.7126295	POS
BisMePA(32:1)	BisMePA	M+NH4	C37 H75 O8 N1 P1	24.551	692.5224835	POS
BisMePA(32:1/18:2)	BisMePA	M+NH4	C55 H107 O8 N1 P1	15.68993785	940.7728835	POS
BisMePA(32:1/18:4)	BisMePA	M+NH4	C55 H103 O8 N1 P1	13.276	936.7415835	POS
BisMePA(33:0/18:2)	BisMePA	M+Na	C56 H107 O8 N0 P1 Na1	15.969	961.7595795	POS
BisMePA(33:1/18:1)	BisMePA	M+NH4	C56 H111 O8 N1 P1	18.082	956.8041835	POS
BisMePA(33:1/18:2)	BisMePA	M+NH4	C56 H109 O8 N1 P1	17.747	954.7885335	POS

BisMePA(34:0/12:4)	BisMePA	M+NH4	C51 H97 O8 N1 P1	16.208	882.6946335	POS
BisMePA(34:0e)	BisMePA	M+NH4	C39 H83 O7 N1 P1	17.044	708.5901685	POS
BisMePA(34:1/10:3)	BisMePA	M+NH4	C49 H93 O8 N1 P1	12.539	854.6633335	POS
BisMePA(34:1/11:4)	BisMePA	M+NH4	C50 H93 O8 N1 P1	13.104	866.6633335	POS
BisMePA(36:0/10:3)	BisMePA	M+NH4	C51 H99 O8 N1 P1	17.139	884.7102835	POS
BisMePA(36:3)	BisMePA	M+NH4	C41 H79 O8 N1 P1	15.201	744.5537835	POS
BisMePA(36:3e)	BisMePA	M+NH4	C41 H81 O7 N1 P1	8.572	730.5745185	POS
BisMePA(36:5)	BisMePA	M+NH4	C41 H75 O8 N1 P1	8.842	740.5224835	POS
BisMePA(38:0/10:4)	BisMePA	M+NH4	C53 H101 O8 N1 P1	12.797	910.7259335	POS
BisMePA(38:0/18:1)	BisMePA	M+NH4	C61 H123 O8 N1 P1	20.70194712	1028.898083	POS
BisMePA(38:0/18:4)	BisMePA	M+NH4	C61 H117 O8 N1 P1	18.352	1022.851133	POS
BisMePA(38:0/6:0)	BisMePA	M+NH4	C49 H101 O8 N1 P1	17.72	862.7259335	POS
BisMePA(47:0)	BisMePA	M+NH4	C52 H107 O8 N1 P1	19.453	904.7728835	POS
BisMePA(48:4)	BisMePA	M+NH4	C53 H101 O8 N1 P1	17.182	910.7259335	POS
BisMePA(50:3)	BisMePA	M+NH4	C55 H107 O8 N1 P1	16.429	940.7728835	POS
BisMePA(52:5)	BisMePA	M+NH4	C57 H107 O8 N1 P1	18.05	964.7728835	POS
BisMePE(16:0/18:1)	BisMePE	M+H	C41 H81 O8 N1 P1	14.8220384	746.5694335	POS
BisMePE(18:1/18:1)	BisMePE	M+H	C43 H83 O8 N1 P1	14.86255824	772.5850835	POS
CL(18:2/18:1/18:2/18:2)	CL	M-H	C81 H143 O17 P2	19.90803641	1449.980605	NEG
CL(62:0)	CL	M-H	C71 H137 O17 P2	20.22663981	1323.933655	NEG
CL(63:0)	CL	M-H	C72 H139 O17 P2	20.41344985	1337.949305	NEG

CL(66:2)	CL	M-H	C75 H141 O17 P2	20.643	1375.964955	NEG
CL(70:1)	CL	M+H	C79 H153 O17 P2	14.948	1436.057757	POS
CL(71:0)	CL	M-H	C80 H155 O17 P2	13.038	1450.074505	NEG
CL(72:6)	CL	M-H	C81 H145 O17 P2	20.24463644	1451.996255	NEG
CL(72:7)	CL	M-H	C81 H143 O17 P2	20.202	1449.980605	NEG
CL(72:8)	CL	M-H	C81 H141 O17 P2	19.5587833	1447.964955	NEG
CL(72:9)	CL	M-H	C81 H139 O17 P2	19.19993766	1445.949305	NEG
CL(74:11)	CL	M-H	C83 H139 O17 P2	19.54904704	1469.949305	NEG
CL(78:2)	CL	M-H	C87 H165 O17 P2	13.972	1544.152755	NEG
CL(80:8)	CL	M-H	C89 H157 O17 P2	13.49	1560.090155	NEG
Cer(d12:0/18:1)	Cer	M+HCOO	C31 H60 O5 N1	11.80818429	526.4476975	POS
Cer(d12:0/20:3)	Cer	M+HCOO	C33 H60 O5 N1	10.401	550.4476975	POS
Cer(d14:0/16:0)	Cer	M+H	C30 H62 O3 N1	12.00353692	484.4724205	POS
Cer(d14:0/18:1)	Cer	M+H	C32 H64 O3 N1	12.17764915	510.4880705	POS
Cer(d14:0/18:2)	Cer	M+H	C32 H62 O3 N1	10.92618912	508.4724205	POS
Cer(d14:1/18:0)	Cer	M+H	C32 H64 O3 N1	13.07059915	510.4880705	POS
Cer(d14:1/18:0;O)	Cer	M+H	C32 H64 O4 N1	10.83209472	526.4829855	POS
Cer(d14:1/18:1)	Cer	M+H	C32 H62 O3 N1	12.00446621	508.4724205	POS
Cer(d14:1/18:1;O)	Cer	M+H	C32 H62 O4 N1	9.617734346	524.4673355	POS
Cer(d14:1/20:0)	Cer	M-H	C34 H66 O3 N1	14.59480848	536.5048175	NEG
Cer(d14:1/22:0)	Cer	M+HCOO	C37 H72 O5 N1	16.10383077	610.5415975	POS

Cer(d15:0/16:1)	Cer	M+H	C31 H62 O3 N1	7.235	496.4724205	POS
Cer(d15:0/17:1)	Cer	M+HCOO	C33 H64 O5 N1	13.33867877	554.4789975	POS
Cer(d15:1/21:2)	Cer	M+HCOO	C37 H68 O5 N1	13.9150919	606.5102975	POS
Cer(d16:0/16:0)	Cer	M+HCOO	C33 H66 O5 N1	13.60209964	556.4946475	POS
Cer(d16:0/18:1)	Cer	M+H	C34 H68 O3 N1	13.73175717	538.5193705	POS
Cer(d16:1/18:0;O)	Cer	M+H	C34 H68 O4 N1	12.37618932	554.5142855	POS
Cer(d16:1/18:1)	Cer	M+H	C34 H66 O3 N1	13.53216998	536.5037205	POS
Cer(d16:1/18:1;O)	Cer	M+H	C34 H66 O4 N1	11.166	552.4986355	POS
Cer(d16:1/20:1)	Cer	M+HCOO	C37 H70 O5 N1	15.29889282	608.5259475	POS
Cer(d16:1/21:2)	Cer	M+HCOO	C38 H70 O5 N1	14.414	620.5259475	POS
Cer(d16:1/22:0;O)	Cer	M+H	C38 H76 O4 N1	17.595	610.5768855	POS
Cer(d16:1/26:0)	Cer	M+HCOO	C43 H84 O5 N1	19.12596177	694.6354975	POS
Cer(d17:1/18:1;O)	Cer	M+H	C35 H68 O4 N1	10.029	566.5142855	POS
Cer(d18:0/16:0)	Cer	M+H	C34 H70 O3 N1	15.09004272	540.5350205	POS
Cer(d18:0/18:0)	Cer	M+HCOO	C37 H74 O5 N1	16.47667421	612.5572475	POS
Cer(d18:0/18:1)	Cer	M+H	C36 H72 O3 N1	15.24630031	566.5506705	POS
Cer(d18:0/20:2)	Cer	M+HCOO	C39 H74 O5 N1	16.42631972	636.5572475	POS
Cer(d18:1/16:0)	Cer	M+H	C34 H68 O3 N1	15.78317473	538.5193705	POS
Cer(d18:1/18:0;2O)	Cer	M+H	C36 H72 O5 N1	13.832	598.5405005	POS
Cer(d18:1/18:0;O)	Cer	M+H	C36 H72 O4 N1	13.91668151	582.5455855	POS
Cer(d18:1/18:1)	Cer	M+HCOO	C37 H70 O5 N1	14.709	608.5259475	POS

Cer(d18:1/18:1;O)	Cer	M+H	C36 H70 O4 N1	12.72819925	580.5299355	POS
Cer(d18:1/20:0)	Cer	M+H	C38 H76 O3 N1	17.45293132	594.5819705	POS
Cer(d18:1/22:0)	Cer	M+H	C40 H80 O3 N1	18.39110741	622.6132705	POS
Cer(d18:1/22:0;O)	Cer	M+H	C40 H80 O4 N1	18.54045281	638.6081855	POS
Cer(d18:1/24:0)	Cer	M+H	C42 H84 O3 N1	19.56996623	650.6445705	POS
Cer(d18:1/25:0)	Cer	M+H	C43 H86 O3 N1	19.45150295	664.6602205	POS
Cer(d18:1/26:0)	Cer	M+H	C44 H88 O3 N1	19.75009493	678.6758705	POS
Cer(d18:2/18:1;O)	Cer	M+H	C36 H68 O4 N1	11.74091077	578.5142855	POS
Cer(d18:2/18:2)	Cer	M+H	C36 H66 O3 N1	12.51612415	560.5037205	POS
Cer(d18:2/20:0;O)	Cer	M-H	C38 H72 O4 N1	15.92576924	606.5466825	NEG
Cer(d18:2/20:1)	Cer	M+H	C38 H72 O3 N1	15.95965952	590.5506705	POS
Cer(d21:1/14:0;O)	Cer	M+H	C35 H70 O4 N1	13.44712089	568.5299355	POS
Cer(d27:2)	Cer	M+HCOO	C28 H52 O5 N1	8.70896788	482.3850975	POS
Cer(d28:0)	Cer	M+HCOO	C29 H58 O5 N1	10.468	500.4320475	POS
Cer(d30:0)	Cer	M+HCOO	C31 H62 O5 N1	12.00353692	528.4633475	POS
Cer(d30:0;O)	Cer	M+HCOO	C31 H62 O6 N1	10.67386202	544.4582625	POS
Cer(d32:1)	Cer	M+HCOO	C33 H64 O5 N1	12.17764915	554.4789975	POS
Cer(d34:0)	Cer	M+H	C34 H70 O3 N1	12.73809463	540.5350205	POS
Cer(d34:1)	Cer	M+HCOO	C35 H68 O5 N1	14.87032095	582.5102975	POS
Cer(d34:2)	Cer	M+H	C34 H66 O3 N1	14.52849627	536.5037205	POS
Cer(d35:0)	Cer	M+HCOO	C36 H72 O5 N1	15.78345296	598.5415975	POS

Cer(d35:1;O)	Cer	M+HCOO	C36 H70 O6 N1	13.44712089	612.5208625	POS
Cer(d35:2)	Cer	M+H	C35 H68 O3 N1	15.165	550.5193705	POS
Cer(d36:0)	Cer	M+CH3COO	C38 H76 O5 N1	16.21	626.5728975	POS
Cer(d36:1)	Cer	M+HCOO	C37 H72 O5 N1	15.24630031	610.5415975	POS
Cer(d36:2;O)	Cer	M+HCOO	C37 H70 O6 N1	14.554	624.5208625	POS
Cer(d36:3)	Cer	M+HCOO	C37 H68 O5 N1	14.20735966	606.5102975	POS
Cer(d36:4)	Cer	M-H	C36 H64 O3 N1	12.86434796	558.4891675	NEG
Cer(d37:3;O)	Cer	M-H	C37 H68 O4 N1	14.5744315	590.5153825	NEG
Cer(d38:0)	Cer	M+HCOO	C39 H78 O5 N1	17.52343795	640.5885475	POS
Cer(d38:0;O)	Cer	M+HCOO	C39 H78 O6 N1	17.29849987	656.5834625	POS
Cer(d38:1)	Cer	M+HCOO	C39 H76 O5 N1	17.45293132	638.5728975	POS
Cer(d38:1;O)	Cer	M+HCOO	C39 H76 O6 N1	16.411	654.5678125	POS
Cer(d38:2)	Cer	M+H	C38 H74 O3 N1	17.13108106	592.5663205	POS
Cer(d38:2;O)	Cer	M+HCOO	C39 H74 O6 N1	15.92576924	652.5521625	POS
Cer(d38:4)	Cer	M+H	C38 H70 O3 N1	14.86868653	588.5350205	POS
Cer(d38:5)	Cer	M+H	C38 H68 O3 N1	10.80428855	586.5193705	POS
Cer(d39:1)	Cer	M+HCOO	C40 H78 O5 N1	17.96856699	652.5885475	POS
Cer(d39:3)	Cer	M+H	C39 H74 O3 N1	16.277	604.5663205	POS
Cer(d40:0)	Cer	M+HCOO	C41 H82 O5 N1	18.47702874	668.6198475	POS
Cer(d40:0;O)	Cer	M+HCOO	C41 H82 O6 N1	18.232	684.6147625	POS
Cer(d40:1)	Cer	M-H	C40 H78 O3 N1	18.39110741	620.5987175	NEG

Cer(d40:1;O)	Cer	M+HCOO	C41 H80 O6 N1	17.560073 39	682.59911 25	POS
Cer(d40:2)	Cer	M+H	C40 H78 O3 N1	18.122760 73	620.59762 05	POS
Cer(d40:3)	Cer	M+H	C40 H76 O3 N1	17.112965 74	618.58197 05	POS
Cer(d40:4)	Cer	M+H	C40 H74 O3 N1	16.119636 57	616.56632 05	POS
Cer(d41:1)	Cer	M+HCOO	C42 H82 O5 N1	18.787461 6	680.61984 75	POS
Cer(d42:1;2O)	Cer	M-H	C42 H82 O5 N1	18.168035 28	680.61984 75	NEG
Cer(d42:2)	Cer	M+HCOO	C43 H82 O5 N1	18.554382 57	692.61984 75	POS
Cer(d43:0;O)	Cer	M+HCOO	C44 H88 O6 N1	19.285694 34	726.66171 25	POS
Cer(d43:1)	Cer	M-H	C43 H84 O3 N1	19.451502 95	662.64566 75	NEG
Cer(d44:0)	Cer	M+HCOO	C45 H90 O5 N1	19.356	724.68244 75	POS
Cer(d44:1)	Cer	M-H	C44 H86 O3 N1	19.750094 93	676.66131 75	NEG
Cer(d44:2)	Cer	M+HCOO	C45 H86 O5 N1	19.354693 32	720.65114 75	POS
Cer(m17:0/21:0)	Cer	M+HCOO	C39 H78 O4 N1	17.157	624.59363 25	POS
Cer(m17:0/22:0)	Cer	M+HCOO	C40 H80 O4 N1	17.686	638.60928 25	POS
Cer(m17:1/16:0)	Cer	M+HCOO	C34 H66 O4 N1	12.838	552.49973 25	POS
Cer(m17:1/18:1;O)	Cer	M+H	C35 H68 O3 N1	7.388	550.51937 05	POS
Cer(m18:0/16:0)	Cer	M+H	C34 H70 O2 N1	15.592268 69	524.54010 55	POS
Cer(m18:0/18:1)	Cer	M+H	C36 H72 O2 N1	15.687279 33	550.55575 55	POS
Cer(m18:0/18:2)	Cer	M+H	C36 H70 O2 N1	14.590395 93	548.54010 55	POS
Cer(m18:0/23:0)	Cer	M+HCOO	C42 H84 O4 N1	18.961	666.64058 25	POS
Cer(m39:0;O)	Cer	M+HCOO	C40 H80 O5 N1	17.407	654.60419 75	POS

Cer(m42:4)	Cer	M+HCOO	C43 H78 O4 N1	18.85	672.5936325	POS
Cer(m42:5)	Cer	M+HCOO	C43 H76 O4 N1	17.90078297	670.5779825	POS
Cer(m42:6)	Cer	M+HCOO	C43 H74 O4 N1	16.953	668.5623325	POS
Cer(m44:5)	Cer	M+HCOO	C45 H80 O4 N1	18.71159178	698.6092825	POS
Cer(m44:6)	Cer	M+HCOO	C45 H78 O4 N1	17.96568364	696.5936325	POS
Cer(m46:5)	Cer	M+HCOO	C47 H84 O4 N1	19.41032836	726.6405825	POS
Cer(m46:6)	Cer	M+HCOO	C47 H82 O4 N1	18.779	724.6249325	POS
Cer(t16:0/14:0)	Cer	M+H	C30 H62 O4 N1	10.601	500.4673355	POS
Cer(t16:0/16:0)	Cer	M+HCOO	C33 H66 O6 N1	12.20555548	572.4895625	POS
Cer(t16:0/18:1)	Cer	M+HCOO	C35 H68 O6 N1	12.41149644	598.5052125	POS
Cer(t16:0/22:2)	Cer	M+HCOO	C39 H74 O6 N1	15.89306896	652.5521625	POS
Cer(t16:0/24:0)	Cer	M+HCOO	C41 H82 O6 N1	18.229	684.6147625	POS
Cer(t16:0/26:2)	Cer	M+HCOO	C43 H82 O6 N1	18.02261656	708.6147625	POS
Cer(t16:1/16:0)	Cer	M+H	C32 H64 O4 N1	12.86622284	526.4829855	POS
Cer(t17:0/16:0)	Cer	M+H	C33 H68 O4 N1	12.995	542.5142855	POS
Cer(t17:0/17:0)	Cer	M+CH3COO	C36 H72 O6 N1	14.52	614.5365125	POS
Cer(t17:0/18:1;2O)	Cer	M+CH3COO	C37 H72 O8 N1	9.707413739	658.5263425	POS
Cer(t17:0/18:2;2O)	Cer	M+CH3COO	C37 H70 O8 N1	8.666	656.5106925	POS
Cer(t17:0/18:3)	Cer	M+CH3COO	C37 H68 O6 N1	11.79794169	622.5052125	POS
Cer(t17:0/19:0)	Cer	M+CH3COO	C38 H76 O6 N1	15.9	642.5678125	POS
Cer(t17:0/20:0)	Cer	M+CH3COO	C39 H78 O6 N1	16.61352084	656.5834625	POS

Cer(t17:0/21:0)	Cer	M+CH3COO	C40 H80 O6 N1	17.168	670.5991125	POS
Cer(t17:0/21:0;O)	Cer	M+CH3COO	C40 H80 O7 N1	16.792	686.5940275	POS
Cer(t17:0/22:0)	Cer	M+CH3COO	C41 H82 O6 N1	17.71950327	684.6147625	POS
Cer(t17:0/24:0)	Cer	M+CH3COO	C43 H86 O6 N1	18.54654782	712.6460625	POS
Cer(t17:0/24:1)	Cer	M+CH3COO	C43 H84 O6 N1	17.596	710.6304125	POS
Cer(t17:0/25:0)	Cer	M+CH3COO	C44 H88 O6 N1	18.89159078	726.6617125	POS
Cer(t17:0/25:1)	Cer	M+HCOO	C43 H84 O6 N1	17.583	710.6304125	POS
Cer(t17:0/26:0)	Cer	M+CH3COO	C45 H90 O6 N1	19.217	740.6773625	POS
Cer(t17:0/26:1)	Cer	M+CH3COO	C45 H88 O6 N1	18.432	738.6617125	POS
Cer(t17:1/18:0)	Cer	M+CH3COO	C37 H72 O6 N1	14.37134571	626.5365125	POS
Cer(t17:1/18:1)	Cer	M+CH3COO	C37 H70 O6 N1	13.01767409	624.5208625	POS
Cer(t17:1/20:0)	Cer	M+CH3COO	C39 H76 O6 N1	15.71864161	654.5678125	POS
Cer(t17:1/24:0;O)	Cer	M+HCOO	C42 H82 O7 N1	17.23304632	712.6096775	POS
Cer(t17:1/25:0;O)	Cer	M+CH3COO	C44 H86 O7 N1	18.20785424	740.6409775	POS
Cer(t17:1/26:0)	Cer	M+CH3COO	C45 H88 O6 N1	18.94986633	738.6617125	POS
Cer(t18:0/15:1)	Cer	M+CH3COO	C35 H68 O6 N1	12.426	598.5052125	POS
Cer(t18:0/16:0)	Cer	M+H	C34 H70 O4 N1	13.76922474	556.5299355	POS
Cer(t18:0/17:0)	Cer	M+HCOO	C36 H72 O6 N1	14.53751303	614.5365125	POS
Cer(t18:0/18:0)	Cer	M+HCOO	C37 H74 O6 N1	15.24619483	628.5521625	POS
Cer(t18:0/18:0;O)	Cer	M+HCOO	C37 H74 O7 N1	14.83696016	644.5470775	POS
Cer(t18:0/18:1)	Cer	M+HCOO	C37 H72 O6 N1	13.95224507	626.5365125	POS

Cer(t18:0/18:2)	Cer	M+HCOO	C37 H70 O6 N1	12.78243694	624.5208625	POS
Cer(t18:0/18:2;O)	Cer	M+CH3COO	C38 H72 O7 N1	15.09300851	654.5314275	POS
Cer(t18:0/18:3)	Cer	M+CH3COO	C38 H70 O6 N1	14.378	636.5208625	POS
Cer(t18:0/18:4)	Cer	M+H	C36 H66 O4 N1	10.463	576.4986355	POS
Cer(t18:0/19:1)	Cer	M+HCOO	C38 H74 O6 N1	14.682	640.5521625	POS
Cer(t18:0/20:0)	Cer	M+H	C38 H78 O4 N1	16.57861723	612.5925355	POS
Cer(t18:0/20:0;O)	Cer	M+H	C38 H78 O5 N1	16.19170754	628.5874505	POS
Cer(t18:0/21:0)	Cer	M+HCOO	C40 H80 O6 N1	17.17375233	670.5991125	POS
Cer(t18:0/21:0;O)	Cer	M+HCOO	C40 H80 O7 N1	16.82442399	686.5940275	POS
Cer(t18:0/22:0)	Cer	M+CH3COO	C42 H84 O6 N1	17.68930218	698.6304125	POS
Cer(t18:0/22:0;O)	Cer	M+H	C40 H82 O5 N1	17.39092594	656.6187505	POS
Cer(t18:0/22:4)	Cer	M+CH3COO	C42 H76 O6 N1	12.09480078	690.5678125	POS
Cer(t18:0/22:5)	Cer	M+HCOO	C41 H72 O6 N1	15.191	674.5365125	POS
Cer(t18:0/23:0)	Cer	M+HCOO	C42 H84 O6 N1	18.15705206	698.6304125	POS
Cer(t18:0/23:0;O)	Cer	M-H	C41 H82 O5 N1	17.8716169	668.6198475	NEG
Cer(t18:0/23:1)	Cer	M+HCOO	C42 H82 O6 N1	18.22789118	696.6147625	POS
Cer(t18:0/23:2)	Cer	M+CH3COO	C43 H82 O6 N1	15.398	708.6147625	POS
Cer(t18:0/24:0)	Cer	M+H	C42 H86 O4 N1	18.54366141	668.6551355	POS
Cer(t18:0/24:0;O)	Cer	M+H	C42 H86 O5 N1	18.30581212	684.6500505	POS
Cer(t18:0/24:2)	Cer	M+H	C42 H82 O4 N1	18.49419579	664.6238355	POS
Cer(t18:0/24:3)	Cer	M+H	C42 H80 O4 N1	17.79974745	662.6081855	POS

Cer(t18:0/24:6)	Cer	M+HCOO	C43 H74 O6 N1	15.281	700.5521625	POS
Cer(t18:0/25:0)	Cer	M+HCOO	C44 H88 O6 N1	18.91893918	726.6617125	POS
Cer(t18:0/25:0;O)	Cer	M-H	C43 H86 O5 N1	18.6897158	696.6511475	NEG
Cer(t18:0/25:3)	Cer	M+CH3COO	C45 H84 O6 N1	15.44	734.6304125	POS
Cer(t18:0/26:0)	Cer	M+HCOO	C45 H90 O6 N1	19.24318831	740.6773625	POS
Cer(t18:0/26:0;O)	Cer	M+HCOO	C45 H90 O7 N1	19.0142135	756.6722775	POS
Cer(t18:0/26:1)	Cer	M-H	C44 H86 O4 N1	19.28747726	692.6562325	NEG
Cer(t18:0/26:6)	Cer	M+H	C44 H78 O4 N1	18.26996418	684.5925355	POS
Cer(t18:1/16:0)	Cer	M+HCOO	C35 H68 O6 N1	12.83052613	598.5052125	POS
Cer(t18:1/18:0)	Cer	M+HCOO	C37 H72 O6 N1	14.36650898	626.5365125	POS
Cer(t18:1/18:1)	Cer	M+HCOO	C37 H70 O6 N1	14.54484117	624.5208625	POS
Cer(t18:1/18:1;O)	Cer	M+H	C36 H70 O5 N1	7.380769117	596.5248505	POS
Cer(t18:1/18:2)	Cer	M+HCOO	C37 H68 O6 N1	11.79905536	622.5052125	POS
Cer(t18:1/20:0;O)	Cer	M+H	C38 H76 O5 N1	15.35830653	626.5718005	POS
Cer(t18:1/22:0;O)	Cer	M+H	C40 H80 O5 N1	15.93216987	654.6031005	POS
Cer(t18:1/23:1)	Cer	M+HCOO	C42 H80 O6 N1	17.59068365	694.5991125	POS
Cer(t18:1/24:0)	Cer	M+HCOO	C43 H84 O6 N1	18.59982041	710.6304125	POS
Cer(t18:1/24:0;O)	Cer	M+H	C42 H84 O5 N1	17.13522947	682.6344005	POS
Cer(t18:1/25:0;O)	Cer	M-H	C43 H84 O5 N1	18.20230685	694.6354975	NEG
Cer(t20:1/18:2)	Cer	M+H	C38 H72 O4 N1	12.84815713	606.5455855	POS
Cer(t20:1/18:3)	Cer	M+H	C38 H70 O4 N1	11.223	604.5299355	POS

Cer(t20:1/18:4)	Cer	M+H	C38 H68 O4 N1	11.058	602.5142855	POS
Cer(t28:1)	Cer	M+H	C28 H56 O4 N1	9.717701987	470.4203855	POS
Cer(t31:0)	Cer	M+CH3COO	C33 H66 O6 N1	12.23419676	572.4895625	POS
Cer(t34:1;2O)	Cer	M-H	C34 H66 O6 N1	13.894	584.4895625	NEG
Cer(t34:1;O)	Cer	M+CH3COO	C36 H70 O7 N1	15.06488576	628.5157775	POS
Cer(t36:1;2O)	Cer	M-H	C36 H70 O6 N1	15.34032877	612.5208625	NEG
Cer(t36:1;O)	Cer	M+CH3COO	C38 H74 O7 N1	15.2674595	656.5470775	POS
Cer(t36:2;2O)	Cer	M+HCOO	C37 H70 O8 N1	8.72	656.5106925	POS
Cer(t36:3;O)	Cer	M+CH3COO	C38 H70 O7 N1	14.128	652.5157775	POS
Cer(t36:4)	Cer	M+CH3COO	C38 H68 O6 N1	14.14073759	634.5052125	POS
Cer(t38:0;O)	Cer	M-H	C38 H76 O5 N1	16.19170754	626.5728975	NEG
Cer(t38:1)	Cer	M-H	C38 H74 O4 N1	16.70349912	608.5623325	NEG
Cer(t38:1;O)	Cer	M-H	C38 H74 O5 N1	15.35830653	624.5572475	NEG
Cer(t38:2;2O)	Cer	M-H	C38 H72 O6 N1	15.425	638.5365125	NEG
Cer(t38:2;O)	Cer	M+HCOO	C39 H74 O7 N1	13.99397035	668.5470775	POS
Cer(t38:3;O)	Cer	M-H	C38 H70 O5 N1	12.69835599	620.5259475	NEG
Cer(t39:0;O)	Cer	M-H	C39 H78 O5 N1	16.82442399	640.5885475	NEG
Cer(t39:1)	Cer	M+HCOO	C40 H78 O6 N1	17.429	668.5834625	POS
Cer(t39:1;O)	Cer	M-H	C39 H76 O5 N1	16.03609798	638.5728975	NEG
Cer(t39:2)	Cer	M+HCOO	C40 H76 O6 N1	16.539	666.5678125	POS
Cer(t39:3)	Cer	M+HCOO	C40 H74 O6 N1	15.55497324	664.5521625	POS

Cer(t39:5)	Cer	M+HCOO	C40 H70 O6 N1	13.29380156	660.5208625	POS
Cer(t40:0;O)	Cer	M-H	C40 H80 O5 N1	17.39092594	654.6041975	NEG
Cer(t40:1)	Cer	M+HCOO	C41 H80 O6 N1	17.7900324	682.5991125	POS
Cer(t40:1;O)	Cer	M-H	C40 H78 O5 N1	16.66363267	652.5885475	NEG
Cer(t40:2)	Cer	M+HCOO	C41 H78 O6 N1	17.02654621	680.5834625	POS
Cer(t40:3)	Cer	M+CH3COO	C42 H78 O6 N1	12.85441944	692.5834625	POS
Cer(t41:1)	Cer	M-H	C41 H80 O4 N1	18.22789118	650.6092825	NEG
Cer(t41:3)	Cer	M+HCOO	C42 H78 O6 N1	17.45	692.5834625	POS
Cer(t42:0;O)	Cer	M+HCOO	C43 H86 O7 N1	17.884	728.6409775	POS
Cer(t42:1;O)	Cer	M+HCOO	C43 H84 O7 N1	17.13522947	726.6253275	POS
Cer(t42:2)	Cer	M+HCOO	C43 H82 O6 N1	18.49419579	708.6147625	POS
Cer(t42:5)	Cer	M+HCOO	C43 H76 O6 N1	16.534	702.5678125	POS
Cer(t42:7)	Cer	M+HCOO	C43 H72 O6 N1	14.817	698.5365125	POS
Cer(t43:0;O)	Cer	M+HCOO	C44 H88 O7 N1	18.6897158	742.6566275	POS
Cer(t43:1;O)	Cer	M+HCOO	C44 H86 O7 N1	18.20230685	740.6409775	POS
Cer(t43:2)	Cer	M+HCOO	C44 H84 O6 N1	18.4613217	722.6304125	POS
Cer(t43:4)	Cer	M+CH3COO	C45 H82 O6 N1	16.508	732.6147625	POS
Cer(t43:5)	Cer	M+CH3COO	C45 H80 O6 N1	15.428	730.5991125	POS
Cer(t44:1;O)	Cer	M-H	C44 H86 O5 N1	18.57250326	708.6511475	NEG
Cer(t44:2)	Cer	M-H	C44 H84 O4 N1	18.82226949	690.6405825	NEG
Cer(t44:5)	Cer	M-H	C44 H78 O4 N1	14.164	684.5936325	NEG

Cer(t44:6)	Cer	M-H	C44 H76 O4 N1	13.43574302	682.5779825	NEG
Cer(t45:2)	Cer	M+CH3COO	C47 H90 O6 N1	18.279	764.6773625	POS
CerG2GNAc1(m42:1)	CerG2GNAc1	M+CH3COO	C64 H119 O19 N2	13.172	1219.841257	POS
CerP(d41:7)	CerP	M-H	C41 H69 O6 N1 P1	9.932	702.4868005	NEG
CerP(d43:8)	CerP	M-H	C43 H71 O6 N1 P1	9.310191537	728.5024505	NEG
CerP(m32:2)	CerP	M-H	C32 H61 O5 N1 P1	11.101	570.4292855	NEG
CerP(m34:2)	CerP	M-H	C34 H65 O5 N1 P1	12.61596861	598.4605855	NEG
CerP(t34:0;2O)	CerP	M+CH3COO	C36 H73 O11 N1 P1	8.998	726.4926755	POS
CerP(t34:3)	CerP	M+CH3COO	C36 H67 O9 N1 P1	4.589	688.4558955	POS
CerP(t36:4)	CerP	M+CH3COO	C38 H69 O9 N1 P1	3.68	714.4715455	POS
CerP(t36:5)	CerP	M+CH3COO	C38 H67 O9 N1 P1	3.698733724	712.4558955	POS
ChE(11:0)	ChE	M+H	C38 H67 O2	15.57340318	555.5135565	POS
ChE(19:1)	ChE	M+NH4	C46 H84 O2 N1	19.393	682.6496555	POS
ChE(2:0)	ChE	M+H	C29 H49 O2	9.88	429.3727065	POS
ChE(36:0)	ChE	M+H	C63 H117 O2	20.409	905.9048065	POS
CmE(18:2)	CmE	M+H	C46 H79 O2	19.1	663.6074565	POS
CmE(18:3)	CmE	M+H	C46 H77 O2	18.6096567	661.5918065	POS
CmE(6:0)	CmE	M+H	C34 H59 O2	13.31699977	499.4509565	POS
CmE(8:0)	CmE	M+H	C36 H63 O2	19.255	527.4822565	POS
DG(10:0/10:2)	DG	M+NH4	C23 H44 O5 N1	3.08	414.3214005	POS
DG(10:0/12:2)	DG	M+H	C25 H45 O5	5.115295568	425.3261515	POS

DG(11:0/10:2)	DG	M+H	C24 H43 O5	4.719	411.3105015	POS
DG(11:0/11:2)	DG	M+H	C25 H45 O5	4.075	425.3261515	POS
DG(14:0/12:3)	DG	M+H	C29 H51 O5	18.00241446	479.3731015	POS
DG(14:0/18:2)	DG	M+NH4	C35 H68 O5 N1	14.35276515	582.5092005	POS
DG(14:0/18:3)	DG	M+NH4	C35 H66 O5 N1	13.26132117	580.4935505	POS
DG(15:0/16:0)	DG	M+Na	C34 H66 O5 Na1	10.68689791	577.4802465	POS
DG(15:0/18:2)	DG	M+NH4	C36 H70 O5 N1	15.01903753	596.5248505	POS
DG(15:0/18:3)	DG	M+H	C36 H65 O5	10.62484706	577.4826515	POS
DG(15:3)	DG	M+H	C18 H29 O5	5.33	325.2009515	POS
DG(16:0/10:1)	DG	M+Na	C29 H54 O5 Na1	15.066	505.3863465	POS
DG(16:0/10:2)	DG	M+H	C29 H53 O5	12.21233409	481.3887515	POS
DG(16:0/10:3)	DG	M+H	C29 H51 O5	7.933499279	479.3731015	POS
DG(16:0/11:2)	DG	M+H	C30 H55 O5	13.13917286	495.4044015	POS
DG(16:0/12:3)	DG	M+H	C31 H55 O5	18.282	507.4044015	POS
DG(16:0/12:4)	DG	M+H	C31 H53 O5	18.89897171	505.3887515	POS
DG(16:0/14:0)	DG	M+Na	C33 H64 O5 Na1	11.36	563.4645965	POS
DG(16:0/18:3)	DG	M+NH4	C37 H70 O5 N1	11.50603464	608.5248505	POS
DG(16:0/24:1)	DG	M+H	C43 H83 O5	17.91243332	679.6235015	POS
DG(16:1)	DG	M+Na	C19 H34 O5 Na1	2.093374994	365.2298465	POS
DG(16:1/12:3)	DG	M+H	C31 H53 O5	10.12107029	505.3887515	POS
DG(16:1/18:2)	DG	M+NH4	C37 H70 O5 N1	14.45297889	608.5248505	POS

DG(16:1/18:3)	DG	M+NH4	C37 H68 O5 N1	13.36501896	606.5092005	POS
DG(16:1e/16:0)	DG	M+Na	C35 H68 O4 Na1	13.147	575.5009815	POS
DG(17:0/18:3)	DG	M+H	C38 H69 O5	12.20526764	605.5139515	POS
DG(17:1/10:3)	DG	M+Na	C30 H50 O5 Na1	3.213	513.3550465	POS
DG(17:1/18:1)	DG	M+NH4	C38 H74 O5 N1	16.23344283	624.5561505	POS
DG(17:1/6:0)	DG	M+Na	C26 H48 O5 Na1	5.671	463.3393965	POS
DG(17:4)	DG	M+H	C20 H31 O5	1.534	351.2166015	POS
DG(18:0/18:1)	DG	M+NH4	C39 H78 O5 N1	17.88715436	640.5874505	POS
DG(18:0/18:2)	DG	M+H	C39 H73 O5	14.99943817	621.5452515	POS
DG(18:0/18:3)	DG	M+H	C39 H71 O5	16.04060943	619.5296015	POS
DG(18:1)	DG	M+NH4	C21 H42 O5 N1	2.302109271	388.3057505	POS
DG(18:1/10:2)	DG	M+H	C31 H55 O5	12.3457407	507.4044015	POS
DG(18:1/10:3)	DG	M+H	C31 H53 O5	17.1633507	505.3887515	POS
DG(18:1/11:2)	DG	M+H	C32 H57 O5	13.20806072	521.4200515	POS
DG(18:1/11:3)	DG	M+H	C32 H55 O5	8.540315089	519.4044015	POS
DG(18:1/12:3)	DG	M+NH4	C33 H60 O5 N1	11.44869393	550.4466005	POS
DG(18:1/12:4)	DG	M+H	C33 H55 O5	18.9221813	531.4044015	POS
DG(18:1/18:3)	DG	M+H	C39 H69 O5	11.80658762	617.5139515	POS
DG(18:1/20:4)	DG	M+NH4	C41 H74 O5 N1	15.427	660.5561505	POS
DG(18:1/22:0)	DG	M+NH4	C43 H86 O5 N1	19.38728973	696.6500505	POS
DG(18:1/24:0)	DG	M+NH4	C45 H90 O5 N1	19.93684435	724.6813505	POS

DG(18:2)	DG	M+NH4	C21 H40 O5 N1	1.8033124<br>13	386.29010<br>05	POS
DG(18:2/10:2)	DG	M+H	C31 H53 O5	15.765	505.38875<br>15	POS
DG(18:2/10:3)	DG	M+H	C31 H51 O5	16.194	503.37310<br>15	POS
DG(18:2/11:2)	DG	M+H	C32 H55 O5	12.169467<br>21	519.40440<br>15	POS
DG(18:2/11:3)	DG	M+H	C32 H53 O5	6.857	517.38875<br>15	POS
DG(18:2/12:3)	DG	M+H	C33 H55 O5	15.892	531.40440<br>15	POS
DG(18:2/12:4)	DG	M+H	C33 H53 O5	17.653	529.38875<br>15	POS
DG(18:2/18:2)	DG	M+H	C39 H69 O5	14.667137<br>7	617.51395<br>15	POS
DG(18:2e)	DG	M+H	C21 H39 O4	12.58	355.28428<br>65	POS
DG(18:3/10:1)	DG	M+H	C31 H53 O5	15.066	505.38875<br>15	POS
DG(18:3/10:2)	DG	M+H	C31 H51 O5	15.895287<br>45	503.37310<br>15	POS
DG(18:3/11:1)	DG	M+H	C32 H55 O5	9.0027275<br>03	519.40440<br>15	POS
DG(18:3/12:3)	DG	M+H	C33 H53 O5	9.4570250<br>71	529.38875<br>15	POS
DG(18:3/12:4)	DG	M+H	C33 H51 O5	13.968223<br>74	527.37310<br>15	POS
DG(18:3/18:2)	DG	M+NH4	C39 H70 O5 N1	13.612185<br>76	632.52485<br>05	POS
DG(18:3/18:3)	DG	M+NH4	C39 H68 O5 N1	12.605607<br>22	630.50920<br>05	POS
DG(18:3e)	DG	M+H	C21 H37 O4	12.679	353.26863<br>65	POS
DG(18:4)	DG	M+H	C21 H33 O5	8.568	365.23225<br>15	POS
DG(18:4/14:0)	DG	M+H	C35 H61 O5	9.2791623<br>38	561.45135<br>15	POS
DG(18:4/16:0)	DG	M+H	C37 H65 O5	11.809290<br>64	589.48265<br>15	POS
DG(18:4/18:0)	DG	M+H	C39 H69 O5	12.111739	617.51395<br>15	POS

DG(18:4/18:1)	DG	M+H	C39 H67 O5	19.065	615.4983015	POS
DG(18:4/18:2)	DG	M+H	C39 H65 O5	10.828	613.4826515	POS
DG(18:4/18:3)	DG	M+H	C39 H63 O5	17.714	611.4670015	POS
DG(18:4/6:0)	DG	M+NH4	C27 H48 O5 N1	3.694047414	466.3527005	POS
DG(18:4e)	DG	M+H	C21 H35 O4	4.119	351.2529865	POS
DG(18:5e)	DG	M+H	C21 H33 O4	9.723	349.2373365	POS
DG(19:2e)	DG	M+H	C22 H41 O4	10.43	369.2999365	POS
DG(19:3)	DG	M+H	C22 H37 O5	2.550359384	381.2635515	POS
DG(19:4e)	DG	M+H	C22 H37 O4	15.97768412	365.2686365	POS
DG(20:0)	DG	M+Na	C23 H44 O5 Na1	6.481498117	423.3080965	POS
DG(20:0/18:1)	DG	M+NH4	C41 H82 O5 N1	18.68135015	668.6187505	POS
DG(20:0/18:2)	DG	M+NH4	C41 H80 O5 N1	17.14	666.6031005	POS
DG(20:0/18:3)	DG	M+H	C41 H75 O5	14.07779329	647.5609015	POS
DG(20:1/18:1)	DG	M+NH4	C41 H80 O5 N1	17.82102046	666.6031005	POS
DG(20:1/18:2)	DG	M+H	C41 H75 O5	16.93637872	647.5609015	POS
DG(20:1/18:3)	DG	M+H	C41 H73 O5	12.291	645.5452515	POS
DG(20:2)	DG	M+Na	C23 H40 O5 Na1	3.673	419.2767965	POS
DG(20:2e)	DG	M+Na	C23 H42 O4 Na1	4.49	405.2975315	POS
DG(20:3/18:2)	DG	M+NH4	C41 H74 O5 N1	14.92376445	660.5561505	POS
DG(20:3e)	DG	M+H	C23 H41 O4	3.284773007	381.2999365	POS
DG(20:4/14:4)	DG	M+Na	C37 H56 O5 Na1	8.066	603.4019965	POS

DG(20:4e)	DG	M+H	C23 H39 O4	3.226	379.2842865	POS
DG(21:0)	DG	M+Na	C24 H46 O5 Na1	4.485784756	437.3237465	POS
DG(21:2e)	DG	M+H	C24 H45 O4	4.775094832	397.3312365	POS
DG(21:3e)	DG	M+H	C24 H43 O4	3.48440076	395.3155865	POS
DG(21:4e)	DG	M+H	C24 H41 O4	3.324	393.2999365	POS
DG(22:0/18:2)	DG	M+NH4	C43 H84 O5 N1	18.78544951	694.6344005	POS
DG(22:0/18:3)	DG	M+H	C43 H79 O5	15.543	675.5922015	POS
DG(22:0e)	DG	M+Na	C25 H50 O4 Na1	11.439	437.3601315	POS
DG(22:1)	DG	M+Na	C25 H46 O5 Na1	3.886	449.3237465	POS
DG(22:1/18:2)	DG	M+NH4	C43 H82 O5 N1	17.91864407	692.6187505	POS
DG(22:1e)	DG	M+Na	C25 H48 O4 Na1	10.307	435.3444815	POS
DG(22:2)	DG	M+Na	C25 H44 O5 Na1	4.536	447.3080965	POS
DG(22:2e)	DG	M+Na	C25 H46 O4 Na1	5.98	433.3288315	POS
DG(22:3)	DG	M+H	C25 H43 O5	2.518	423.3105015	POS
DG(22:3e)	DG	M+H	C25 H45 O4	5.133585201	409.3312365	POS
DG(22:4)	DG	M+Na	C25 H40 O5 Na1	1.502	443.2767965	POS
DG(22:4e)	DG	M+H	C25 H43 O4	4.118432359	407.3155865	POS
DG(23:0)	DG	M+Na	C26 H50 O5 Na1	10.15	465.3550465	POS
DG(23:1)	DG	M+NH4	C26 H52 O5 N1	7.51	458.3840005	POS
DG(23:2)	DG	M+H	C26 H47 O5	10.13282081	439.3418015	POS
DG(23:2e)	DG	M+NH4	C26 H52 O4 N1	9.58222287	442.3890855	POS

DG(23:3e)	DG	M+H	C26 H47 O4	5.533	423.3468865	POS
DG(23:4e)	DG	M+H	C26 H45 O4	2.506199317	421.3312365	POS
DG(24:0/18:2)	DG	M+NH4	C45 H88 O5 N1	19.45118126	722.6657005	POS
DG(24:0/18:3)	DG	M+H	C45 H83 O5	16.54075673	703.6235015	POS
DG(24:0/24:0)	DG	M+NH4	C51 H104 O5 N1	21.547	810.7909005	POS
DG(24:1)	DG	M+H	C27 H51 O5	6.595	455.3731015	POS
DG(24:1/18:2)	DG	M+H	C45 H83 O5	17.1358063	703.6235015	POS
DG(24:1e)	DG	M+H	C27 H53 O4	7.279	441.3938365	POS
DG(24:2)	DG	M+H	C27 H49 O5	10.84	453.3574515	POS
DG(24:2e)	DG	M+Na	C27 H50 O4 Na1	10.854	461.3601315	POS
DG(24:3)	DG	M+H	C27 H47 O5	9.841718932	451.3418015	POS
DG(24:3e)	DG	M+H	C27 H49 O4	8.860854764	437.3625365	POS
DG(24:4)	DG	M+Na	C27 H44 O5 Na1	3.126	471.3080965	POS
DG(24:4e)	DG	M+H	C27 H47 O4	10.31782738	435.3468865	POS
DG(24:5e)	DG	M+H	C27 H45 O4	5.612	433.3312365	POS
DG(25:0)	DG	M+Na	C28 H54 O5 Na1	17.462	493.3863465	POS
DG(25:0/18:2)	DG	M+NH4	C46 H90 O5 N1	19.77309219	736.6813505	POS
DG(25:0/6:0)	DG	M+Na	C34 H66 O5 Na1	9.275892649	577.4802465	POS
DG(25:1)	DG	M+Na	C28 H52 O5 Na1	17.76622647	491.3706965	POS
DG(25:2)	DG	M+H	C28 H51 O5	8.01800596	467.3731015	POS
DG(25:2e)	DG	M+Na	C28 H52 O4 Na1	18.813	475.3757815	POS

DG(25:3)	DG	M+H	C28 H49 O5	11.033	465.3574515	POS
DG(25:3e)	DG	M+Na	C28 H50 O4 Na1	9.12	473.3601315	POS
DG(25:4e)	DG	M+H	C28 H49 O4	9.133547501	449.3625365	POS
DG(25:5e)	DG	M+H	C28 H47 O4	7.546	447.3468865	POS
DG(26:0)	DG	M+Na	C29 H56 O5 Na1	12.568	507.4019965	POS
DG(26:0/16:0)	DG	M+NH4	C45 H92 O5 N1	20.41	726.6970005	POS
DG(26:0/18:1)	DG	M+NH4	C47 H94 O5 N1	20.39716219	752.7126505	POS
DG(26:0/18:2)	DG	M+NH4	C47 H92 O5 N1	19.98333171	750.6970005	POS
DG(26:1)	DG	M+Na	C29 H54 O5 Na1	10.13	505.3863465	POS
DG(26:1e)	DG	M+H	C29 H57 O4	8.898	469.4251365	POS
DG(26:2)	DG	M+Na	C29 H52 O5 Na1	17.369	503.3706965	POS
DG(26:3)	DG	M+H	C29 H51 O5	10.91633159	479.3731015	POS
DG(26:3e)	DG	M+H	C29 H53 O4	27.853	465.3938365	POS
DG(26:4e)	DG	M+H	C29 H51 O4	10	463.3781865	POS
DG(26:5e)	DG	M+H	C29 H49 O4	8.791	461.3625365	POS
DG(26:6e)	DG	M+H	C29 H47 O4	2.885	459.3468865	POS
DG(27:0)	DG	M+Na	C30 H58 O5 Na1	12.90332043	521.4176465	POS
DG(27:0e)	DG	M+Na	C30 H60 O4 Na1	9.97	507.4383815	POS
DG(27:1)	DG	M+Na	C30 H56 O5 Na1	17.603	519.4019965	POS
DG(27:2)	DG	M+Na	C30 H54 O5 Na1	15.508	517.3863465	POS
DG(27:2e)	DG	M+Na	C30 H56 O4 Na1	8.443190473	503.4070815	POS

DG(27:3)	DG	M+H	C30 H53 O5	20.027	493.38875 15	POS
DG(27:3e)	DG	M+Na	C30 H54 O4 Na1	17.608	501.39143 15	POS
DG(27:4)	DG	M+H	C30 H51 O5	13.607864 74	491.37310 15	POS
DG(27:4e)	DG	M+H	C30 H53 O4	8.299	477.39383 65	POS
DG(27:5)	DG	M+H	C30 H49 O5	15.835	489.35745 15	POS
DG(27:5e)	DG	M+H	C30 H51 O4	9.4576626 48	475.37818 65	POS
DG(27:6e)	DG	M+H	C30 H49 O4	18.173	473.36253 65	POS
DG(28:1)	DG	M+Na	C31 H58 O5 Na1	11.478	533.41764 65	POS
DG(28:1e)	DG	M+Na	C31 H60 O4 Na1	9.632	519.43838 15	POS
DG(28:2)	DG	M+Na	C31 H56 O5 Na1	17.62	531.40199 65	POS
DG(28:3)	DG	M+Na	C31 H54 O5 Na1	9.463	529.38634 65	POS
DG(28:5)	DG	M+H	C31 H51 O5	11.357	503.37310 15	POS
DG(28:6e)	DG	M+H	C31 H51 O4	11.142	487.37818 65	POS
DG(29:0)	DG	M+H	C32 H63 O5	11.045	527.46700 15	POS
DG(29:1)	DG	M+H	C32 H61 O5	10.191	525.45135 15	POS
DG(29:1e)	DG	M+Na	C32 H62 O4 Na1	14.479	533.45403 15	POS
DG(29:2)	DG	M+H	C32 H59 O5	14.238	523.43570 15	POS
DG(29:2e)	DG	M+H	C32 H61 O4	11.049	509.45643 65	POS
DG(29:3)	DG	M+H	C32 H57 O5	8.3642100 07	521.42005 15	POS
DG(29:3e)	DG	M+H	C32 H59 O4	9.958	507.44078 65	POS
DG(29:4)	DG	M+H	C32 H55 O5	8.232	519.40440 15	POS

DG(29:5)	DG	M+H	C32 H53 O5	5.869	517.3887515	POS
DG(29:5e)	DG	M+H	C32 H55 O4	8.44	503.4094865	POS
DG(29:6e)	DG	M+H	C32 H53 O4	16.711	501.3938365	POS
DG(30:0e)	DG	M+Na	C33 H66 O4 Na1	20.43	549.4853315	POS
DG(30:1e)	DG	M+Na	C33 H64 O4 Na1	14.2850568	547.4696815	POS
DG(30:3)	DG	M+NH4	C33 H62 O5 N1	10.723	552.4622505	POS
DG(30:3e)	DG	M+H	C33 H61 O4	20.405	521.4564365	POS
DG(30:4)	DG	M+H	C33 H57 O5	15.319	533.4200515	POS
DG(30:5)	DG	M+H	C33 H55 O5	17.434	531.4044015	POS
DG(30:5e)	DG	M+H	C33 H57 O4	19.619	517.4251365	POS
DG(30:6e)	DG	M+H	C33 H55 O4	18.77181699	515.4094865	POS
DG(31:0)	DG	M+Na	C34 H66 O5 Na1	10.314	577.4802465	POS
DG(31:1)	DG	M+NH4	C34 H68 O5 N1	11.7644182	570.5092005	POS
DG(31:1e)	DG	M+Na	C34 H66 O4 Na1	20.33455626	561.4853315	POS
DG(31:2e)	DG	M+Na	C34 H64 O4 Na1	9.510776529	559.4696815	POS
DG(31:3e)	DG	M+H	C34 H63 O4	9.923	535.4720865	POS
DG(31:4e)	DG	M+H	C34 H61 O4	9.180334715	533.4564365	POS
DG(32:0/18:1)	DG	M+NH4	C53 H106 O5 N1	21.73	836.8065505	POS
DG(32:1)	DG	M+Na	C35 H66 O5 Na1	15.767	589.4802465	POS
DG(32:1e)	DG	M+Na	C35 H68 O4 Na1	12.233	575.5009815	POS
DG(32:2e)	DG	M+Na	C35 H66 O4 Na1	20.163	573.4853315	POS

DG(32:3e)	DG	M+H	C35 H65 O4	20.435	549.4877365	POS
DG(32:4e)	DG	M+H	C35 H63 O4	11.688	547.4720865	POS
DG(32:5e)	DG	M+H	C35 H61 O4	13.25	545.4564365	POS
DG(32:8)	DG	M+H	C35 H53 O5	13.19746646	553.3887515	POS
DG(33:0)	DG	M+Na	C36 H70 O5 Na1	14.023	605.5115465	POS
DG(33:0e)	DG	M+Na	C36 H72 O4 Na1	21.403	591.5322815	POS
DG(33:1)	DG	M+Na	C36 H68 O5 Na1	12.96283378	603.4958965	POS
DG(33:1e)	DG	M+Na	C36 H70 O4 Na1	21.092	589.5166315	POS
DG(33:2)	DG	M+NH4	C36 H70 O5 N1	11.42685841	596.5248505	POS
DG(33:2e)	DG	M+Na	C36 H68 O4 Na1	20.6798777	587.5009815	POS
DG(33:3)	DG	M+NH4	C36 H68 O5 N1	10.62484706	594.5092005	POS
DG(33:3e)	DG	M+H	C36 H67 O4	15.49277628	563.5033865	POS
DG(33:4)	DG	M+H	C36 H63 O5	9.819542202	575.4670015	POS
DG(33:4e)	DG	M+H	C36 H65 O4	13.66174947	561.4877365	POS
DG(34:1)	DG	M+Na	C37 H70 O5 Na1	16.297	617.5115465	POS
DG(34:2e)	DG	M+Na	C37 H70 O4 Na1	20.82331914	601.5166315	POS
DG(34:3)	DG	M+Na	C37 H66 O5 Na1	17.656	613.4802465	POS
DG(34:4)	DG	M+Na	C37 H64 O5 Na1	16.947	611.4645965	POS
DG(34:5)	DG	M+H	C37 H63 O5	14.291	587.4670015	POS
DG(34:5e)	DG	M+H	C37 H65 O4	18.92379408	573.4877365	POS
DG(34:6e)	DG	M+H	C37 H63 O4	11.35144564	571.4720865	POS

DG(35:2)	DG	M+NH4	C38 H74 O5 N1	15.418	624.5561505	POS
DG(35:3)	DG	M+H	C38 H69 O5	14.598	605.5139515	POS
DG(35:4)	DG	M+H	C38 H67 O5	10.39883326	603.4983015	POS
DG(35:4e)	DG	M+H	C38 H69 O4	12.483	589.5190365	POS
DG(36:1e)	DG	M+Na	C39 H76 O4 Na1	19.88592725	631.5635815	POS
DG(36:2e)	DG	M+Na	C39 H74 O4 Na1	21.244	629.5479315	POS
DG(36:3)	DG	M+H	C39 H71 O5	12.50074813	619.5296015	POS
DG(36:3e)	DG	M+H	C39 H73 O4	21.55948476	605.5503365	POS
DG(36:5e)	DG	M+H	C39 H69 O4	19.44946875	601.5190365	POS
DG(36:6)	DG	M+H	C39 H65 O5	9.762	613.4826515	POS
DG(36:6e)	DG	M+H	C39 H67 O4	18.92621871	599.5033865	POS
DG(36:7e)	DG	M+H	C39 H65 O4	18.434	597.4877365	POS
DG(37:1)	DG	M+NH4	C40 H80 O5 N1	17.111	654.6031005	POS
DG(37:2)	DG	M+NH4	C40 H78 O5 N1	16.16	652.5874505	POS
DG(37:4)	DG	M+NH4	C40 H74 O5 N1	14.087	648.5561505	POS
DG(37:4e)	DG	M+H	C40 H73 O4	21.414	617.5503365	POS
DG(38:0)	DG	M+Na	C41 H80 O5 Na1	15.549	675.5897965	POS
DG(38:2e)	DG	M+H	C41 H79 O4	22.162	635.5972865	POS
DG(38:3)	DG	M+H	C41 H75 O5	13.742	647.5609015	POS
DG(38:3e)	DG	M+H	C41 H77 O4	18.63375799	633.5816365	POS
DG(38:4e)	DG	M+H	C41 H75 O4	21.51414588	631.5659865	POS

DG(38:5e)	DG	M+H	C41 H73 O4	16.891	629.5503365	POS
DG(38:6e)	DG	M+H	C41 H71 O4	15.991	627.5346865	POS
DG(39:3e)	DG	M+H	C42 H79 O4	18.05344611	647.5972865	POS
DG(40:0)	DG	M+Na	C43 H84 O5 Na1	16.751	703.6210965	POS
DG(40:3e)	DG	M+H	C43 H81 O4	22.14	661.6129365	POS
DG(40:4)	DG	M+NH4	C43 H80 O5 N1	17.104	690.6031005	POS
DG(41:2)	DG	M+Na	C44 H82 O5 Na1	15.056	713.6054465	POS
DG(42:4)	DG	M+Na	C45 H80 O5 Na1	16.889	723.5897965	POS
DG(42:7e)	DG	M+Na	C45 H76 O4 Na1	17.442	703.5635815	POS
DG(43:3e)	DG	M+H	C46 H87 O4	19.46999028	703.6598865	POS
DG(47:1)	DG	M+NH4	C50 H100 O5 N1	20.647	794.7596005	POS
DG(56:2)	DG	M+Na	C59 H112 O5 Na1	20.868	923.8401965	POS
DG(6:0/10:1)	DG	M+NH4	C19 H38 O5 N1	2.093374994	360.2744505	POS
DG(6:0/10:2)	DG	M+H	C19 H33 O5	1.875895712	341.2322515	POS
DG(6:0/12:1)	DG	M+H	C21 H39 O5	2.302109271	371.2792015	POS
DG(6:0/12:2)	DG	M+H	C21 H37 O5	1.803312413	369.2635515	POS
DG(6:0/12:3)	DG	M+H	C21 H35 O5	10.51568855	367.2479015	POS
DG(6:0/12:4)	DG	M+H	C21 H33 O5	16.401	365.2322515	POS
DG(6:0/18:2)	DG	M+NH4	C27 H52 O5 N1	4.843	470.3840005	POS
DG(6:0/18:3)	DG	M+H	C27 H47 O5	7.496744165	451.3418015	POS
DG(6:0/20:4)	DG	M+H	C29 H49 O5	11.37849156	477.3574515	POS

DG(6:0/20:5)	DG	M+H	C29 H47 O5	7.834994085	475.3418015	POS
DG(6:0/22:4)	DG	M+H	C31 H53 O5	11.24249993	505.3887515	POS
DG(8:0/10:2)	DG	M+H	C21 H37 O5	1.327	369.2635515	POS
DG(8:0/10:3)	DG	M+H	C21 H35 O5	10.956	367.2479015	POS
DG(8:0/18:3)	DG	M+H	C29 H51 O5	6.706070313	479.3731015	POS
DG(8:0/20:4)	DG	M+H	C31 H53 O5	14.60822473	505.3887515	POS
DG(8:0/22:6)	DG	M+H	C33 H53 O5	15.61533081	529.3887515	POS
DG(8:0/8:0)	DG	M+Na	C19 H36 O5 Na1	8.931	367.2454965	POS
DG(9:0/10:1)	DG	M+H	C22 H41 O5	2.391668014	385.2948515	POS
DG(9:0/9:0)	DG	M+NH4	C21 H44 O5 N1	2.957295663	390.3214005	POS
DGDG(16:0/18:2)	DGDG	M+NH4	C49 H92 O15 N1	13.47281405	934.6461505	POS
DGDG(16:0/18:3)	DGDG	M+HCOO	C50 H87 O17	12.44588974	959.5948785	POS
DGDG(18:2/18:2)	DGDG	M+NH4	C51 H92 O15 N1	12.36786231	958.6461505	POS
DGDG(18:2/18:3)	DGDG	M+HCOO	C52 H87 O17	11.36167121	983.5948785	POS
DGDG(18:3/18:3)	DGDG	M+HCOO	C52 H85 O17	10.38626725	981.5792285	POS
DGDG(28:2e)	DGDG	M-H	C43 H77 O14	16.00392938	817.5318835	NEG
DGDG(30:1)	DGDG	M+Na	C45 H82 O15 Na1	14.923	885.5545965	POS
DGDG(30:2)	DGDG	M-H	C45 H79 O15	7.993769881	859.5424485	NEG
DGDG(32:2)	DGDG	M+CH3COO	C49 H87 O17	9.316	947.5948785	POS
DGDG(36:1e)	DGDG	M+HCOO	C52 H97 O16	14.019	977.6782135	POS
DGDG(38:1)	DGDG	M+HCOO	C54 H99 O17	15.783	1019.688779	POS

DGDG(41:3)	DGDG	M-H	C56 H99 O15	11.22695909	1011.698949	NEG
DGDG(44:2)	DGDG	M-H	C59 H107 O15	14.3185679	1055.761549	NEG
DGDG(45:2)	DGDG	M-H	C60 H109 O15	17.25582344	1069.777199	NEG
DGMG(16:0)	DGMG	M+HCOO	C32 H59 O16	3.562763822	699.3808635	POS
DGMG(18:1)	DGMG	M+HCOO	C34 H61 O16	3.82	725.3965135	POS
DGMG(18:2)	DGMG	M+HCOO	C34 H59 O16	2.780089175	723.3808635	POS
DLCL(29:0)	DLCL	M-H	C38 H75 O15 P2	13.21561842	833.4586745	NEG
DLCL(29:1)	DLCL	M-H	C38 H73 O15 P2	12.043	831.4430245	NEG
DLCL(34:3)	DLCL	M-H	C43 H79 O15 P2	6.679	897.4899745	NEG
FA(18:2)	FA	M-H	O2 H31 C18	11.44596009	279.2329535	NEG
FA(18:3)	FA	M-H	O2 H29 C18	4.562338944	277.2173035	NEG
FA(18:4)	FA	M-H	O2 H27 C18	1.795	275.2016535	NEG
GM3(m37:0)	GM3	M-H	C60 H111 O20 N2	10.469	1179.773572	NEG
GM3(m41:0)	GM3	M-H	C64 H119 O20 N2	13.07528687	1235.836172	NEG
Hex1Cer(d13:0/16:0)	Hex1Cer	M-H	C35 H68 O8 N1	9.921	630.4950425	NEG
Hex1Cer(d18:2/18:1)	Hex1Cer	M+H	C42 H78 O8 N1	13.74	724.5721955	POS
Hex1Cer(d18:2/20:0;O)	Hex1Cer	M+H	C44 H84 O9 N1	15.15840729	770.6140605	POS
Hex1Cer(d18:2/20:1)	Hex1Cer	M+H	C44 H82 O8 N1	15.1596513	752.6034955	POS
Hex1Cer(d18:2/22:0;O)	Hex1Cer	M+H	C46 H88 O9 N1	16.475	798.6453605	POS
Hex1Cer(d18:2/24:1)	Hex1Cer	M+H	C48 H90 O8 N1	17.556	808.6660955	POS
Hex1Cer(d28:0)	Hex1Cer	M-H	C34 H66 O8 N1	12.609	616.4793925	NEG

Hex1Cer(d30:2)	Hex1Cer	M-H	C36 H66 O8 N1	11.851	640.4793925	NEG
Hex1Cer(d31:1)	Hex1Cer	M+HCOO	C38 H72 O10 N1	10.571	702.5161725	POS
Hex1Cer(d32:1)	Hex1Cer	M+HCOO	C39 H74 O10 N1	12.38849427	716.5318225	POS
Hex1Cer(d32:2)	Hex1Cer	M+HCOO	C39 H72 O10 N1	11.2109168	714.5161725	POS
Hex1Cer(d34:2)	Hex1Cer	M+HCOO	C41 H76 O10 N1	11.65142853	742.5474725	POS
Hex1Cer(d34:3)	Hex1Cer	M+HCOO	C41 H74 O10 N1	14.223	740.5318225	POS
Hex1Cer(d34:4)	Hex1Cer	M+HCOO	C41 H72 O10 N1	12.751	738.5161725	POS
Hex1Cer(d36:1)	Hex1Cer	M+CH3COO	C44 H84 O10 N1	14.678	786.6100725	POS
Hex1Cer(d37:1)	Hex1Cer	M+HCOO	C44 H84 O10 N1	14.79	786.6100725	POS
Hex1Cer(d37:2)	Hex1Cer	M+H	C43 H82 O8 N1	2.378	740.6034955	POS
Hex1Cer(d38:1)	Hex1Cer	M+CH3COO	C46 H88 O10 N1	16.003	814.6413725	POS
Hex1Cer(d38:1;O)	Hex1Cer	M+HCOO	C45 H86 O11 N1	16.06339133	816.6206375	POS
Hex1Cer(d38:2)	Hex1Cer	M+HCOO	C45 H84 O10 N1	15.698	798.6100725	POS
Hex1Cer(d39:1)	Hex1Cer	M+HCOO	C46 H88 O10 N1	15.99172355	814.6413725	POS
Hex1Cer(d40:1)	Hex1Cer	M+CH3COO	C48 H92 O10 N1	17.172	842.6726725	POS
Hex1Cer(d40:3)	Hex1Cer	M-H	C46 H84 O8 N1	16.53079153	778.6202425	NEG
Hex1Cer(d42:1)	Hex1Cer	M+CH3COO	C50 H96 O10 N1	18.09181033	870.7039725	POS
Hex1Cer(d43:1)	Hex1Cer	M+HCOO	C50 H96 O10 N1	18.09277685	870.7039725	POS
Hex1Cer(d46:2)	Hex1Cer	M-H	C52 H98 O8 N1	19.59324315	864.7297925	NEG
Hex1Cer(d48:2)	Hex1Cer	M-H	C54 H102 O8 N1	20.194	892.7610925	NEG
Hex1Cer(m20:1/18:1)	Hex1Cer	M+H	C44 H84 O7 N1	15.163	738.6242305	POS

Hex1Cer(m39:2)	Hex1Cer	M+HCOO	C46 H86 O9 N1	16.48	796.6308075	POS
Hex1Cer(m40:2)	Hex1Cer	M+CH3COO	C48 H90 O9 N1	17.55076626	824.6621075	POS
Hex1Cer(t18:0/18:2)	Hex1Cer	M-H	C42 H78 O9 N1	13.75567748	740.5682075	NEG
Hex1Cer(t18:1/22:0;O)	Hex1Cer	M+H	C46 H90 O10 N1	15.9552832	816.6559255	POS
Hex1Cer(t18:1/23:0;O)	Hex1Cer	M+H	C47 H92 O10 N1	16.56974536	830.6715755	POS
Hex1Cer(t18:1/24:0;O)	Hex1Cer	M+H	C48 H94 O10 N1	17.15040806	844.6872255	POS
Hex1Cer(t18:1/26:0;O)	Hex1Cer	M+H	C50 H98 O10 N1	17.157	872.7185255	POS
Hex1Cer(t20:0/18:2)	Hex1Cer	M+HCOO	C45 H84 O11 N1	15.18033471	814.6049875	POS
Hex1Cer(t36:2)	Hex1Cer	M+CH3COO	C44 H82 O11 N1	14.47	800.5893375	POS
Hex1Cer(t36:3)	Hex1Cer	M+CH3COO	C44 H80 O11 N1	13.82674198	798.5736875	POS
Hex1Cer(t38:2)	Hex1Cer	M+CH3COO	C46 H86 O11 N1	15.825	828.6206375	POS
Hex1Cer(t38:3)	Hex1Cer	M+CH3COO	C46 H84 O11 N1	14.894	826.6049875	POS
Hex1Cer(t40:1;O)	Hex1Cer	M+HCOO	C47 H90 O12 N1	15.9552832	860.6468525	POS
Hex1Cer(t40:2)	Hex1Cer	M+HCOO	C47 H88 O11 N1	16.474	842.6362875	POS
Hex1Cer(t42:1;O)	Hex1Cer	M+HCOO	C49 H94 O12 N1	17.15040806	888.6781525	POS
Hex1Cer(t42:2)	Hex1Cer	M+HCOO	C49 H92 O11 N1	17.58405717	870.6675875	POS
Hex1Cer(t44:1;O)	Hex1Cer	M+HCOO	C51 H98 O12 N1	18.12	916.7094525	POS
Hex2Cer(d33:2)	Hex2Cer	M-H	C45 H82 O13 N1	10.506	844.5791675	NEG
Hex2Cer(d33:3)	Hex2Cer	M-H	C45 H80 O13 N1	8.438	842.5635175	NEG
Hex2Cer(d36:4)	Hex2Cer	M-H	C48 H84 O13 N1	13.337	882.5948175	NEG
Hex2Cer(m34:0)	Hex2Cer	M-H	C46 H88 O12 N1	13.213	846.6312025	NEG

Hex2Cer(m35:1)	Hex2Cer	M-H	C47 H88 O12 N1	15.904	858.6312025	NEG
Hex2Cer(m35:2)	Hex2Cer	M-H	C47 H86 O12 N1	14.828	856.6155525	NEG
Hex2Cer(m35:3)	Hex2Cer	M-H	C47 H84 O12 N1	13.69	854.5999025	NEG
Hex2Cer(m36:0)	Hex2Cer	M-H	C48 H92 O12 N1	16.591	874.6625025	NEG
Hex2Cer(m37:0)	Hex2Cer	M-H	C49 H94 O12 N1	17.187	888.6781525	NEG
Hex2Cer(m40:2)	Hex2Cer	M+CH3COO	C54 H100 O14 N1	14.257	986.7149325	POS
Hex2Cer(m40:4)	Hex2Cer	M+CH3COO	C54 H96 O14 N1	11.389	982.6836325	POS
Hex2Cer(t43:1)	Hex2Cer	M+H	C55 H106 O14 N1	17.82057131	1004.760785	POS
LBPA(16:0/16:0)	LBPA	M-H	C38 H74 O10 P1	13.5970373	721.5025115	NEG
LBPA(16:0/18:0)	LBPA	M-H	C40 H78 O10 P1	14.91764733	749.5338115	NEG
LBPA(16:0/18:1)	LBPA	M+NH4	C40 H81 O10 P1 N1	13.66439673	766.5592635	POS
LBPA(16:0/18:2)	LBPA	M+NH4	C40 H79 O10 P1 N1	12.538	764.5436135	POS
LBPA(30:1)	LBPA	M-H	C36 H68 O10 P1	10.224	691.4555615	NEG
LBPA(32:1)	LBPA	M-H	C38 H72 O10 P1	8.623	719.4868615	NEG
LBPA(32:2)	LBPA	M-H	C38 H70 O10 P1	10.07168812	717.4712115	NEG
LBPA(32:3)	LBPA	M-H	C38 H68 O10 P1	8.346198436	715.4555615	NEG
LPA(15:0)	LPA	M-H	C18 H36 O7 N0 P1	5.935	395.2204165	NEG
LPA(16:0)	LPA	M-H	C19 H38 O7 N0 P1	4.541	409.2360665	NEG
LPA(16:2e)	LPA	M-H	C19 H36 O6 N0 P1	1.598	391.2255015	NEG
LPA(18:1)	LPA	M-H	C21 H40 O7 N0 P1	5.757365444	435.2517165	NEG
LPA(18:2)	LPA	M-H	C21 H38 O7 N0 P1	5.825633446	433.2360665	NEG

LPA(20:0)	LPA	M-H	C23 H46 O7 N0 P1	7.463	465.2986665	NEG
LPA(22:0)	LPA	M-H	C25 H50 O7 N0 P1	9.260718398	493.3299665	NEG
LPC(14:0)	LPC	M+H	C22 H47 O7 N1 P1	2.285731732	468.3084685	POS
LPC(15:0)	LPC	M+H	C23 H49 O7 N1 P1	1.245985255	482.3241185	POS
LPC(16:0)	LPC	M+HCOO	C25 H51 O9 N1 P1	3.569807711	540.3306955	POS
LPC(16:1)	LPC	M+H	C24 H49 O7 N1 P1	2.517	494.3241185	POS
LPC(18:0)	LPC	M+HCOO	C27 H55 O9 N1 P1	5.482475559	568.3619955	POS
LPC(18:1)	LPC	M+HCOO	C27 H53 O9 N1 P1	3.811993062	566.3463455	POS
LPC(18:2)	LPC	M+HCOO	C27 H51 O9 N1 P1	2.763599558	564.3306955	POS
LPC(18:3)	LPC	M+H	C26 H49 O7 N1 P1	2.133297889	518.3241185	POS
LPC(20:0)	LPC	M+H	C28 H59 O7 N1 P1	7.674718878	552.4023685	POS
LPC(20:1)	LPC	M+HCOO	C29 H57 O9 N1 P1	5.616795455	594.3776455	POS
LPC(22:4)	LPC	M+CH3COO	C32 H57 O9 N1 P1	5.405	630.3776455	POS
LPE(16:0)	LPE	M-H	C21 H43 O7 N1 P1	3.756480048	452.2782655	NEG
LPE(18:0)	LPE	M-H	C23 H47 O7 N1 P1	5.664198449	480.3095655	NEG
LPE(18:1)	LPE	M-H	C23 H45 O7 N1 P1	4.016342486	478.2939155	NEG
LPE(18:2)	LPE	M-H	C23 H43 O7 N1 P1	2.868556638	476.2782655	NEG
LPE(18:3)	LPE	M+H	C23 H43 O7 N1 P1	1.398	476.2771685	POS
LPE(20:1)	LPE	M-H	C25 H49 O7 N1 P1	5.929	506.3252155	NEG
LPG(16:0)	LPG	M-H	C22 H44 O9 N0 P1	2.933679274	483.2728465	NEG
LPG(18:1)	LPG	M-H	C24 H46 O9 N0 P1	3.296542222	509.2884965	NEG

LPG(18:2)	LPG	M-H	C24 H44 O9 N0 P1	2.32529727	507.2728465	NEG
LPG(18:4)	LPG	M-H	C24 H40 O9 N0 P1	4.427	503.2415465	NEG
LPI(15:0)	LPI	M-H	C24 H46 O12 N0 P1	1.115557084	557.2732415	NEG
LPI(16:0)	LPI	M-H	C25 H48 O12 N0 P1	2.787972852	571.2888915	NEG
LPI(18:0)	LPI	M-H	C27 H52 O12 N0 P1	4.35	599.3201915	NEG
LPI(18:1)	LPI	M-H	C27 H50 O12 N0 P1	2.793503627	597.3045415	NEG
LPI(18:2)	LPI	M-H	C27 H48 O12 N0 P1	2.072014819	595.2888915	NEG
LPMe(18:2)	LPMe	M-H	C22 H40 O7 N0 P1	2.717	447.2517165	NEG
LdMePE(16:0)	LdMePE	M-H	C23 H47 O7 N1 P1	3.593327333	480.3095655	NEG
LdMePE(18:1)	LdMePE	M-H	C25 H49 O7 N1 P1	3.530735868	506.3252155	NEG
LdMePE(18:2)	LdMePE	M-H	C25 H47 O7 N1 P1	2.769511358	504.3095655	NEG
MG(16:0)	MG	M+H	C19 H39 O4	27.913	331.2842865	POS
MG(18:1)	MG	M+H	C21 H41 O4	6.14923555	357.2999365	POS
MG(18:2)	MG	M+H	C21 H39 O4	12.581	355.2842865	POS
MG(18:3)	MG	M+H	C21 H37 O4	1.792268672	353.2686365	POS
MG(18:3e)	MG	M+H	C21 H39 O3	2.888	339.2893715	POS
MG(18:4)	MG	M+H	C21 H35 O4	11.36566888	351.2529865	POS
MG(20:4e)	MG	M+H	C23 H41 O3	4.226	365.3050215	POS
MGDG(14:0/14:0)	MGDG	M+HCOO	C38 H71 O12	12.26851045	719.4951035	POS
MGDG(16:0/16:0)	MGDG	M+HCOO	C42 H79 O12	15.46568059	775.5577035	POS
MGDG(16:0/18:1)	MGDG	M+HCOO	C44 H81 O12	14.80263462	801.5733535	POS

MGDG(16:0/18:2)	MGDG	M+HCOO	C44 H79 O12	14.41060555	799.5577035	POS
MGDG(16:0/18:3)	MGDG	M-H	C43 H75 O10	13.099	751.5365735	NEG
MGDG(18:1/18:1)	MGDG	M+HCOO	C46 H83 O12	15.50207138	827.5890035	POS
MGDG(18:1/18:2)	MGDG	M+HCOO	C46 H81 O12	14.42373266	825.5733535	POS
MGDG(18:2/18:2)	MGDG	M+HCOO	C46 H79 O12	13.29759833	823.5577035	POS
MGDG(18:2/18:3)	MGDG	M+NH4	C45 H80 O10 N1	12.24731841	794.5776755	POS
MGDG(18:3/18:3)	MGDG	M+HCOO	C46 H75 O12	11.25452687	819.5264035	POS
MGDG(24:1)	MGDG	M-H	C33 H59 O10	10.2330251	615.4113735	NEG
MGDG(24:2)	MGDG	M-H	C33 H57 O10	5.193408911	613.3957235	NEG
MGDG(26:0)	MGDG	M+HCOO	C36 H67 O12	9.77	691.4638035	POS
MGDG(26:2)	MGDG	M-H	C35 H61 O10	7.651846452	641.4270235	NEG
MGDG(27:0)	MGDG	M-H	C36 H67 O10	13.82	659.4739735	NEG
MGDG(27:2)	MGDG	M-H	C36 H63 O10	7.994890658	655.4426735	NEG
MGDG(28:0)	MGDG	M-H	C37 H69 O10	5.771	673.4896235	NEG
MGDG(28:1)	MGDG	M-H	C37 H67 O10	7.653	671.4739735	NEG
MGDG(28:1e)	MGDG	M+HCOO	C38 H71 O11	9.659	703.5001885	POS
MGDG(28:2)	MGDG	M-H	C37 H65 O10	9.009835334	669.4583235	NEG
MGDG(28:3e)	MGDG	M+CH3COO	C39 H69 O11	4.433	713.4845385	POS
MGDG(29:0)	MGDG	M-H	C38 H71 O10	8.438	687.5052735	NEG
MGDG(29:1)	MGDG	M-H	C38 H69 O10	9.922840429	685.4896235	NEG
MGDG(29:2)	MGDG	M-H	C38 H67 O10	9.416070613	683.4739735	NEG

MGDG(29:3)	MGDG	M-H	C38 H65 O10	8.108	681.4583235	NEG
MGDG(29:5)	MGDG	M-H	C38 H61 O10	7.999	677.4270235	NEG
MGDG(30:0)	MGDG	M-H	C39 H73 O10	11.473	701.5209235	NEG
MGDG(30:1)	MGDG	M-H	C39 H71 O10	5.368	699.5052735	NEG
MGDG(30:2)	MGDG	M-H	C39 H69 O10	7.547	697.4896235	NEG
MGDG(30:2e)	MGDG	M-H	C39 H71 O9	8.60968747	683.5103585	NEG
MGDG(30:3)	MGDG	M-H	C39 H67 O10	9.160829	695.4739735	NEG
MGDG(30:3e)	MGDG	M+HCOO	C40 H71 O11	7.777	727.5001885	POS
MGDG(30:4)	MGDG	M+HCOO	C40 H67 O12	13.124	739.4638035	POS
MGDG(31:1)	MGDG	M-H	C40 H73 O10	9.683	713.5209235	NEG
MGDG(31:2)	MGDG	M-H	C40 H71 O10	10.459	711.5052735	NEG
MGDG(31:3)	MGDG	M-H	C40 H69 O10	9.581	709.4896235	NEG
MGDG(31:4)	MGDG	M-H	C40 H67 O10	8.540739335	707.4739735	NEG
MGDG(31:6)	MGDG	M-H	C40 H63 O10	8.113	703.4426735	NEG
MGDG(32:1)	MGDG	M+CH3COO	C43 H79 O12	6.208	787.5577035	POS
MGDG(32:2)	MGDG	M+HCOO	C42 H75 O12	11.89254237	771.5264035	POS
MGDG(32:2e)	MGDG	M+HCOO	C42 H77 O11	11.328	757.5471385	POS
MGDG(32:3)	MGDG	M+HCOO	C42 H73 O12	10.91	769.5107535	POS
MGDG(32:3e)	MGDG	M+HCOO	C42 H75 O11	8.605029844	755.5314885	POS
MGDG(33:4)	MGDG	M+CH3COO	C44 H75 O12	8.553456997	795.5264035	POS
MGDG(33:5)	MGDG	M+CH3COO	C44 H73 O12	7.607	793.5107535	POS

MGDG(33:6)	MGDG	M+CH3COO	C44 H71 O12	6.333	791.4951035	POS
MGDG(34:1)	MGDG	M-H	C43 H79 O10	15.716	755.5678735	NEG
MGDG(34:2)	MGDG	M-H	C43 H77 O10	14.41060555	753.5522235	NEG
MGDG(34:3)	MGDG	M-H	C43 H75 O10	12.59002062	751.5365735	NEG
MGDG(34:3e)	MGDG	M-H	C43 H77 O9	10.395	737.5573085	NEG
MGDG(35:1)	MGDG	M-H	C44 H81 O10	16.524	769.5835235	NEG
MGDG(35:2)	MGDG	M-H	C44 H79 O10	14.01427985	767.5678735	NEG
MGDG(35:4)	MGDG	M+CH3COO	C46 H79 O12	8.012195414	823.5577035	POS
MGDG(35:5)	MGDG	M-H	C44 H73 O10	8.885	761.5209235	NEG
MGDG(35:6)	MGDG	M+HCOO	C45 H73 O12	9.757	805.5107535	POS
MGDG(36:1)	MGDG	M-H	C45 H83 O10	19.775	783.5991735	NEG
MGDG(36:2)	MGDG	M-H	C45 H81 O10	14.6899717	781.5835235	NEG
MGDG(36:2e)	MGDG	M-H	C45 H83 O9	15.95538709	767.6042585	NEG
MGDG(36:3e)	MGDG	M-H	C45 H81 O9	12.422	765.5886085	NEG
MGDG(36:4)	MGDG	M+CH3COO	C47 H81 O12	10.275	837.5733535	POS
MGDG(37:2)	MGDG	M-H	C46 H83 O10	15.32508619	795.5991735	NEG
MGDG(37:4)	MGDG	M-H	C46 H79 O10	9.454677092	791.5678735	NEG
MGDG(37:5)	MGDG	M+CH3COO	C48 H81 O12	8.100266134	849.5733535	POS
MGDG(37:6)	MGDG	M+CH3COO	C48 H79 O12	6.916787288	847.5577035	POS
MGDG(38:2e)	MGDG	M-H	C47 H87 O9	17.072	795.6355585	NEG
MGDG(38:3)	MGDG	M-H	C47 H83 O10	11.05	807.5991735	NEG

MGDG(38:3e)	MGDG	M+CH3COO	C49 H89 O11	16.35491567	853.6410385	POS
MGDG(38:4)	MGDG	M-H	C47 H81 O10	11.53144599	805.5835235	NEG
MGDG(38:4e)	MGDG	M-H	C47 H83 O9	15.673	791.6042585	NEG
MGDG(38:5)	MGDG	M-H	C47 H79 O10	10.23818243	803.5678735	NEG
MGDG(38:5e)	MGDG	M-H	C47 H81 O9	17.778	789.5886085	NEG
MGDG(38:6)	MGDG	M+CH3COO	C49 H81 O12	8.619	861.5733535	POS
MGDG(39:1)	MGDG	M-H	C48 H89 O10	14.445	825.6461235	NEG
MGDG(39:4)	MGDG	M-H	C48 H83 O10	14.129	819.5991735	NEG
MGDG(39:5)	MGDG	M-H	C48 H81 O10	9.586418252	817.5835235	NEG
MGDG(39:9)	MGDG	M-H	C48 H73 O10	13.302	809.5209235	NEG
MGDG(40:2e)	MGDG	M-H	C49 H91 O9	17.976	823.6668585	NEG
MGDG(40:3)	MGDG	M-H	C49 H87 O10	16.274	835.6304735	NEG
MGDG(40:5)	MGDG	M-H	C49 H83 O10	11.753	831.5991735	NEG
MGDG(40:6)	MGDG	M-H	C49 H81 O10	10.153	829.5835235	NEG
MGDG(40:7)	MGDG	M-H	C49 H79 O10	12.411	827.5678735	NEG
MGDG(41:10)	MGDG	M-H	C50 H75 O10	14.471	835.5365735	NEG
MGDG(41:3)	MGDG	M-H	C50 H89 O10	16.345	849.6461235	NEG
MGDG(41:4)	MGDG	M-H	C50 H87 O10	16.271	847.6304735	NEG
MGDG(42:2)	MGDG	M-H	C51 H93 O10	18.104	865.6774235	NEG
MGDG(42:3)	MGDG	M-H	C51 H91 O10	19.59848225	863.6617735	NEG
MGDG(42:5)	MGDG	M-H	C51 H87 O10	15.909	859.6304735	NEG

MGDG(42:6)	MGDG	M-H	C51 H85 O10	16.964077 61	857.61482 35	NEG
MGDG(42:6e)	MGDG	M-H	C51 H87 O9	5.5111169 18	843.63555 85	NEG
MGDG(43:11)	MGDG	M-H	C52 H77 O10	13.452	861.55222 35	NEG
MGDG(43:2)	MGDG	M+CH3C OO	C54 H99 O12	16.88	939.71420 35	POS
MGDG(43:3)	MGDG	M+CH3C OO	C54 H97 O12	17.071	937.69855 35	POS
MGDG(43:4)	MGDG	M+HCOO	C53 H93 O12	16.343	921.66725 35	POS
MGDG(44:2)	MGDG	M+HCOO	C54 H99 O12	17.937	939.71420 35	POS
MGDG(44:3)	MGDG	M+HCOO	C54 H97 O12	17.071	937.69855 35	POS
MGDG(44:4)	MGDG	M+CH3C OO	C55 H97 O12	13.130459 22	949.69855 35	POS
MGDG(44:5)	MGDG	M+CH3C OO	C55 H95 O12	12.345264 89	947.68290 35	POS
MGDG(45:2)	MGDG	M+CH3C OO	C56 H103 O12	18.35	967.74550 35	POS
MGDG(45:3)	MGDG	M+CH3C OO	C56 H101 O12	17.934	965.72985 35	POS
MGDG(45:4)	MGDG	M+CH3C OO	C56 H99 O12	15.979	963.71420 35	POS
MGDG(45:5)	MGDG	M+CH3C OO	C56 H97 O12	16.049553 97	961.69855 35	POS
MGDG(45:6)	MGDG	M-H	C54 H91 O10	16.099	899.66177 35	NEG
MGDG(45:7)	MGDG	M-H	C54 H89 O10	16.16	897.64612 35	NEG
MGDG(46:2)	MGDG	M+HCOO	C56 H103 O12	17.186	967.74550 35	POS
MGDG(46:3)	MGDG	M+HCOO	C56 H101 O12	21.445	965.72985 35	POS
MGDG(46:4)	MGDG	M-H	C55 H97 O10	16.854	917.70872 35	NEG
MGDG(46:5)	MGDG	M+HCOO	C56 H97 O12	16.481	961.69855 35	POS
MGDG(46:6)	MGDG	M+CH3C OO	C57 H97 O12	12.184189 02	973.69855 35	POS

MGDG(46:7)	MGDG	M-H	C55 H91 O10	12.739	911.6617735	NEG
MGDG(46:8)	MGDG	M-H	C55 H89 O10	16.98	909.6461235	NEG
MGDG(47:1)	MGDG	M-H	C56 H105 O10	20.422	937.7713235	NEG
MGDG(47:2)	MGDG	M-H	C56 H103 O10	19.799	935.7556735	NEG
MGDG(47:3)	MGDG	M+CH3COO	C58 H105 O12	18.632	993.7611535	POS
MGDG(47:4)	MGDG	M-H	C56 H99 O10	18.08012494	931.7243735	NEG
MGDG(47:5)	MGDG	M+CH3COO	C58 H101 O12	17.41331091	989.7298535	POS
MGDG(47:6)	MGDG	M+CH3COO	C58 H99 O12	16.834	987.7142035	POS
MGDG(47:7)	MGDG	M+CH3COO	C58 H97 O12	14.695	985.6985535	POS
MGDG(47:8)	MGDG	M+CH3COO	C58 H95 O12	12.911	983.6829035	POS
MGDG(48:3)	MGDG	M+CH3COO	C59 H107 O12	16.6113736	1007.776804	POS
MGDG(48:4)	MGDG	M-H	C57 H101 O10	18.09382377	945.7400235	NEG
MGDG(48:5)	MGDG	M+HCOO	C58 H101 O12	18.188	989.7298535	POS
MGDG(48:6)	MGDG	M+HCOO	C58 H99 O12	16.839	987.7142035	POS
MGDG(48:7)	MGDG	M+HCOO	C58 H97 O12	16.115	985.6985535	POS
MGDG(48:8)	MGDG	M-H	C57 H93 O10	12.197	937.6774235	NEG
MGDG(48:9)	MGDG	M+CH3COO	C59 H95 O12	16.712	995.6829035	POS
MGDG(49:2)	MGDG	M-H	C58 H107 O10	20.221	963.7869735	NEG
MGDG(49:3)	MGDG	M-H	C58 H105 O10	19.903	961.7713235	NEG
MGDG(49:4)	MGDG	M+CH3COO	C60 H107 O12	17.0428841	1019.776804	POS
MGDG(49:5)	MGDG	M+CH3COO	C60 H105 O12	16.04774001	1017.761154	POS

MGDG(50:10)	MGDG	M-H	C59 H93 O10	5.115565474	961.6774235	NEG
MGDG(50:2)	MGDG	M+HCOO	C60 H111 O12	19.866	1023.808104	POS
MGDG(50:3)	MGDG	M+HCOO	C60 H109 O12	17.58407335	1021.792454	POS
MGDG(51:3)	MGDG	M+CH3COO	C62 H113 O12	18.37243039	1049.823754	POS
MGDG(52:2)	MGDG	M+HCOO	C62 H115 O12	19.906	1051.839404	POS
MGDG(52:4)	MGDG	M+HCOO	C62 H111 O12	17.90232956	1047.808104	POS
MGDG(54:2)	MGDG	M+HCOO	C64 H119 O12	19.29519936	1079.870704	POS
MGDG(54:3)	MGDG	M+HCOO	C64 H117 O12	18.9703044	1077.855054	POS
MGDG(54:4)	MGDG	M+HCOO	C64 H115 O12	18.54989547	1075.839404	POS
MGMG(16:0)	MGMG	M+HCOO	C26 H49 O11	4.362067668	537.3280385	POS
MGMG(18:1)	MGMG	M+HCOO	C28 H51 O11	4.67088761	563.3436885	POS
MGMG(18:2)	MGMG	M+HCOO	C28 H49 O11	3.332553775	561.3280385	POS
MGMG(8:1)	MGMG	M+HCOO	C18 H31 O11	1.695017079	423.1871885	POS
MLCL(51:3)	MLCL	M-H	C60 H111 O16 P2	8.044	1149.73529	NEG
MLCL(54:5)	MLCL	M-H	C63 H113 O16 P2	16.84976677	1187.75094	NEG
MLCL(54:6)	MLCL	M-H	C63 H111 O16 P2	15.95835202	1185.73529	NEG
MLCL(55:2)	MLCL	M-H	C64 H121 O16 P2	11.77483882	1207.81354	NEG
MLCL(63:1)	MLCL	M-H	C72 H139 O16 P2	14.15224415	1321.95439	NEG
MLCL(63:3)	MLCL	M-H	C72 H135 O16 P2	13.02986191	1317.92309	NEG
MLCL(65:2)	MLCL	M-H	C74 H141 O16 P2	14.18672214	1347.97004	NEG
MLCL(65:4)	MLCL	M-H	C74 H137 O16 P2	13.08985339	1343.93874	NEG

MLCL(67:3)	MLCL	M-H	C76 H143 O16 P2	14.25986006	1373.98569	NEG
MLCL(67:4)	MLCL	M-H	C76 H141 O16 P2	14.194	1371.97004	NEG
MLCL(67:5)	MLCL	M-H	C76 H139 O16 P2	13.09311469	1369.95439	NEG
MLCL(69:4)	MLCL	M-H	C78 H145 O16 P2	14.26403036	1400.00134	NEG
MLCL(69:5)	MLCL	M-H	C78 H143 O16 P2	14.2	1397.98569	NEG
MLCL(69:6)	MLCL	M-H	C78 H141 O16 P2	13.16242635	1395.97004	NEG
MLCL(69:7)	MLCL	M-H	C78 H139 O16 P2	13.062	1393.95439	NEG
MLCL(69:8)	MLCL	M-H	C78 H137 O16 P2	13.095	1391.93874	NEG
MePC(27:3e)	MePC	M+Na	C36 H68 O7 N1 P1 Na1	4.347	680.4625635	POS
MePC(29:0)	MePC	M+Na	C38 H76 O8 N1 P1 Na1	10.94289446	728.5200785	POS
MePC(31:0)	MePC	M+Na	C40 H80 O8 N1 P1 Na1	9.311	756.5513785	POS
MePC(31:8e)	MePC	M+NH4	C40 H70 O7 N2 P1	5.359082919	721.4915175	POS
MePC(33:0)	MePC	M+Na	C42 H84 O8 N1 P1 Na1	13.51830186	784.5826785	POS
MePC(33:2)	MePC	M+Na	C42 H80 O8 N1 P1 Na1	11.33473344	780.5513785	POS
MePC(35:0)	MePC	M+Na	C44 H88 O8 N1 P1 Na1	14.815	812.6139785	POS
MePC(36:3e)	MePC	M+Na	C45 H86 O7 N1 P1 Na1	11.119	806.6034135	POS
MePC(36:4e)	MePC	M+Na	C45 H84 O7 N1 P1 Na1	9.723	804.5877635	POS
MePC(37:3)	MePC	M+Na	C46 H86 O8 N1 P1 Na1	7.401	834.5983285	POS
MePC(37:4)	MePC	M+Na	C46 H84 O8 N1 P1 Na1	6.257	832.5826785	POS
MePC(37:5)	MePC	M+Na	C46 H82 O8 N1 P1 Na1	7.238	830.5670285	POS
MePC(38:8)	MePC	M+NH4	C47 H82 O8 N2 P1	7.703	833.5803325	POS

MePC(38:9)	MePC	M+NH4	C47 H80 O8 N2 P1	7.641	831.5646825	POS
MePC(39:4e)	MePC	M+Na	C48 H90 O7 N1 P1 Na1	11.037	846.6347135	POS
OAHFA(16:0/12:0)	OAHFA	M-H	C28 H53 O4	14.10696106	453.3949335	NEG
OAHFA(16:0/14:0)	OAHFA	M-H	C30 H57 O4	15.55501592	481.4262335	NEG
OAHFA(16:0/15:0)	OAHFA	M-H	C31 H59 O4	16.164	495.4418835	NEG
OAHFA(16:0/16:0)	OAHFA	M-H	C32 H61 O4	14.52375155	509.4575335	NEG
OAHFA(16:0/18:0)	OAHFA	M-H	C34 H65 O4	15.73633927	537.4888335	NEG
OAHFA(16:0/18:1)	OAHFA	M-H	C34 H63 O4	14.84602868	535.4731835	NEG
OAHFA(16:0/18:2)	OAHFA	M-H	C34 H61 O4	14.48308977	533.4575335	NEG
OAHFA(16:0/19:0)	OAHFA	M-H	C35 H67 O4	18.009	551.5044835	NEG
OAHFA(16:0/20:0)	OAHFA	M-H	C36 H69 O4	18.77782014	565.5201335	NEG
OAHFA(16:0/21:0)	OAHFA	M-H	C37 H71 O4	19.1925237	579.5357835	NEG
OAHFA(16:0/22:0)	OAHFA	M-H	C38 H73 O4	19.45143197	593.5514335	NEG
OAHFA(16:0/23:0)	OAHFA	M-H	C39 H75 O4	19.77562488	607.5670835	NEG
OAHFA(16:0/24:0)	OAHFA	M-H	C40 H77 O4	18.73948111	621.5827335	NEG
OAHFA(16:0/25:0)	OAHFA	M-H	C41 H79 O4	20.29911118	635.5983835	NEG
OAHFA(16:0/26:0)	OAHFA	M-H	C42 H81 O4	20.51539291	649.6140335	NEG
OAHFA(16:0/27:0)	OAHFA	M-H	C43 H83 O4	20.74503692	663.6296835	NEG
OAHFA(16:0/28:0)	OAHFA	M-H	C44 H85 O4	20.954	677.6453335	NEG
OAHFA(16:0/29:0)	OAHFA	M-H	C45 H87 O4	21.139	691.6609835	NEG
OAHFA(16:0/30:0)	OAHFA	M-H	C46 H89 O4	21.355	705.6766335	NEG

OAHFA(16:0/31:0)	OAHFA	M-H	C47 H91 O4	21.48533108	719.6922835	NEG
OAHFA(18:0/18:1)	OAHFA	M-H	C36 H67 O4	16.20723511	563.5044835	NEG
OAHFA(18:0/25:0)	OAHFA	M-H	C43 H83 O4	19.718	663.6296835	NEG
OAHFA(18:0/26:0)	OAHFA	M-H	C44 H85 O4	19.971591	677.6453335	NEG
OAHFA(18:1/12:0)	OAHFA	M-H	C30 H55 O4	14.12845501	479.4105835	NEG
OAHFA(18:1/14:0)	OAHFA	M-H	C32 H59 O4	15.57795029	507.4418835	NEG
OAHFA(18:1/15:0)	OAHFA	M-H	C33 H61 O4	16.26144208	521.4575335	NEG
OAHFA(18:1/16:0)	OAHFA	M-H	C34 H63 O4	16.88937902	535.4731835	NEG
OAHFA(18:1/18:0)	OAHFA	M-H	C36 H67 O4	15.777	563.5044835	NEG
OAHFA(18:1/18:1)	OAHFA	M-H	C36 H65 O4	14.8572471	561.4888335	NEG
OAHFA(18:1/18:2)	OAHFA	M-H	C36 H63 O4	14.7861474	559.4731835	NEG
OAHFA(18:1/19:0)	OAHFA	M-H	C37 H69 O4	18.40489513	577.5201335	NEG
OAHFA(18:1/20:0)	OAHFA	M-H	C38 H71 O4	18.7771635	591.5357835	NEG
OAHFA(18:1/21:0)	OAHFA	M-H	C39 H73 O4	19.1333927	605.5514335	NEG
OAHFA(18:1/22:0)	OAHFA	M-H	C40 H75 O4	19.42417492	619.5670835	NEG
OAHFA(18:1/23:0)	OAHFA	M-H	C41 H77 O4	19.75725851	633.5827335	NEG
OAHFA(18:1/24:0)	OAHFA	M-H	C42 H79 O4	18.72600454	647.5983835	NEG
OAHFA(18:1/25:0)	OAHFA	M-H	C43 H81 O4	20.27837372	661.6140335	NEG
OAHFA(18:1/26:0)	OAHFA	M-H	C44 H83 O4	19.36688133	675.6296835	NEG
OAHFA(18:1/26:1)	OAHFA	M-H	C44 H81 O4	19.93935504	673.6140335	NEG
OAHFA(18:1/27:0)	OAHFA	M-H	C45 H85 O4	20.707	689.6453335	NEG

OAHFA(18:1/28:0)	OAHFA	M-H	C46 H87 O4	19.94987261	703.6609835	NEG
OAHFA(18:1/28:2)	OAHFA	M-H	C46 H83 O4	18.731	699.6296835	NEG
OAHFA(18:1/29:0)	OAHFA	M-H	C47 H89 O4	21.126	717.6766335	NEG
OAHFA(18:1/29:1)	OAHFA	M-H	C47 H87 O4	19.74630707	715.6609835	NEG
OAHFA(18:1/30:0)	OAHFA	M-H	C48 H91 O4	20.42444478	731.6922835	NEG
OAHFA(18:1/30:2)	OAHFA	M-H	C48 H87 O4	19.32279849	727.6609835	NEG
OAHFA(18:1/31:0)	OAHFA	M-H	C49 H93 O4	21.479	745.7079335	NEG
OAHFA(18:1/32:0)	OAHFA	M-H	C50 H95 O4	20.80241362	759.7235835	NEG
OAHFA(18:2/14:0)	OAHFA	M-H	C32 H57 O4	14.39553018	505.4262335	NEG
OAHFA(18:2/15:0)	OAHFA	M-H	C33 H59 O4	15.202	519.4418835	NEG
OAHFA(18:2/16:0)	OAHFA	M-H	C34 H61 O4	13.37564422	533.4575335	NEG
OAHFA(18:2/18:1)	OAHFA	M-H	C36 H63 O4	13.71259783	559.4731835	NEG
OAHFA(18:2/18:2)	OAHFA	M-H	C36 H61 O4	13.29285411	557.4575335	NEG
OAHFA(18:2/20:0)	OAHFA	M-H	C38 H69 O4	18.071625	589.5201335	NEG
OAHFA(18:2/21:0)	OAHFA	M-H	C39 H71 O4	18.5083667	603.5357835	NEG
OAHFA(18:2/22:0)	OAHFA	M-H	C40 H73 O4	18.86969672	617.5514335	NEG
OAHFA(18:2/23:0)	OAHFA	M-H	C41 H75 O4	19.22235573	631.5670835	NEG
OAHFA(18:2/24:0)	OAHFA	M-H	C42 H77 O4	18.07072521	645.5827335	NEG
OAHFA(18:2/24:1)	OAHFA	M-H	C42 H75 O4	18.78906737	643.5670835	NEG
OAHFA(18:2/25:0)	OAHFA	M-H	C43 H79 O4	18.46822581	659.5983835	NEG
OAHFA(18:2/26:0)	OAHFA	M-H	C44 H81 O4	18.83273664	673.6140335	NEG

OAHFA(18:2/26:1)	OAHFA	M-H	C44 H79 O4	19.434680 99	671.59838 35	NEG
OAHFA(18:2/27:0)	OAHFA	M-H	C45 H83 O4	19.179723	687.62968 35	NEG
OAHFA(18:2/28:0)	OAHFA	M-H	C46 H85 O4	19.383240 05	701.64533 35	NEG
OAHFA(18:2/28:2)	OAHFA	M-H	C46 H81 O4	18.074	697.61403 35	NEG
OAHFA(18:2/29:1)	OAHFA	M-H	C47 H85 O4	19.202	713.64533 35	NEG
OAHFA(18:2/30:0)	OAHFA	M-H	C48 H89 O4	19.983182 36	729.67663 35	NEG
OAHFA(18:2/32:0)	OAHFA	M-H	C50 H93 O4	20.403	757.70793 35	NEG
OAHFA(19:1/18:1)	OAHFA	M-H	C37 H67 O4	17.153	575.50448 35	NEG
OAHFA(20:0/18:1)	OAHFA	M-H	C38 H71 O4	17.395236 09	591.53578 35	NEG
OAHFA(20:0/26:0)	OAHFA	M-H	C46 H89 O4	20.416	705.67663 35	NEG
OAHFA(20:1/18:1)	OAHFA	M-H	C38 H69 O4	16.148603 78	589.52013 35	NEG
OAHFA(20:1/18:2)	OAHFA	M-H	C38 H67 O4	15.793126 57	587.50448 35	NEG
OAHFA(22:0/18:1)	OAHFA	M-H	C40 H75 O4	18.300226 19	619.56708 35	NEG
OAHFA(22:0/25:0)	OAHFA	M-H	C47 H91 O4	20.636229 5	719.69228 35	NEG
OAHFA(32:2)	OAHFA	M-H	C32 H57 O4	13.942	505.42623 35	NEG
OAHFA(34:0)	OAHFA	M-H	C34 H65 O4	17.690930 78	537.48883 35	NEG
OAHFA(35:0)	OAHFA	M-H	C35 H67 O4	17.269886 46	551.50448 35	NEG
OAHFA(36:1)	OAHFA	M-H	C36 H67 O4	7.5045625 88	563.50448 35	NEG
OAHFA(37:1)	OAHFA	M-H	C37 H69 O4	17.269432 07	577.52013 35	NEG
OAHFA(37:2)	OAHFA	M-H	C37 H67 O4	16.360019 26	575.50448 35	NEG
OAHFA(38:3)	OAHFA	M-H	C38 H67 O4	16.743635 61	587.50448 35	NEG

OAHFA(46:6)	OAHFA	M-H	C46 H77 O4	13.096	693.5827335	NEG
OAHFA(47:7)	OAHFA	M-H	C47 H77 O4	14.48947779	705.5827335	NEG
OAHFA(47:8)	OAHFA	M-H	C47 H75 O4	14.088	703.5670835	NEG
OAHFA(48:5)	OAHFA	M-H	C48 H83 O4	19.539	723.6296835	NEG
OAHFA(52:1)	OAHFA	M-H	C52 H99 O4	21.138	787.7548835	NEG
PA(10:0e/14:0)	PA	M-H	C27 H54 O7 N0 P1	11.07084455	521.3612665	NEG
PA(10:0e/6:0)	PA	M-H	C19 H38 O7 N0 P1	4.545953124	409.2360665	NEG
PA(12:1e/18:1)	PA	M-H	C33 H62 O7 N0 P1	11.131	601.4238665	NEG
PA(14:0/18:2)	PA	M-H	C35 H64 O8 N0 P1	11.66147609	643.4344315	NEG
PA(14:1e/23:0)	PA	M-H	C40 H78 O7 N0 P1	15.277	701.5490665	NEG
PA(15:0/18:1)	PA	M-H	C36 H68 O8 N0 P1	11.16	659.4657315	NEG
PA(15:0/18:2)	PA	M-H	C36 H66 O8 N0 P1	12.44570173	657.4500815	NEG
PA(15:1)	PA	M-H	C18 H32 O8 N0 P1	1.016094739	407.1840315	NEG
PA(15:2)	PA	M-H	C18 H30 O8 N0 P1	0.978276517	405.1683815	NEG
PA(16:0/14:0)	PA	M-H	C33 H64 O8 N0 P1	12.76499004	619.4344315	NEG
PA(16:0/14:1)	PA	M-H	C33 H62 O8 N0 P1	11.86	617.4187815	NEG
PA(16:0/16:0)	PA	M-H	C35 H68 O8 N0 P1	14.47403564	647.4657315	NEG
PA(16:0/18:1)	PA	M-H	C37 H70 O8 N0 P1	14.54865872	673.4813815	NEG
PA(16:0/18:2)	PA	M+NH4	C37 H73 O8 N1 P1	13.50877121	690.5068335	POS
PA(16:0/18:3)	PA	M-H	C37 H66 O8 N0 P1	12.08011269	669.4500815	NEG
PA(16:0/19:0)	PA	M-H	C38 H74 O8 N0 P1	13.826	689.5126815	NEG

PA(16:0/8:0)	PA	M-H	C27 H52 O8 N0 P1	7.197	535.3405315	NEG
PA(16:2e/19:0)	PA	M-H	C38 H72 O7 N0 P1	11.844	671.5021165	NEG
PA(17:0/18:1)	PA	M-H	C38 H72 O8 N0 P1	15.02101511	687.4970315	NEG
PA(17:1/16:0)	PA	M-H	C36 H68 O8 N0 P1	13.76793683	659.4657315	NEG
PA(17:1/18:1)	PA	M-H	C38 H70 O8 N0 P1	13.82756776	685.4813815	NEG
PA(18:0/11:1)	PA	M-H	C32 H60 O8 N0 P1	7.507339107	603.4031315	NEG
PA(18:0/18:1)	PA	M-H	C39 H74 O8 N0 P1	15.57316638	701.5126815	NEG
PA(18:0/18:2)	PA	M-H	C39 H72 O8 N0 P1	17.114	699.4970315	NEG
PA(18:1/12:0)	PA	M-H	C33 H62 O8 N0 P1	7.544323569	617.4187815	NEG
PA(18:1/12:1)	PA	M-H	C33 H60 O8 N0 P1	7.389	615.4031315	NEG
PA(18:1/14:0)	PA	M-H	C35 H66 O8 N0 P1	13.291	645.4500815	NEG
PA(18:1/18:1)	PA	M+NH4	C39 H77 O8 N1 P1	26.456	718.5381335	POS
PA(18:1/18:2)	PA	M+NH4	C39 H75 O8 N1 P1	13.81510906	716.5224835	POS
PA(18:1/22:0)	PA	M-H	C43 H82 O8 N0 P1	17.88720939	757.5752815	NEG
PA(18:1/23:0)	PA	M-H	C44 H84 O8 N0 P1	18.29057178	771.5909315	NEG
PA(18:1/24:0)	PA	M-H	C45 H86 O8 N0 P1	18.62321514	785.6065815	NEG
PA(18:2/14:1)	PA	M-H	C35 H62 O8 N0 P1	11.082	641.4187815	NEG
PA(18:2/18:2)	PA	M+NH4	C39 H73 O8 N1 P1	12.69	714.5068335	POS
PA(18:2/23:0)	PA	M-H	C44 H82 O8 N0 P1	17.58823	769.5752815	NEG
PA(18:2/23:1)	PA	M-H	C44 H80 O8 N0 P1	15.523	767.5596315	NEG
PA(18:2e/24:2)	PA	M-H	C45 H82 O7 N0 P1	13.39	765.5803665	NEG

PA(18:3/18:2)	PA	M-H	C39 H66 O8 N0 P1	11.358	693.4500815	NEG
PA(18:3e/18:3)	PA	M-H	C39 H66 O7 N0 P1	7.506	677.4551665	NEG
PA(19:0/22:6)	PA	M-H	C44 H74 O8 N0 P1	5.571	761.5126815	NEG
PA(19:1/16:0)	PA	M-H	C38 H72 O8 N0 P1	12.763	687.4970315	NEG
PA(19:1/18:1)	PA	M-H	C40 H74 O8 N0 P1	10.078	713.5126815	NEG
PA(19:1/22:6)	PA	M-H	C44 H72 O8 N0 P1	4.555	759.4970315	NEG
PA(20:0/18:1)	PA	M-H	C41 H78 O8 N0 P1	16.90919064	729.5439815	NEG
PA(20:0/18:2)	PA	M+NH4	C41 H81 O8 N1 P1	15.87385472	746.5694335	POS
PA(20:1/18:2)	PA	M-H	C41 H74 O8 N0 P1	14.47684766	725.5126815	NEG
PA(20:4e/17:0)	PA	M-H	C40 H72 O7 N0 P1	10.764577	695.5021165	NEG
PA(22:0/18:2)	PA	M-H	C43 H80 O8 N0 P1	17.08206779	755.5596315	NEG
PA(24:0/18:2)	PA	M-H	C45 H84 O8 N0 P1	18.58	783.5909315	NEG
PA(24:0/18:3)	PA	M-H	C45 H82 O8 N0 P1	17.375	781.5752815	NEG
PA(24:0/22:6)	PA	M-H	C49 H84 O8 N0 P1	12.175	831.5909315	NEG
PA(25:0/18:1)	PA	M-H	C46 H88 O8 N0 P1	19.05655738	799.6222315	NEG
PA(25:0/18:2)	PA	M-H	C46 H86 O8 N0 P1	18.41015701	797.6065815	NEG
PA(26:0/18:1)	PA	M-H	C47 H90 O8 N0 P1	19.432	813.6378815	NEG
PA(26:4e)	PA	M-H	C29 H50 O7 N0 P1	4.546	541.3299665	NEG
PA(34:1)	PA	M+Na	C37 H71 O8 N0 P1 Na1	14.20888509	697.4778795	POS
PA(34:2)	PA	M+Na	C37 H69 O8 N0 P1 Na1	13.08212851	695.4622295	POS
PA(36:3)	PA	M+Na	C39 H71 O8 N0 P1 Na1	13.17053775	721.4778795	POS

PA(36:4)	PA	M-H	C39 H68 O8 N0 P1	9.208478796	695.4657315	NEG
PA(36:5)	PA	M-H	C39 H66 O8 N0 P1	7.998	693.4500815	NEG
PA(42:2)	PA	M+NH4	C45 H89 O8 N1 P1	26.363	802.6320335	POS
PA(4:0/14:1)	PA	M-H	C21 H38 O8 N0 P1	1.606	449.2309815	NEG
PA(51:2)	PA	M-H	C54 H102 O8 N0 P1	17.604	909.7317815	NEG
PA(53:3)	PA	M-H	C56 H104 O8 N0 P1	19.81398984	935.7474315	NEG
PC(12:0e/6:0)	PC	M+H	C26 H55 O7 N1 P1	5.451278473	524.3710685	POS
PC(14:0/18:2)	PC	M+HCOO	C41 H77 O10 N1 P1	11.98667908	774.5290605	POS
PC(14:1e)	PC	M+HCOO	C23 H45 O9 N1 P1	1.042366733	510.2837455	POS
PC(15:0)	PC	M+H	C23 H47 O8 N1 P1	1.030476783	496.3033835	POS
PC(15:0/18:1)	PC	M+HCOO	C42 H81 O10 N1 P1	13.9339424	790.5603605	POS
PC(15:1)	PC	M+H	C23 H45 O8 N1 P1	1.01763414	494.2877335	POS
PC(16:0/14:0)	PC	M+HCOO	C39 H77 O10 N1 P1	13.08490197	750.5290605	POS
PC(16:0/16:0)	PC	M+HCOO	C41 H81 O10 N1 P1	14.53241534	778.5603605	POS
PC(16:0/16:1)	PC	M+HCOO	C41 H79 O10 N1 P1	10.24986376	776.5447105	POS
PC(16:0/18:1)	PC	M+HCOO	C43 H83 O10 N1 P1	14.59262126	804.5760105	POS
PC(16:0/18:2)	PC	M+HCOO	C43 H81 O10 N1 P1	13.46378824	802.5603605	POS
PC(16:0/18:3)	PC	M+HCOO	C43 H79 O10 N1 P1	12.43728006	800.5447105	POS
PC(16:0/20:2)	PC	M+H	C44 H85 O8 N1 P1	10.443	786.6007335	POS
PC(16:0/22:3)	PC	M+H	C46 H87 O8 N1 P1	10.954	812.6163835	POS
PC(16:0/24:2)	PC	M+H	C48 H93 O8 N1 P1	17.33183818	842.6633335	POS

PC(16:1/18:1)	PC	M+H	C42 H81 O8 N1 P1	14.959	758.5694335	POS
PC(16:1/18:2)	PC	M+HCOO	C43 H79 O10 N1 P1	12.1344177	800.5447105	POS
PC(17:0/18:1)	PC	M+HCOO	C44 H85 O10 N1 P1	15.29645751	818.5916605	POS
PC(17:0/18:2)	PC	M+HCOO	C44 H83 O10 N1 P1	14.18405669	816.5760105	POS
PC(17:1/18:1)	PC	M+HCOO	C44 H83 O10 N1 P1	13.81438882	816.5760105	POS
PC(18:0/16:0)	PC	M+H	C42 H85 O8 N1 P1	15.89754265	762.6007335	POS
PC(18:0/18:1)	PC	M+HCOO	C45 H87 O10 N1 P1	15.92371949	832.6073105	POS
PC(18:0/18:2)	PC	M+HCOO	C45 H85 O10 N1 P1	14.9040812	830.5916605	POS
PC(18:0e/18:2)	PC	M+H	C44 H87 O7 N1 P1	8.551500694	772.6214685	POS
PC(18:1)	PC	M+H	C26 H51 O8 N1 P1	1.591	536.3346835	POS
PC(18:1/14:0)	PC	M+HCOO	C41 H79 O10 N1 P1	13.16035275	776.5447105	POS
PC(18:1/14:2)	PC	M+H	C40 H75 O8 N1 P1	9.588070926	728.5224835	POS
PC(18:1/18:1)	PC	M+HCOO	C45 H85 O10 N1 P1	14.64576355	830.5916605	POS
PC(18:1/18:2)	PC	M+HCOO	C45 H83 O10 N1 P1	13.51809151	828.5760105	POS
PC(18:1/22:0)	PC	M+HCOO	C49 H95 O10 N1 P1	18.07442475	888.6699105	POS
PC(18:1/24:0)	PC	M+HCOO	C51 H99 O10 N1 P1	18.80211218	916.7012105	POS
PC(18:2/13:0)	PC	M+H	C39 H75 O8 N1 P1	11.269	716.5224835	POS
PC(18:2/18:2)	PC	M+HCOO	C45 H81 O10 N1 P1	12.36219955	826.5603605	POS
PC(18:2/21:1)	PC	M+H	C47 H89 O8 N1 P1	9.665	826.6320335	POS
PC(18:3/18:2)	PC	M+HCOO	C45 H79 O10 N1 P1	11.33722621	824.5447105	POS
PC(18:4/18:2)	PC	M+H	C44 H77 O8 N1 P1	10.32631688	778.5381335	POS

PC(20:0/13:0)	PC	M+H	C41 H83 O8 N1 P1	16.878	748.58508<br>35	POS
PC(20:0/18:1)	PC	M+HCOO	C47 H91 O10 N1 P1	17.120204<br>1	860.63861<br>05	POS
PC(20:0/18:2)	PC	M+HCOO	C47 H89 O10 N1 P1	16.184640<br>46	858.62296<br>05	POS
PC(20:1/18:1)	PC	M+HCOO	C47 H89 O10 N1 P1	15.875769<br>45	858.62296<br>05	POS
PC(20:1/18:2)	PC	M+HCOO	C47 H87 O10 N1 P1	14.809281<br>66	856.60731<br>05	POS
PC(20:2/18:2)	PC	M+HCOO	C47 H85 O10 N1 P1	13.741867<br>07	854.59166<br>05	POS
PC(20:2e/16:0)	PC	M+H	C44 H87 O7 N1 P1	11.941	772.62146<br>85	POS
PC(20:2e/18:2)	PC	M+H	C46 H87 O7 N1 P1	10.192599<br>91	796.62146<br>85	POS
PC(22:0/11:2)	PC	M+H	C41 H79 O8 N1 P1	12.741960<br>92	744.55378<br>35	POS
PC(22:1)	PC	M+CH3C<br>OO	C32 H61 O10 N1 P1	5.711	650.40386<br>05	POS
PC(22:1/18:2)	PC	M+H	C48 H91 O8 N1 P1	9.729	840.64768<br>35	POS
PC(24:0)	PC	M+H	C32 H65 O8 N1 P1	8.5779370<br>97	622.44423<br>35	POS
PC(24:0/18:2)	PC	M+HCOO	C51 H97 O10 N1 P1	18.214439<br>24	914.68556<br>05	POS
PC(26:0/18:1)	PC	M+HCOO	C53 H103 O10 N1 P1	19.420300<br>89	944.73251<br>05	POS
PC(26:1e)	PC	M+Na	C34 H68 O7 N1 P1<br>Na1	6.96	656.46256<br>35	POS
PC(27:0/16:0)	PC	M+H	C51 H103 O8 N1 P1	15.921267<br>47	888.74158<br>35	POS
PC(27:3e)	PC	M+H	C35 H67 O7 N1 P1	5.896	644.46496<br>85	POS
PC(28:0)	PC	M+H	C36 H73 O8 N1 P1	11.562	678.50683<br>35	POS
PC(30:0)	PC	M+H	C38 H77 O8 N1 P1	13.084901<br>97	706.53813<br>35	POS
PC(30:1)	PC	M+H	C38 H75 O8 N1 P1	11.668523<br>84	704.52248<br>35	POS
PC(30:2)	PC	M+HCOO	C39 H73 O10 N1 P1	9.5747901<br>33	746.49776<br>05	POS

PC(31:1/6:0)	PC	M+H	C45 H89 O8 N1 P1	10.516	802.6320335	POS
PC(32:2)	PC	M+HCOO	C41 H77 O10 N1 P1	8.94	774.5290605	POS
PC(32:3)	PC	M+H	C40 H75 O8 N1 P1	10.9591513	728.5224835	POS
PC(32:4)	PC	M+HCOO	C41 H73 O10 N1 P1	6.803	770.4977605	POS
PC(32:5)	PC	M+HCOO	C41 H71 O10 N1 P1	5.840166758	768.4821105	POS
PC(32:7e)	PC	M+H	C40 H69 O7 N1 P1	3.608	706.4806185	POS
PC(33:3)	PC	M+H	C41 H77 O8 N1 P1	11.71351566	742.5381335	POS
PC(34:3)	PC	M+H	C42 H79 O8 N1 P1	12.43728006	756.5537835	POS
PC(34:3e)	PC	M+CH3COO	C44 H83 O9 N1 P1	14.49718523	800.5810955	POS
PC(34:4)	PC	M+H	C42 H77 O8 N1 P1	12.681	754.5381335	POS
PC(35:2)	PC	M+H	C43 H83 O8 N1 P1	14.18405669	772.5850835	POS
PC(35:4e)	PC	M+H	C43 H81 O7 N1 P1	11.601	754.5745185	POS
PC(36:2e)	PC	M+H	C44 H87 O7 N1 P1	15.293	772.6214685	POS
PC(36:3)	PC	M+H	C44 H83 O8 N1 P1	13.51809151	784.5850835	POS
PC(36:3e)	PC	M+HCOO	C45 H85 O9 N1 P1	14.898	814.5967455	POS
PC(36:4)	PC	M+H	C44 H81 O8 N1 P1	9.315126214	782.5694335	POS
PC(36:4e)	PC	M+CH3COO	C46 H85 O9 N1 P1	14.87	826.5967455	POS
PC(36:5)	PC	M+H	C44 H79 O8 N1 P1	8.34507367	780.5537835	POS
PC(36:6)	PC	M+HCOO	C45 H77 O10 N1 P1	12.61162641	822.5290605	POS
PC(36:6e)	PC	M+H	C44 H79 O7 N1 P1	8.657	764.5588685	POS
PC(37:3e)	PC	M+H	C45 H87 O7 N1 P1	14.855	784.6214685	POS

PC(38:0)	PC	M+Na	C46 H92 O8 N1 P1 Na1	15.046	840.6452785	POS
PC(38:1e)	PC	M+Na	C46 H92 O7 N1 P1 Na1	16.13	824.6503635	POS
PC(38:3)	PC	M+H	C46 H87 O8 N1 P1	14.80928166	812.6163835	POS
PC(38:3e)	PC	M+H	C46 H89 O7 N1 P1	14.931	798.6371185	POS
PC(38:4)	PC	M+CH3COO	C48 H87 O10 N1 P1	15.06565989	868.6073105	POS
PC(38:4e)	PC	M+CH3COO	C48 H89 O9 N1 P1	15.98076348	854.6280455	POS
PC(38:5)	PC	M+CH3COO	C48 H85 O10 N1 P1	14.28310062	866.5916605	POS
PC(38:5e)	PC	M+CH3COO	C48 H87 O9 N1 P1	14.90953119	852.6123955	POS
PC(38:6)	PC	M+CH3COO	C48 H83 O10 N1 P1	13.26196352	864.5760105	POS
PC(38:6e)	PC	M+CH3COO	C48 H85 O9 N1 P1	13.813	850.5967455	POS
PC(38:7)	PC	M+HCOO	C47 H79 O10 N1 P1	12.81458091	848.5447105	POS
PC(38:7e)	PC	M+H	C46 H81 O7 N1 P1	9.285	790.5745185	POS
PC(39:4e)	PC	M+H	C47 H89 O7 N1 P1	14.147	810.6371185	POS
PC(40:4)	PC	M+H	C48 H89 O8 N1 P1	10.988	838.6320335	POS
PC(40:5)	PC	M+CH3COO	C50 H89 O10 N1 P1	15.08710558	894.6229605	POS
PC(40:6)	PC	M+H	C48 H85 O8 N1 P1	7.599069291	834.6007335	POS
PC(40:6e)	PC	M+H	C48 H87 O7 N1 P1	11.6733861	820.6214685	POS
PC(40:7)	PC	M+H	C48 H83 O8 N1 P1	5.136	832.5850835	POS
PC(40:7e)	PC	M+H	C48 H85 O7 N1 P1	6.626	818.6058185	POS
PC(40:8e)	PC	M+H	C48 H83 O7 N1 P1	6.062	816.5901685	POS
PC(41:4e)	PC	M+H	C49 H93 O7 N1 P1	15.909	838.6684185	POS

PC(42:6e)	PC	M+Na	C50 H90 O7 N1 P1 Na1	7.036	870.6347135	POS
PC(42:7e)	PC	M+H	C50 H89 O7 N1 P1	11.109	846.6371185	POS
PC(43:1)	PC	M+CH3COO	C53 H103 O10 N1 P1	15.186	944.7325105	POS
PC(43:3e)	PC	M+H	C51 H99 O7 N1 P1	17.192	868.7153685	POS
PC(44:1)	PC	M+CH3COO	C54 H105 O10 N1 P1	18.43	958.7481605	POS
PC(45:0)	PC	M+HCOO	C54 H107 O10 N1 P1	17.555	960.7638105	POS
PC(45:1)	PC	M+Na	C53 H104 O8 N1 P1 Na1	12.912	936.7391785	POS
PC(45:2)	PC	M+Na	C53 H102 O8 N1 P1 Na1	17.632	934.7235285	POS
PC(47:0)	PC	M+Na	C55 H110 O8 N1 P1 Na1	17.727	966.7861285	POS
PC(47:1)	PC	M+Na	C55 H108 O8 N1 P1 Na1	20.53	964.7704785	POS
PC(47:2)	PC	M+Na	C55 H106 O8 N1 P1 Na1	17.387	962.7548285	POS
PC(47:3)	PC	M+Na	C55 H104 O8 N1 P1 Na1	13.586	960.7391785	POS
PC(47:4)	PC	M+Na	C55 H102 O8 N1 P1 Na1	9.748	958.7235285	POS
PC(49:3)	PC	M+H	C57 H109 O8 N1 P1	15.56730592	966.7885335	POS
PC(50:3)	PC	M+H	C58 H111 O8 N1 P1	16.895	980.8041835	POS
PC(51:1)	PC	M+Na	C59 H116 O8 N1 P1 Na1	18.909	1020.833078	POS
PC(51:5)	PC	M+H	C59 H109 O8 N1 P1	18.384	990.7885335	POS
PC(53:1)	PC	M+Na	C61 H120 O8 N1 P1 Na1	19.08	1048.864378	POS
PC(54:0)	PC	M+Na	C62 H124 O8 N1 P1 Na1	19.114	1064.895678	POS
PC(54:2)	PC	M+Na	C62 H120 O8 N1 P1 Na1	17.97	1060.864378	POS
PC(55:1)	PC	M+H	C63 H125 O8 N1 P1	20.645	1054.913733	POS

PC(55:2)	PC	M+Na	C63 H122 O8 N1 P1 Na1	18.248	1074.880028	POS
PC(56:2)	PC	M+Na	C64 H124 O8 N1 P1 Na1	19.448	1088.895678	POS
PC(59:7)	PC	M+H	C67 H121 O8 N1 P1	14.577	1098.882433	POS
PE(12:1e/6:0)	PE	M+Na	C23 H46 O7 N1 P1 Na1	4.019952987	502.2904135	POS
PE(14:0/18:2)	PE	M+H	C37 H71 O8 N1 P1	12.34524363	688.4911835	POS
PE(15:0/18:2)	PE	M+H	C38 H73 O8 N1 P1	13.09189425	702.5068335	POS
PE(16:0/12:3)	PE	M+H	C33 H61 O8 N1 P1	9.562	630.4129335	POS
PE(16:0/14:0)	PE	M-H	C35 H69 O8 N1 P1	13.47392767	662.4766305	NEG
PE(16:0/16:0)	PE	M-H	C37 H73 O8 N1 P1	12.503	690.5079305	NEG
PE(16:0/18:1)	PE	M-H	C39 H75 O8 N1 P1	14.94889021	716.5235805	NEG
PE(16:0/18:2)	PE	M+H	C39 H75 O8 N1 P1	13.82992876	716.5224835	POS
PE(16:0/18:3)	PE	M-H	C39 H71 O8 N1 P1	12.83347785	712.4922805	NEG
PE(16:1/18:2)	PE	M+H	C39 H73 O8 N1 P1	12.44986347	714.5068335	POS
PE(17:0/18:2)	PE	M-H	C40 H75 O8 N1 P1	14.53997839	728.5235805	NEG
PE(17:1/16:0)	PE	M-H	C38 H73 O8 N1 P1	14.47913541	702.5079305	NEG
PE(18:0/18:1)	PE	M-H	C41 H79 O8 N1 P1	16.26002913	744.5548805	NEG
PE(18:1/12:3)	PE	M+H	C35 H63 O8 N1 P1	9.666	656.4285835	POS
PE(18:1/14:0)	PE	M+H	C37 H73 O8 N1 P1	13.54181192	690.5068335	POS
PE(18:1/18:1)	PE	M-H	C41 H77 O8 N1 P1	14.99813825	742.5392305	NEG
PE(18:1/18:2)	PE	M+H	C41 H77 O8 N1 P1	13.87615615	742.5381335	POS
PE(18:1/22:0)	PE	M-H	C45 H87 O8 N1 P1	18.2668937	800.6174805	NEG

PE(18:1/24:0)	PE	M-H	C47 H91 O8 N1 P1	18.9644795	828.6487805	NEG
PE(18:2/18:2)	PE	M+H	C41 H75 O8 N1 P1	12.71289917	740.5224835	POS
PE(18:2/23:0)	PE	M-H	C46 H87 O8 N1 P1	17.997	812.6174805	NEG
PE(18:3/18:2)	PE	M-H	C41 H71 O8 N1 P1	11.68721743	736.4922805	NEG
PE(20:0/18:1)	PE	M-H	C43 H83 O8 N1 P1	17.40817095	772.5861805	NEG
PE(20:0/18:2)	PE	M-H	C43 H81 O8 N1 P1	16.50263324	770.5705305	NEG
PE(20:1/18:1)	PE	M-H	C43 H81 O8 N1 P1	16.21845948	770.5705305	NEG
PE(22:0/18:2)	PE	M-H	C45 H85 O8 N1 P1	17.56994305	798.6018305	NEG
PE(24:0/18:2)	PE	M-H	C47 H89 O8 N1 P1	18.39668946	826.6331305	NEG
PE(26:0/18:1)	PE	M-H	C49 H95 O8 N1 P1	19.56699343	856.6800805	NEG
PE(26:0/18:2)	PE	M-H	C49 H93 O8 N1 P1	19.06845502	854.6644305	NEG
PE(32:1)	PE	M+H	C37 H73 O8 N1 P1	25.5	690.5068335	POS
PE(34:0)	PE	M+Na	C39 H78 O8 N1 P1 Na1	10.645	742.5357285	POS
PE(34:2)	PE	M+Na	C39 H74 O8 N1 P1 Na1	11.67776539	738.5044285	POS
PE(35:1)	PE	M-H	C40 H77 O8 N1 P1	14.94065486	730.5392305	NEG
PE(35:2)	PE	M-H	C40 H75 O8 N1 P1	13.82182785	728.5235805	NEG
PE(36:2e)	PE	M-H	C41 H79 O7 N1 P1	16.084	728.5599655	NEG
PE(36:3)	PE	M-H	C41 H75 O8 N1 P1	14.171	740.5235805	NEG
PE(36:3e)	PE	M-H	C41 H77 O7 N1 P1	15.121	726.5443155	NEG
PE(36:4)	PE	M+H	C41 H75 O8 N1 P1	9.719741219	740.5224835	POS
PE(37:2)	PE	M-H	C42 H79 O8 N1 P1	14.98807909	756.5548805	NEG

PE(38:2e)	PE	M-H	C43 H83 O7 N1 P1	16.868	756.5912655	NEG
PE(48:0)	PE	M+Na	C53 H106 O8 N1 P1 Na1	23.062	938.7548285	POS
PE(50:1)	PE	M+Na	C55 H108 O8 N1 P1 Na1	24.01	964.7704785	POS
PE(50:2)	PE	M+Na	C55 H106 O8 N1 P1 Na1	23.282	962.7548285	POS
PE(52:7)	PE	M+H	C57 H101 O8 N1 P1	10.817	958.7259335	POS
PE(54:7)	PE	M+Na	C59 H104 O8 N1 P1 Na1	12.437	1008.739178	POS
PE(60:6)	PE	M+Na	C65 H118 O8 N1 P1 Na1	13.261	1094.848728	POS
PE(6:0/12:2)	PE	M+H	C23 H43 O8 N1 P1	1.370815764	492.2720835	POS
PEt(16:1/18:1)	PEt	M-H	C39 H72 O8 N0 P1	14.251	699.4970315	NEG
PEt(28:1e)	PEt	M-H	C33 H64 O7 N0 P1	9.184	603.4395165	NEG
PEt(30:2)	PEt	M-H	C35 H64 O8 N0 P1	8.338	643.4344315	NEG
PEt(30:2e)	PEt	M-H	C35 H66 O7 N0 P1	10.307	629.4551665	NEG
PEt(31:2)	PEt	M-H	C36 H66 O8 N0 P1	10.806	657.4500815	NEG
PEt(33:1)	PEt	M-H	C38 H72 O8 N0 P1	12.098	687.4970315	NEG
PEt(33:2)	PEt	M-H	C38 H70 O8 N0 P1	11.462	685.4813815	NEG
PEt(35:3)	PEt	M-H	C40 H72 O8 N0 P1	10.833	711.4970315	NEG
PEt(37:1)	PEt	M+H	C42 H82 O8 N0 P1	7.691600496	745.5741845	POS
PEt(38:4e)	PEt	M-H	C43 H78 O7 N0 P1	11.707	737.5490665	NEG
PEt(42:3)	PEt	M-H	C47 H86 O8 N0 P1	11.952	809.6065815	NEG
PG(14:0/18:2)	PG	M-H	C38 H70 O10 N0 P1	11.13128423	717.4712115	NEG
PG(15:0/16:0)	PG	M-H	C37 H72 O10 N0 P1	12.86000631	707.4868615	NEG

PG(16:0/14:0)	PG	M-H	C36 H70 O10 N0 P1	12.16280012	693.4712115	NEG
PG(16:0/16:0)	PG	M+Na	C38 H75 O10 N0 P1 Na1	13.59747873	745.4990095	POS
PG(16:0/18:1)	PG	M+Na	C40 H77 O10 N0 P1 Na1	13.65895855	771.5146595	POS
PG(16:0/18:2)	PG	M-H	C40 H74 O10 N0 P1	11.84353917	745.5025115	NEG
PG(16:0/18:3)	PG	M-H	C40 H72 O10 N0 P1	11.58309308	743.4868615	NEG
PG(18:1/14:0)	PG	M-H	C38 H72 O10 N0 P1	12.24379813	719.4868615	NEG
PG(18:1/18:1)	PG	M-H	C42 H78 O10 N0 P1	13.71984884	773.5338115	NEG
PG(18:1/18:2)	PG	M+NH4	C42 H81 O10 N1 P1	12.62505576	790.5592635	POS
PG(18:2/18:2)	PG	M-H	C42 H74 O10 N0 P1	11.52578831	769.5025115	NEG
PG(18:3/18:2)	PG	M-H	C42 H72 O10 N0 P1	10.57864527	767.4868615	NEG
PG(19:1/16:0)	PG	M-H	C41 H78 O10 N0 P1	14.525	761.5338115	NEG
PG(26:2)	PG	M-H	C32 H58 O10 N0 P1	5.399	633.3773115	NEG
PG(29:2)	PG	M-H	C35 H64 O10 N0 P1	7.641499271	675.4242615	NEG
PG(30:0)	PG	M-H	C36 H70 O10 N0 P1	3.701792931	693.4712115	NEG
PG(30:1)	PG	M-H	C36 H68 O10 N0 P1	10.218	691.4555615	NEG
PG(31:1)	PG	M-H	C37 H70 O10 N0 P1	10.08675672	705.4712115	NEG
PG(31:2)	PG	M-H	C37 H68 O10 N0 P1	7.476251768	703.4555615	NEG
PG(31:3)	PG	M-H	C37 H66 O10 N0 P1	11.097	701.4399115	NEG
PG(32:2e)	PG	M-H	C38 H72 O9 N0 P1	9.956	703.4919465	NEG
PG(32:3)	PG	M-H	C38 H68 O10 N0 P1	8.381	715.4555615	NEG
PG(32:4)	PG	M-H	C38 H66 O10 N0 P1	8.337	713.4399115	NEG

PG(32:5)	PG	M-H	C38 H64 O10 N0 P1	11.678	711.4242615	NEG
PG(33:0)	PG	M-H	C39 H76 O10 N0 P1	7.688	735.5181615	NEG
PG(33:1)	PG	M-H	C39 H74 O10 N0 P1	8.598	733.5025115	NEG
PG(33:2)	PG	M-H	C39 H72 O10 N0 P1	7.491450062	731.4868615	NEG
PG(33:3)	PG	M-H	C39 H70 O10 N0 P1	9.253943007	729.4712115	NEG
PG(33:4)	PG	M-H	C39 H68 O10 N0 P1	8.378	727.4555615	NEG
PG(34:4)	PG	M-H	C40 H70 O10 N0 P1	14.26806015	741.4712115	NEG
PG(35:2)	PG	M-H	C41 H76 O10 N0 P1	3.778	759.5181615	NEG
PG(35:3)	PG	M-H	C41 H74 O10 N0 P1	13.024	757.5025115	NEG
PG(36:1)	PG	M-H	C42 H80 O10 N0 P1	14.28976915	775.5494615	NEG
PG(36:1e)	PG	M-H	C42 H82 O9 N0 P1	9.24331703	761.5701965	NEG
PG(36:4)	PG	M-H	C42 H74 O10 N0 P1	15.58927611	769.5025115	NEG
PG(36:4e)	PG	M-H	C42 H76 O9 N0 P1	10.58	755.5232465	NEG
PG(36:5)	PG	M-H	C42 H72 O10 N0 P1	14.29663309	767.4868615	NEG
PG(36:5e)	PG	M-H	C42 H74 O9 N0 P1	9.233	753.5075965	NEG
PG(36:6)	PG	M-H	C42 H70 O10 N0 P1	13.2510632	765.4712115	NEG
PG(37:2)	PG	M-H	C43 H80 O10 N0 P1	6.15	787.5494615	NEG
PG(38:2)	PG	M-H	C44 H82 O10 N0 P1	14.43	801.5651115	NEG
PG(38:3)	PG	M-H	C44 H80 O10 N0 P1	13.157	799.5494615	NEG
PG(38:4)	PG	M-H	C44 H78 O10 N0 P1	12.089	797.5338115	NEG
PG(40:4)	PG	M-H	C46 H82 O10 N0 P1	14.43671734	825.5651115	NEG

PG(41:3)	PG	M-H	C47 H86 O10 N0 P1	17.391	841.5964115	NEG
PG(43:0)	PG	M+NH4	C49 H101 O10 N1 P1	18.379	894.7157635	POS
PG(44:0)	PG	M+H	C50 H100 O10 N0 P1	14.58111343	891.7048645	POS
PG(44:1)	PG	M+NH4	C50 H101 O10 N1 P1	15.82	906.7157635	POS
PG(46:0)	PG	M+H	C52 H104 O10 N0 P1	19.088	919.7361645	POS
PG(46:3)	PG	M-H	C52 H96 O10 N0 P1	17.082	911.6746615	NEG
PG(48:1)	PG	M+H	C54 H106 O10 N0 P1	15.32592422	945.7518145	POS
PG(48:2)	PG	M-H	C54 H102 O10 N0 P1	16.613	941.7216115	NEG
PG(49:1)	PG	M+Na	C55 H107 O10 N0 P1 Na1	17.382	981.7494095	POS
PG(50:1)	PG	M+Na	C56 H109 O10 N0 P1 Na1	15.394	995.7650595	POS
PG(50:2)	PG	M+NH4	C56 H111 O10 N1 P1	16.076	988.7940135	POS
PG(50:3)	PG	M+H	C56 H106 O10 N0 P1	16.282	969.7518145	POS
PG(52:3)	PG	M-H	C58 H108 O10 N0 P1	16.471	995.7685615	NEG
PG(53:0)	PG	M+NH4	C59 H121 O10 N1 P1	18.541	1034.872263	POS
PG(54:0)	PG	M+Na	C60 H119 O10 N0 P1 Na1	16.96	1053.843309	POS
PG(54:3)	PG	M+H	C60 H114 O10 N0 P1	12.694	1025.814414	POS
PG(55:1)	PG	M+NH4	C61 H123 O10 N1 P1	19.149	1060.887913	POS
PI(10:1e/18:1)	PI	M-H	C37 H68 O12 N0 P1	7.667	735.4453915	NEG
PI(12:0/18:2)	PI	M-H	C39 H70 O13 N0 P1	9.34	777.4559565	NEG
PI(12:0e/18:2)	PI	M-H	C39 H72 O12 N0 P1	6.187968313	763.4766915	NEG
PI(12:1e/18:1)	PI	M-H	C39 H72 O12 N0 P1	5.849530225	763.4766915	NEG

PI(12:1e/18:2)	PI	M-H	C39 H70 O12 N0 P1	7.644870689	761.4610415	NEG
PI(14:0/18:2)	PI	M-H	C41 H74 O13 N0 P1	10.81794504	805.4872565	NEG
PI(14:0/18:3)	PI	M-H	C41 H72 O13 N0 P1	9.853606253	803.4716065	NEG
PI(14:0/20:3)	PI	M-H	C43 H76 O13 N0 P1	6.398195679	831.5029065	NEG
PI(15:0)	PI	M-H	C24 H44 O13 N0 P1	0.963884694	571.2525065	NEG
PI(15:0/18:1)	PI	M-H	C42 H78 O13 N0 P1	12.68871596	821.5185565	NEG
PI(15:0/18:2)	PI	M-H	C42 H76 O13 N0 P1	11.57137789	819.5029065	NEG
PI(16:0/12:3)	PI	M+H	C37 H66 O13 N0 P1	8.257646164	749.4235595	POS
PI(16:0/14:0)	PI	M-H	C39 H74 O13 N0 P1	11.8585393	781.4872565	NEG
PI(16:0/16:0)	PI	M-H	C41 H78 O13 N0 P1	13.30202275	809.5185565	NEG
PI(16:0/18:1)	PI	M+Na	C43 H81 O13 N0 P1 Na1	13.37570608	859.5307045	POS
PI(16:0/18:2)	PI	M-H	C43 H78 O13 N0 P1	12.2594443	833.5185565	NEG
PI(16:0/18:3)	PI	M+H	C43 H78 O13 N0 P1	8.334110613	833.5174595	POS
PI(16:0/19:0)	PI	M-H	C44 H84 O13 N0 P1	14.96241645	851.5655065	NEG
PI(16:0/8:0)	PI	M-H	C33 H62 O13 N0 P1	7.400755645	697.3933565	NEG
PI(16:1/18:2)	PI	M-H	C43 H76 O13 N0 P1	10.96180093	831.5029065	NEG
PI(17:0/18:1)	PI	M-H	C44 H82 O13 N0 P1	14.0854962	849.5498565	NEG
PI(17:0/18:2)	PI	M-H	C44 H80 O13 N0 P1	12.98708739	847.5342065	NEG
PI(17:1/18:2)	PI	M-H	C44 H78 O13 N0 P1	11.7	845.5185565	NEG
PI(18:0/16:0)	PI	M-H	C43 H82 O13 N0 P1	14.6773194	837.5498565	NEG
PI(18:0/18:1)	PI	M-H	C45 H84 O13 N0 P1	14.73951714	863.5655065	NEG

PI(18:0/18:2)	PI	M+H	C45 H84 O13 N0 P1	13.445708 89	863.56440 95	POS
PI(18:1/12:3)	PI	M+H	C39 H68 O13 N0 P1	8.392	775.43920 95	POS
PI(18:1/14:0)	PI	M-H	C41 H76 O13 N0 P1	11.960427 78	807.50290 65	NEG
PI(18:1/18:2)	PI	M-H	C45 H80 O13 N0 P1	12.330943 27	859.53420 65	NEG
PI(18:1/24:0)	PI	M-H	C51 H96 O13 N0 P1	18.01	947.65940 65	NEG
PI(18:2/12:3)	PI	M+H	C39 H66 O13 N0 P1	7.301	773.42355 95	POS
PI(18:2/18:2)	PI	M-H	C45 H78 O13 N0 P1	11.224175 07	857.51855 65	NEG
PI(18:3/18:2)	PI	M+H	C45 H78 O13 N0 P1	7.284	857.51745 95	POS
PI(18:3/18:3)	PI	M-H	C45 H74 O13 N0 P1	9.278	853.48725 65	NEG
PI(18:4/20:0)	PI	M+H	C47 H84 O13 N0 P1	14.905	887.56440 95	POS
PI(20:0/18:1)	PI	M-H	C47 H88 O13 N0 P1	16.006	891.59680 65	NEG
PI(20:0e/16:0)	PI	M-H	C45 H88 O12 N0 P1	8.493	851.60189 15	NEG
PI(20:1/18:1)	PI	M-H	C47 H86 O13 N0 P1	14.725097 86	889.58115 65	NEG
PI(20:1/18:2)	PI	M-H	C47 H84 O13 N0 P1	13.648207 79	887.56550 65	NEG
PI(21:3)	PI	M-H	C30 H50 O13 N0 P1	0.9566454 25	649.29945 65	NEG
PI(21:4e)	PI	M-H	C30 H50 O12 N0 P1	1.056	633.30454 15	NEG
PI(24:0/18:2)	PI	M-H	C51 H94 O13 N0 P1	17.314845 64	945.64375 65	NEG
PI(28:1/18:1)	PI	M-H	C55 H102 O13 N0 P1	14.489358 13	1001.7063 57	NEG
PI(28:1/18:2)	PI	M-H	C55 H100 O13 N0 P1	13.691432 96	999.69070 65	NEG
PI(30:2)	PI	M-H	C39 H70 O13 N0 P1	6.641	777.45595 65	NEG
PI(34:1e)	PI	M-H	C43 H82 O12 N0 P1	17.811753 62	821.55494 15	NEG

PI(34:2e)	PI	M-H	C43 H80 O12 N0 P1	16.973	819.53929 15	NEG
PI(34:3e)	PI	M-H	C43 H78 O12 N0 P1	15.971	817.52364 15	NEG
PI(36:2)	PI	M+NH4	C45 H87 O13 N1 P1	13.445708 89	880.59095 85	POS
PI(36:3)	PI	M-H	C45 H80 O13 N0 P1	8.027	859.53420 65	NEG
PI(36:4e)	PI	M-H	C45 H80 O12 N0 P1	8.079	843.53929 15	NEG
PI(38:2)	PI	M+NH4	C47 H91 O13 N1 P1	8.509	908.62225 85	POS
PI(38:4)	PI	M-H	C47 H82 O13 N0 P1	8.191	885.54985 65	NEG
PI(38:5)	PI	M-H	C47 H80 O13 N0 P1	6.928	883.53420 65	NEG
PI(43:0)	PI	M+H	C52 H102 O13 N0 P1	14.134281 43	965.70525 95	POS
PI(47:4)	PI	M-H	C56 H100 O13 N0 P1	11.225	1011.6907 07	NEG
PI(48:3)	PI	M-H	C57 H104 O13 N0 P1	14.789	1027.7220 07	NEG
PI(48:4)	PI	M-H	C57 H102 O13 N0 P1	13.634	1025.7063 57	NEG
PIP(18:3e/16:0)	PIP	M-H	C43 H79 O15 N0 P2	6.741	897.48997 45	NEG
PIP(18:3e/18:1)	PIP	M-H	C45 H81 O15 N0 P2	5.9	923.50562 45	NEG
PIP(52:3)	PIP	M+Na	C61 H114 O16 N0 P2 Na1	15.895	1187.7474 37	POS
PMe(16:0/18:1)	PMe	M-H	C38 H72 O8 N0 P1	11	687.49703 15	NEG
PMe(16:0/18:2)	PMe	M-H	C38 H70 O8 N0 P1	11.394262 77	685.48138 15	NEG
PMe(17:1/18:1)	PMe	M-H	C39 H72 O8 N0 P1	17.757	699.49703 15	NEG
PMe(18:1/18:1)	PMe	M-H	C40 H74 O8 N0 P1	14.270084 69	713.51268 15	NEG
PMe(18:1/18:2)	PMe	M-H	C40 H72 O8 N0 P1	13.144984 11	711.49703 15	NEG
PMe(28:1)	PMe	M-H	C32 H60 O8 N0 P1	7.493	603.40313 15	NEG

PMe(32:2)	PMe	M-H	C36 H66 O8 N0 P1	7.994	657.4500815	NEG
PMe(32:2e)	PMe	M-H	C36 H68 O7 N0 P1	10.013	643.4708165	NEG
PMe(32:3)	PMe	M-H	C36 H64 O8 N0 P1	7.703	655.4344315	NEG
PMe(34:2)	PMe	M-H	C38 H70 O8 N0 P1	11.003	685.4813815	NEG
PMe(36:2e)	PMe	M-H	C40 H76 O7 N0 P1	11.51	699.5334165	NEG
PMe(36:3e)	PMe	M-H	C40 H74 O7 N0 P1	12.373	697.5177665	NEG
PMe(36:4e)	PMe	M-H	C40 H72 O7 N0 P1	10.428	695.5021165	NEG
PMe(38:3)	PMe	M-H	C42 H76 O8 N0 P1	15.246	739.5283315	NEG
PMe(39:3)	PMe	M-H	C43 H78 O8 N0 P1	15.193	753.5439815	NEG
PMe(40:7)	PMe	M-H	C44 H72 O8 N0 P1	6.006	759.4970315	NEG
PS(16:0/18:1)	PS	M-H	C40 H75 O10 N1 P1	13.62368475	760.5134105	NEG
PS(16:0/18:2)	PS	M-H	C40 H73 O10 N1 P1	12.46502921	758.4977605	NEG
PS(18:1/18:2)	PS	M-H	C42 H75 O10 N1 P1	8.825	784.5134105	NEG
PS(18:1/22:0)	PS	M-H	C46 H87 O10 N1 P1	17.38	844.6073105	NEG
PS(18:2/18:2)	PS	M-H	C42 H73 O10 N1 P1	13.8457577	782.4977605	NEG
PS(24:0/18:2)	PS	M-H	C48 H89 O10 N1 P1	17.48275015	870.6229605	NEG
PS(26:1e)	PS	M+H	C32 H63 O9 N1 P1	4.651	636.4234985	POS
PS(30:3)	PS	M-H	C36 H63 O10 N1 P1	3.135	700.4195105	NEG
PS(32:1e)	PS	M-H	C38 H73 O9 N1 P1	13.598	718.5028455	NEG
PS(32:2e)	PS	M-H	C38 H71 O9 N1 P1	12.245	716.4871955	NEG
PS(33:1)	PS	M+H	C39 H75 O10 N1 P1	9.361232563	748.5123135	POS

PS(34:2e)	PS	M-H	C40 H75 O9 N1 P1	13.65423456	744.5184955	NEG
PS(34:3e)	PS	M-H	C40 H73 O9 N1 P1	12.55061596	742.5028455	NEG
PS(35:2)	PS	M+H	C41 H77 O10 N1 P1	12.51	774.5279635	POS
PS(35:3)	PS	M+H	C41 H75 O10 N1 P1	7.376	772.5123135	POS
PS(36:2e)	PS	M-H	C42 H79 O9 N1 P1	14.994	772.5497955	NEG
PS(36:3e)	PS	M-H	C42 H77 O9 N1 P1	13.70897731	770.5341455	NEG
PS(36:4e)	PS	M-H	C42 H75 O9 N1 P1	12.61272157	768.5184955	NEG
PS(36:5e)	PS	M-H	C42 H73 O9 N1 P1	11.52770847	766.5028455	NEG
PS(38:4)	PS	M-H	C44 H77 O10 N1 P1	15.02242719	810.5290605	NEG
PS(38:4e)	PS	M-H	C44 H79 O9 N1 P1	13.4862648	796.5497955	NEG
PS(38:5)	PS	M-H	C44 H75 O10 N1 P1	13.91740576	808.5134105	NEG
PS(38:5e)	PS	M-H	C44 H77 O9 N1 P1	12.44227813	794.5341455	NEG
PS(38:6)	PS	M-H	C44 H73 O10 N1 P1	12.74880535	806.4977605	NEG
PS(38:6e)	PS	M-H	C44 H75 O9 N1 P1	11.35546712	792.5184955	NEG
PS(43:2)	PS	M+H	C49 H93 O10 N1 P1	8.85	886.6531635	POS
PS(44:2)	PS	M+H	C50 H95 O10 N1 P1	9.725	900.6688135	POS
PS(47:0)	PS	M+Na	C53 H104 O10 N1 P1 Na1	16.93403513	968.7290085	POS
PS(47:2)	PS	M+H	C53 H101 O10 N1 P1	16.16	942.7157635	POS
PS(49:1)	PS	M+Na	C55 H106 O10 N1 P1 Na1	15.74	994.7446585	POS
PS(49:2)	PS	M+H	C55 H105 O10 N1 P1	15.61006363	970.7470635	POS
PS(51:0)	PS	M+Na	C57 H112 O10 N1 P1 Na1	19.197	1024.791608	POS

PS(51:2)	PS	M+H	C57 H109 O10 N1 P1	12.741	998.7783635	POS
PS(51:3)	PS	M+H	C57 H107 O10 N1 P1	14.522	996.7627135	POS
PS(53:3)	PS	M+H	C59 H111 O10 N1 P1	14.755	1024.794013	POS
SM(d34:1)	SM	M+H	C39 H80 O6 N2 P1	13.13177338	703.5748525	POS
SM(d34:2)	SM	M+H	C39 H78 O6 N2 P1	12.51398088	701.5592025	POS
SM(d36:1)	SM	M+H	C41 H84 O6 N2 P1	14.62185973	731.6061525	POS
SM(d36:2)	SM	M+H	C41 H82 O6 N2 P1	14.05863639	729.5905025	POS
SM(d41:7)	SM	M+H	C46 H82 O6 N2 P1	9.140149374	789.5905025	POS
SPH(d14:2)	SPH	M+H	C14 H28 O2 N1	5.054677347	242.2114555	POS
SPH(d15:2)	SPH	M+H	C15 H30 O2 N1	6.148	256.2271055	POS
SPH(d16:0)	SPH	M+H	C16 H36 O2 N1	1.497	274.2740555	POS
SPH(d16:2)	SPH	M+H	C16 H32 O2 N1	7.264896087	270.2427555	POS
SPH(d17:0)	SPH	M+H	C17 H38 O2 N1	1.708411753	288.2897055	POS
SPH(d18:0)	SPH	M+H	C18 H40 O2 N1	3.243691517	302.3053555	POS
SPH(d18:1)	SPH	M+H	C18 H38 O2 N1	2.712040142	300.2897055	POS
SPH(d18:2)	SPH	M+H	C18 H36 O2 N1	2.179	298.2740555	POS
SPH(d19:0)	SPH	M+H	C19 H42 O2 N1	2.477	316.3210055	POS
SPH(d20:2)	SPH	M+H	C20 H40 O2 N1	11.045	326.3053555	POS
SPH(m18:0)	SPH	M+H	C18 H40 O1 N1	3.718316379	286.3104405	POS
SPH(m20:0)	SPH	M+H	C20 H44 O1 N1	4.775	314.3417405	POS
SPH(t16:0)	SPH	M+H	C16 H36 O3 N1	2.719	290.2689705	POS

SPH(t17:0)	SPH	M+H	C17 H38 O3 N1	1.816	304.2846205	POS
SPH(t18:0)	SPH	M+H	C18 H40 O3 N1	5.837046155	318.3002705	POS
SPHP(d21:2)	SPHP	M+CH3COO	C23 H45 O7 N1 P1	1.106002257	478.2939155	POS
SQDG(16:0/16:0)	SQDG	M-H	C41 H77 O12 S1	13.06203224	793.5141255	NEG
SQDG(16:0/18:1)	SQDG	M-H	C43 H79 O12 S1	13.13005967	819.5297755	NEG
SQDG(18:2/18:2)	SQDG	M-H	C45 H77 O12 S1	11.083	841.5141255	NEG
SQDG(28:0e)	SQDG	M-H	C37 H71 O11 S1	8.407	723.4722605	NEG
SQDG(30:1)	SQDG	M+CH3COO	C41 H75 O14 S1	4.608439037	823.4883055	POS
SQDG(32:1)	SQDG	M-H	C41 H75 O12 S1	6.34895698	791.4984755	NEG
SQDG(32:2)	SQDG	M+CH3COO	C43 H77 O14 S1	4.86693286	849.5039555	POS
SQDG(32:2e)	SQDG	M-H	C41 H75 O11 S1	8.308	775.5035605	NEG
SQDG(32:3)	SQDG	M+CH3COO	C43 H75 O14 S1	7.643771514	847.4883055	POS
SQDG(32:4)	SQDG	M+HCOO	C42 H71 O14 S1	10.46069713	831.4570055	POS
SQDG(34:0e)	SQDG	M+HCOO	C44 H85 O13 S1	8.069	853.5716405	POS
SQDG(34:1e)	SQDG	M-H	C43 H81 O11 S1	8.036219766	805.5505105	NEG
SQDG(34:2)	SQDG	M-H	C43 H77 O12 S1	12.06106195	817.5141255	NEG
SQDG(34:3)	SQDG	M-H	C43 H75 O12 S1	5.145147176	815.4984755	NEG
SQDG(34:3e)	SQDG	M-H	C43 H77 O11 S1	8.319691078	801.5192105	NEG
SQDG(34:4e)	SQDG	M-H	C43 H75 O11 S1	6.899202638	799.5035605	NEG
SQDG(36:0e)	SQDG	M+CH3COO	C47 H91 O13 S1	11.533	895.6185905	POS
SQDG(36:1e)	SQDG	M+HCOO	C46 H87 O13 S1	7.882	879.5872905	POS

SQDG(36:2e)	SQDG	M-H	C45 H83 O11 S1	8.104227741	831.5661605	NEG
SQDG(36:3)	SQDG	M-H	C45 H79 O12 S1	12.13198806	843.5297755	NEG
SQDG(37:0)	SQDG	M+CH3COO	C48 H91 O14 S1	7.765666856	923.6135055	POS
SQDG(37:3)	SQDG	M+CH3COO	C48 H85 O14 S1	8.109456314	917.5665555	POS
SQDG(38:1e)	SQDG	M-H	C47 H89 O11 S1	10.318	861.6131105	NEG
SQDG(38:2e)	SQDG	M-H	C47 H87 O11 S1	9.643	859.5974605	NEG
SQDG(42:0)	SQDG	M-H	C51 H97 O12 S1	14.666	933.6706255	NEG
SQDG(43:11)	SQDG	M-H	C52 H77 O12 S1	11.247	925.5141255	NEG
SQDG(44:0)	SQDG	M-H	C53 H101 O12 S1	14.463	961.7019255	NEG
SQDG(44:1)	SQDG	M+CH3COO	C55 H103 O14 S1	12.97	1019.707406	POS
SQDG(44:2)	SQDG	M+CH3COO	C55 H101 O14 S1	12.212	1017.691756	POS
SQDG(45:2)	SQDG	M+HCOO	C55 H101 O14 S1	12.491	1017.691756	POS
SQDG(46:1)	SQDG	M-H	C55 H103 O12 S1	15.989	987.7175755	NEG
SQDG(46:2)	SQDG	M-H	C55 H101 O12 S1	14.48718428	985.7019255	NEG
SQDG(46:4)	SQDG	M-H	C55 H97 O12 S1	13.582	981.6706255	NEG
SQDG(47:0)	SQDG	M-H	C56 H107 O12 S1	18.7288154	1003.748876	NEG
SQDG(48:0)	SQDG	M-H	C57 H109 O12 S1	16.675	1017.764526	NEG
SQDG(48:1)	SQDG	M+HCOO	C58 H109 O14 S1	15.511	1061.754356	POS
SQDG(49:0)	SQDG	M-H	C58 H111 O12 S1	19.19007327	1031.780176	NEG
SQDG(49:1)	SQDG	M-H	C58 H109 O12 S1	18.6964356	1029.764526	NEG
SQDG(62:1)	SQDG	M-H	C71 H135 O12 S1	19.879	1211.967976	NEG

SiE(16:0)	SiE	M+NH4	C45 H84 O2 N1	21.74909818	670.6496555	POS
SiE(18:1)	SiE	M+NH4	C47 H86 O2 N1	21.70318867	696.6653055	POS
SiE(18:2)	SiE	M+H	C47 H81 O2	19.37055715	677.6231065	POS
SiE(18:3)	SiE	M+H	C47 H79 O2	18.932	675.6074565	POS
SiE(23:6)	SiE	M+NH4	C52 H86 O2 N1	17.276	756.6653055	POS
StE(0:0)	StE	M+H	C29 H49 O1	9.14566378	413.3777915	POS
StE(16:0)	StE	M+H	C45 H79 O2	20.39105059	651.6074565	POS
StE(18:1)	StE	M+H	C47 H81 O2	19.396	677.6231065	POS
StE(18:2)	StE	M+H	C47 H79 O2	19.00961903	675.6074565	POS
StE(18:3)	StE	M+H	C47 H77 O2	18.45040631	673.5918065	POS
StE(22:5)	StE	M+NH4	C51 H84 O2 N1	16.832	742.6496555	POS
StE(22:6)	StE	M+NH4	C51 H82 O2 N1	17.30827536	740.6340055	POS
StE(30:6)	StE	M+NH4	C59 H98 O2 N1	19.77948569	852.7592055	POS
TG(10:0/10:0/13:0)	TG	M+H	C36 H69 O6	7.05	597.5088665	POS
TG(10:0/10:0/18:1)	TG	M+NH4	C41 H80 O6 N1	14.48710431	682.5980155	POS
TG(10:0/10:0/18:2)	TG	M+NH4	C41 H78 O6 N1	13.60462978	680.5823655	POS
TG(10:0/10:1/10:1)	TG	M+NH4	C33 H62 O6 N1	10.008	568.4571655	POS
TG(10:0/10:2/10:3)	TG	M+H	C33 H53 O6	15.91902098	545.3836665	POS
TG(10:0/10:3/11:3)	TG	M+H	C34 H53 O6	6.840099287	557.3836665	POS
TG(10:0/10:3/14:0)	TG	M+H	C37 H65 O6	11.048	605.4775665	POS
TG(10:0/11:2/12:4)	TG	M+Na	C36 H56 O6 Na1	5.219	607.3969115	POS

TG(10:0/18:1/18:1)	TG	M+Na	C49 H90 O6 Na1	18.750438 41	797.66296 15	POS
TG(10:0/18:1/18:2)	TG	M+NH4	C49 H92 O6 N1	19.452314 93	790.69191 55	POS
TG(10:0/18:1/23:0)	TG	M+NH4	C54 H106 O6 N1	20.306	864.80146 55	POS
TG(10:0/18:3/18:3)	TG	M+NH4	C49 H86 O6 N1	17.829278	784.64496 55	POS
TG(10:0/24:0/24:0)	TG	M+Na	C61 H118 O6 Na1	22.164720 39	969.88206 15	POS
TG(11:0/10:1/10:1)	TG	M+NH4	C34 H64 O6 N1	8.883	582.47281 55	POS
TG(11:0/10:2/10:2)	TG	M+Na	C34 H56 O6 Na1	10.393905 01	583.39691 15	POS
TG(11:0/10:2/12:2)	TG	M+H	C36 H61 O6	4.0087506 04	589.44626 65	POS
TG(11:0/11:0/11:2)	TG	M+Na	C36 H64 O6 Na1	8.2948323 78	615.45951 15	POS
TG(11:0/11:0/12:2)	TG	M+Na	C37 H66 O6 Na1	16.995335 59	629.47516 15	POS
TG(11:0/11:2/11:2)	TG	M+H	C36 H61 O6	11.389	589.44626 65	POS
TG(11:0/11:4/12:4)	TG	M+Na	C37 H54 O6 Na1	4.83	617.38126 15	POS
TG(11:0/16:0/16:0)	TG	M+Na	C46 H88 O6 Na1	18.127	759.64731 15	POS
TG(11:0/16:0/18:1)	TG	M+NH4	C48 H94 O6 N1	20.264299 31	780.70756 55	POS
TG(11:0/16:0/22:6)	TG	M+H	C52 H89 O6	17.933704 67	809.66536 65	POS
TG(11:0/18:1/18:2)	TG	M+NH4	C50 H94 O6 N1	19.853379 66	804.70756 55	POS
TG(11:0/18:1/22:6)	TG	M+H	C54 H91 O6	17.939618 97	835.68101 65	POS
TG(11:0/6:0/10:1)	TG	M+NH4	C30 H58 O6 N1	8.419	528.42586 55	POS
TG(11:0/6:0/10:2)	TG	M+H	C30 H53 O6	7.9911000 66	509.38366 65	POS
TG(11:0/6:0/11:3)	TG	M+H	C31 H53 O6	9.286	521.38366 65	POS
TG(11:0/6:0/11:4)	TG	M+H	C31 H51 O6	7.731	519.36801 65	POS

TG(11:0/6:0/6:0)	TG	M+NH4	C26 H52 O6 N1	6.046	474.37891 55	POS
TG(11:0/6:0/8:0)	TG	M+Na	C28 H52 O6 Na1	14.931	507.36561 15	POS
TG(12:0/10:1/11:2)	TG	M+H	C36 H63 O6	6.4310938 21	591.46191 65	POS
TG(12:0/11:3/12:0)	TG	M+NH4	C38 H70 O6 N1	11.322	636.51976 55	POS
TG(12:0/18:2/18:2)	TG	M+NH4	C51 H94 O6 N1	19.493385 89	816.70756 55	POS
TG(12:0/18:2/18:3)	TG	M+NH4	C51 H92 O6 N1	19.015648 28	814.69191 55	POS
TG(12:0/22:0/22:0)	TG	M+Na	C59 H114 O6 Na1	21.898	941.85076 15	POS
TG(12:0e/10:0/10:2)	TG	M+H	C35 H65 O5	12.224	565.48265 15	POS
TG(12:0e/10:0/11:2)	TG	M+H	C36 H67 O5	9.767	579.49830 15	POS
TG(12:0e/10:1/11:2)	TG	M+H	C36 H65 O5	10.603669 2	577.48265 15	POS
TG(12:0e/10:1/11:3)	TG	M+H	C36 H63 O5	9.153	575.46700 15	POS
TG(12:0e/10:1/12:2)	TG	M+H	C37 H67 O5	12.614042 24	591.49830 15	POS
TG(12:0e/10:2/10:2)	TG	M+H	C35 H61 O5	9.138	561.45135 15	POS
TG(12:0e/10:2/11:2)	TG	M+NH4	C36 H66 O5 N1	11.03	592.49355 05	POS
TG(12:0e/10:2/11:3)	TG	M+H	C36 H61 O5	10.738	573.45135 15	POS
TG(12:0e/10:2/12:2)	TG	M+H	C37 H65 O5	11.380159 71	589.48265 15	POS
TG(12:0e/10:2/12:3)	TG	M+H	C37 H63 O5	9.2737819 18	587.46700 15	POS
TG(12:0e/10:3/11:2)	TG	M+H	C36 H61 O5	8.8135578 12	573.45135 15	POS
TG(12:0e/10:3/11:3)	TG	M+H	C36 H59 O5	9.7219853 13	571.43570 15	POS
TG(12:0e/10:3/22:1)	TG	M+H	C47 H85 O5	14.691	729.63915 15	POS
TG(12:0e/11:3/12:4)	TG	M+Na	C38 H60 O5 Na1	3.065	619.43329 65	POS

TG(12:0e/12:0/18:4)	TG	M+H	C45 H81 O5	20.39211702	701.6078515	POS
TG(12:0e/12:4/20:4)	TG	M+NH4	C47 H80 O5 N1	15.08	738.6031005	POS
TG(12:0e/6:0/10:1)	TG	M+Na	C31 H58 O5 Na1	15.63	533.4176465	POS
TG(12:0e/6:0/10:2)	TG	M+Na	C31 H56 O5 Na1	15.859	531.4019965	POS
TG(12:0e/6:0/10:3)	TG	M+H	C31 H55 O5	18.19825253	507.4044015	POS
TG(12:0e/6:0/11:1)	TG	M+H	C32 H61 O5	10.20448735	525.4513515	POS
TG(12:0e/6:0/11:2)	TG	M+Na	C32 H58 O5 Na1	10.368	545.4176465	POS
TG(12:0e/6:0/11:3)	TG	M+H	C32 H57 O5	10.11002125	521.4200515	POS
TG(12:0e/6:0/11:4)	TG	M+H	C32 H55 O5	8.987582785	519.4044015	POS
TG(12:0e/6:0/12:3)	TG	M+H	C33 H59 O5	9.469	535.4357015	POS
TG(12:0e/6:0/18:1)	TG	M+H	C39 H75 O5	17.809	623.5609015	POS
TG(12:0e/6:0/18:2)	TG	M+H	C39 H73 O5	15.00950419	621.5452515	POS
TG(12:0e/6:0/18:3)	TG	M+H	C39 H71 O5	12.899	619.5296015	POS
TG(12:0e/6:0/20:5)	TG	M+H	C41 H71 O5	17.02	643.5296015	POS
TG(12:0e/6:0/6:0)	TG	M+Na	C27 H52 O5 Na1	12.477	479.3706965	POS
TG(12:0e/6:0/8:0)	TG	M+Na	C29 H56 O5 Na1	14.831	507.4019965	POS
TG(12:0e/8:0/18:3)	TG	M+H	C41 H75 O5	14.07544561	647.5609015	POS
TG(12:0e/9:0/10:1)	TG	M+Na	C34 H64 O5 Na1	7.21	575.4645965	POS
TG(12:0e/9:0/10:2)	TG	M+NH4	C34 H66 O5 N1	10.63009473	568.4935505	POS
TG(12:0e/9:0/10:3)	TG	M+NH4	C34 H64 O5 N1	10.2845999	566.4779005	POS
TG(12:0e/9:0/9:0)	TG	M+Na	C33 H64 O5 Na1	10.95401444	563.4645965	POS

TG(12:1e/10:1/10:1)	TG	M+H	C35 H63 O5	11.16516406	563.4670015	POS
TG(12:1e/10:1/12:1)	TG	M+H	C37 H67 O5	20.304	591.4983015	POS
TG(12:1e/10:2/11:2)	TG	M+H	C36 H61 O5	2.181	573.4513515	POS
TG(12:1e/10:3/11:3)	TG	M+Na	C36 H56 O5 Na1	8.794073603	591.4019965	POS
TG(12:1e/11:0/11:0)	TG	M+Na	C37 H70 O5 Na1	24.327	617.5115465	POS
TG(12:1e/11:1/11:2)	TG	M+H	C37 H65 O5	19.37217493	589.4826515	POS
TG(12:1e/11:2/14:4)	TG	M+H	C40 H65 O5	10.23151573	625.4826515	POS
TG(12:1e/11:3/11:4)	TG	M+Na	C37 H56 O5 Na1	8.062113709	603.4019965	POS
TG(12:1e/11:3/12:4)	TG	M+Na	C38 H58 O5 Na1	3.417	617.4176465	POS
TG(12:1e/11:4/18:1)	TG	M+NH4	C44 H78 O5 N1	16.691	700.5874505	POS
TG(12:1e/12:3/12:3)	TG	M+NH4	C39 H66 O5 N1	13.27504084	628.4935505	POS
TG(12:1e/14:4/16:0)	TG	M+H	C45 H79 O5	17.74571734	699.5922015	POS
TG(12:1e/18:1/18:1)	TG	M+NH4	C51 H98 O5 N1	20.963955	804.7439505	POS
TG(12:1e/6:0/10:1)	TG	M+Na	C31 H56 O5 Na1	10.37249849	531.4019965	POS
TG(12:1e/6:0/10:2)	TG	M+H	C31 H55 O5	12.27458834	507.4044015	POS
TG(12:1e/6:0/10:3)	TG	M+H	C31 H53 O5	17.16422395	505.3887515	POS
TG(12:1e/6:0/10:4)	TG	M+H	C31 H51 O5	11.39396858	503.3731015	POS
TG(12:1e/6:0/11:1)	TG	M+H	C32 H59 O5	10.716	523.4357015	POS
TG(12:1e/6:0/11:2)	TG	M+H	C32 H57 O5	8.304	521.4200515	POS
TG(12:1e/6:0/11:3)	TG	M+H	C32 H55 O5	14.62171253	519.4044015	POS
TG(12:1e/6:0/12:3)	TG	M+H	C33 H57 O5	11.47923099	533.4200515	POS

TG(12:1e/6:0/12:4)	TG	M+H	C33 H55 O5	18.258164 26	531.40440 15	POS
TG(12:1e/6:0/13:0)	TG	M+H	C34 H65 O5	11.732318 04	553.48265 15	POS
TG(12:1e/6:0/15:0)	TG	M+H	C36 H69 O5	7.517	581.51395 15	POS
TG(12:1e/6:0/16:0)	TG	M+NH4	C37 H74 O5 N1	21.386	612.55615 05	POS
TG(12:1e/6:0/18:1)	TG	M+H	C39 H73 O5	16.803851 73	621.54525 15	POS
TG(12:1e/6:0/18:2)	TG	M+H	C39 H71 O5	12.486931 36	619.52960 15	POS
TG(12:1e/6:0/18:3)	TG	M+H	C39 H69 O5	11.548437 37	617.51395 15	POS
TG(12:1e/6:0/18:4)	TG	M+H	C39 H67 O5	11.598193 99	615.49830 15	POS
TG(12:1e/6:0/6:0)	TG	M+Na	C27 H50 O5 Na1	11.37	477.35504 65	POS
TG(12:1e/6:0/9:0)	TG	M+Na	C30 H56 O5 Na1	14.668	519.40199 65	POS
TG(12:1e/8:0/8:0)	TG	M+Na	C31 H58 O5 Na1	14.972	533.41764 65	POS
TG(12:1e/9:0/10:1)	TG	M+NH4	C34 H66 O5 N1	7.295	568.49355 05	POS
TG(12:1e/9:0/10:2)	TG	M+H	C34 H61 O5	11.091108 73	549.45135 15	POS
TG(12:1e/9:0/12:1)	TG	M+NH4	C36 H70 O5 N1	13.464292	596.52485 05	POS
TG(12:1e/9:0/12:3)	TG	M+H	C36 H63 O5	9.6562302 75	575.46700 15	POS
TG(13:0/10:1/10:1)	TG	M+H	C36 H65 O6	9.5767308 97	593.47756 65	POS
TG(13:0/10:1/10:2)	TG	M+H	C36 H63 O6	5.1916371 47	591.46191 65	POS
TG(13:0/10:2/10:2)	TG	M+H	C36 H61 O6	5.3854177 97	589.44626 65	POS
TG(13:0/10:3/10:3)	TG	M+H	C36 H57 O6	11.474191 34	585.41496 65	POS
TG(13:0/11:2/12:3)	TG	M+H	C39 H65 O6	17.827	629.47756 65	POS
TG(13:0/11:3/11:3)	TG	M+H	C38 H61 O6	2.181	613.44626 65	POS

TG(14:0/10:2/12:3)	TG	M+H	C39 H65 O6	9.121	629.4775665	POS
TG(14:0/11:2/18:2)	TG	M+NH4	C46 H84 O6 N1	15.03289904	746.6293155	POS
TG(14:0/14:0/20:2)	TG	M+NH4	C51 H98 O6 N1	22.698	820.7388655	POS
TG(14:0/14:0/22:3)	TG	M+NH4	C53 H100 O6 N1	25.36205994	846.7545155	POS
TG(14:0/14:0/22:5)	TG	M+H	C53 H93 O6	17.41109421	825.6966665	POS
TG(14:0/14:0/22:6)	TG	M+H	C53 H91 O6	17.4826481	823.6810165	POS
TG(14:0/18:2/18:2)	TG	M+NH4	C53 H98 O6 N1	20.30367516	844.7388655	POS
TG(14:0/18:2/18:3)	TG	M+H	C53 H93 O6	17.69608214	825.6966665	POS
TG(14:0/18:2/20:4)	TG	M+H	C55 H95 O6	20.42027321	851.7123165	POS
TG(14:0/18:3/18:3)	TG	M+NH4	C53 H94 O6 N1	19.15796301	840.7075655	POS
TG(14:0/20:2/22:3)	TG	M+H	C59 H105 O6	25.59063458	909.7905665	POS
TG(14:0/22:3/22:3)	TG	M+H	C61 H107 O6	18.786	935.8062165	POS
TG(14:0/22:4/22:4)	TG	M+Na	C61 H102 O6 Na1	16.972	953.7568615	POS
TG(14:0e/10:0/10:1)	TG	M+Na	C37 H70 O5 Na1	17.5046615	617.5115465	POS
TG(14:0e/10:1/10:1)	TG	M+Na	C37 H68 O5 Na1	22.717	615.4958965	POS
TG(14:0e/10:1/10:2)	TG	M+H	C37 H67 O5	15.33824123	591.4983015	POS
TG(14:0e/10:1/11:3)	TG	M+H	C38 H67 O5	10.414	603.4983015	POS
TG(14:0e/10:2/10:2)	TG	M+H	C37 H65 O5	15.4190217	589.4826515	POS
TG(14:0e/10:2/10:3)	TG	M+H	C37 H63 O5	17.795	587.4670015	POS
TG(14:0e/10:2/11:3)	TG	M+Na	C38 H64 O5 Na1	13.057	623.4645965	POS
TG(14:0e/10:2/18:2)	TG	M+Na	C45 H80 O5 Na1	16.88714532	723.5897965	POS

TG(14:0e/10:3/11:3)	TG	M+H	C38 H63 O5	8.156	599.46700 15	POS
TG(14:0e/10:4/11:3)	TG	M+H	C38 H61 O5	8.295	597.45135 15	POS
TG(14:0e/11:0/11:3)	TG	M+H	C39 H71 O5	20.005136 48	619.52960 15	POS
TG(14:0e/11:1/11:2)	TG	M+H	C39 H71 O5	21.12	619.52960 15	POS
TG(14:0e/11:3/11:3)	TG	M+H	C39 H65 O5	10.398330 61	613.48265 15	POS
TG(14:0e/11:4/18:1)	TG	M+H	C46 H81 O5	17.585314 45	713.60785 15	POS
TG(14:0e/11:4/18:2)	TG	M+H	C46 H79 O5	16.600287 01	711.59220 15	POS
TG(14:0e/11:4/18:3)	TG	M+NH4	C46 H80 O5 N1	15.322	726.60310 05	POS
TG(14:0e/16:0/17:1)	TG	M+NH4	C50 H100 O5 N1	19.520892 67	794.75960 05	POS
TG(14:0e/17:1/18:2)	TG	M+NH4	C52 H100 O5 N1	19.031350 32	818.75960 05	POS
TG(14:0e/6:0/11:1)	TG	M+H	C34 H65 O5	11.451806 23	553.48265 15	POS
TG(14:0e/6:0/11:2)	TG	M+H	C34 H63 O5	12.586346 05	551.46700 15	POS
TG(14:0e/6:0/11:3)	TG	M+H	C34 H61 O5	11.698907 15	549.45135 15	POS
TG(14:0e/6:0/12:2)	TG	M+Na	C35 H64 O5 Na1	10.537	587.46459 65	POS
TG(14:0e/6:0/12:3)	TG	M+H	C35 H63 O5	14.079084 16	563.46700 15	POS
TG(14:0e/6:0/18:2)	TG	M+H	C41 H77 O5	17.979	649.57655 15	POS
TG(14:0e/6:0/18:3)	TG	M+H	C41 H75 O5	20.235857 28	647.56090 15	POS
TG(14:0e/6:0/18:4)	TG	M+H	C41 H73 O5	19.864864 32	645.54525 15	POS
TG(14:0e/6:0/6:0)	TG	M+Na	C29 H56 O5 Na1	11.359	507.40199 65	POS
TG(14:0e/6:0/9:0)	TG	M+H	C32 H63 O5	11.047358	527.46700 15	POS
TG(14:0e/8:0/9:0)	TG	M+H	C34 H67 O5	12.880459 6	555.49830 15	POS

TG(14:0e/9:0/10:0)	TG	M+H	C36 H71 O5	13.98870077	583.5296015	POS
TG(14:0e/9:0/10:1)	TG	M+NH4	C36 H72 O5 N1	12.59070477	598.5405005	POS
TG(14:0e/9:0/10:2)	TG	M+H	C36 H67 O5	11.42548466	579.4983015	POS
TG(14:0e/9:0/10:3)	TG	M+H	C36 H65 O5	8.522236406	577.4826515	POS
TG(14:0e/9:0/11:2)	TG	M+H	C37 H69 O5	19.99605679	593.5139515	POS
TG(14:1/10:3/10:3)	TG	M+Na	C37 H56 O6 Na1	6.768	619.3969115	POS
TG(14:1/10:4/11:4)	TG	M+H	C38 H55 O6	5.239	607.3993165	POS
TG(14:1/11:4/11:4)	TG	M+NH4	C39 H60 O6 N1	6.659	638.4415155	POS
TG(14:1e/10:0/10:0)	TG	M+Na	C37 H70 O5 Na1	22.555	617.5115465	POS
TG(14:1e/10:0/10:1)	TG	M+Na	C37 H68 O5 Na1	16.685	615.4958965	POS
TG(14:1e/10:1/10:1)	TG	M+H	C37 H67 O5	16.7808212	591.4983015	POS
TG(14:1e/10:1/12:2)	TG	M+H	C39 H69 O5	17.676	617.5139515	POS
TG(14:1e/10:1/12:3)	TG	M+H	C39 H67 O5	17.03345082	615.4983015	POS
TG(14:1e/10:2/12:1)	TG	M+H	C39 H69 O5	21.134	617.5139515	POS
TG(14:1e/10:2/12:2)	TG	M+H	C39 H67 O5	20.70966835	615.4983015	POS
TG(14:1e/10:2/12:3)	TG	M+H	C39 H65 O5	9.485753523	613.4826515	POS
TG(14:1e/10:3/12:1)	TG	M+H	C39 H67 O5	16.38850295	615.4983015	POS
TG(14:1e/10:3/12:2)	TG	M+H	C39 H65 O5	7.799477683	613.4826515	POS
TG(14:1e/10:3/16:0)	TG	M+Na	C43 H76 O5 Na1	13.093	695.5584965	POS
TG(14:1e/10:4/11:2)	TG	M+H	C38 H61 O5	10.143	597.4513515	POS
TG(14:1e/11:0/11:3)	TG	M+H	C39 H69 O5	19.53920825	617.5139515	POS

TG(14:1e/11:1/11:3)	TG	M+H	C39 H67 O5	15.63532711	615.4983015	POS
TG(14:1e/11:2/11:3)	TG	M+H	C39 H65 O5	16.63694418	613.4826515	POS
TG(14:1e/11:3/11:3)	TG	M+H	C39 H63 O5	13.803	611.4670015	POS
TG(14:1e/11:3/12:3)	TG	M+H	C40 H65 O5	10.8857107	625.4826515	POS
TG(14:1e/6:0/10:3)	TG	M+H	C33 H57 O5	10.37	533.4200515	POS
TG(14:1e/6:0/11:1)	TG	M+NH4	C34 H66 O5 N1	12.11192125	568.4935505	POS
TG(14:1e/6:0/11:2)	TG	M+H	C34 H61 O5	9.192957777	549.4513515	POS
TG(14:1e/6:0/11:3)	TG	M+H	C34 H59 O5	10.71590867	547.4357015	POS
TG(14:1e/6:0/11:4)	TG	M+H	C34 H57 O5	8.976	545.4200515	POS
TG(14:1e/6:0/18:2)	TG	M+H	C41 H75 O5	16.92886726	647.5609015	POS
TG(14:1e/6:0/18:3)	TG	M+H	C41 H73 O5	14.03845585	645.5452515	POS
TG(14:1e/6:0/18:4)	TG	M+H	C41 H71 O5	12.75944266	643.5296015	POS
TG(14:1e/6:0/6:0)	TG	M+Na	C29 H54 O5 Na1	11.278	505.3863465	POS
TG(14:1e/6:0/9:0)	TG	M+NH4	C32 H64 O5 N1	11.29568267	542.4779005	POS
TG(14:1e/8:0/11:1)	TG	M+H	C36 H67 O5	11.77434794	579.4983015	POS
TG(14:1e/8:0/9:0)	TG	M+H	C34 H65 O5	13.11909755	553.4826515	POS
TG(14:1e/9:0/10:1)	TG	M+H	C36 H67 O5	12.81428056	579.4983015	POS
TG(14:1e/9:0/10:2)	TG	M+NH4	C36 H68 O5 N1	12.13142294	594.5092005	POS
TG(14:1e/9:0/11:0)	TG	M+Na	C37 H70 O5 Na1	21.10107478	617.5115465	POS
TG(14:1e/9:0/11:2)	TG	M+H	C37 H67 O5	16.37565546	591.4983015	POS
TG(14:1e/9:0/11:3)	TG	M+H	C37 H65 O5	9.249	589.4826515	POS

TG(14:1e/9:0/9:0)	TG	M+NH4	C35 H70 O5 N1	13.8205193	584.5248505	POS
TG(15:0/10:1/16:0)	TG	M+Na	C44 H82 O6 Na1	18.432	729.6003615	POS
TG(15:0/10:1/18:1)	TG	M+Na	C46 H84 O6 Na1	18.793	755.6160115	POS
TG(15:0/10:2/11:2)	TG	M+H	C39 H67 O6	14.187	631.4932165	POS
TG(15:0/10:2/11:3)	TG	M+H	C39 H65 O6	15.99037404	629.4775665	POS
TG(15:0/10:2/18:2)	TG	M+Na	C46 H80 O6 Na1	14.31360071	751.5847115	POS
TG(15:0/12:4/12:4)	TG	M+H	C42 H65 O6	12.419	665.4775665	POS
TG(15:0/15:0/19:0)	TG	M+Na	C52 H100 O6 Na1	20.669	843.7412115	POS
TG(15:0/15:0/22:6)	TG	M+Na	C55 H94 O6 Na1	13.67130555	873.6942615	POS
TG(15:0/16:0/16:0)	TG	M+NH4	C50 H100 O6 N1	21.09556893	810.7545155	POS
TG(15:0/16:0/18:1)	TG	M+NH4	C52 H102 O6 N1	21.06419493	836.7701655	POS
TG(15:0/16:0/18:2)	TG	M+NH4	C52 H100 O6 N1	18.72547986	834.7545155	POS
TG(15:0/16:0/20:5)	TG	M+H	C54 H95 O6	19.283	839.7123165	POS
TG(15:0/16:0/22:5)	TG	M+H	C56 H99 O6	18.553	867.7436165	POS
TG(15:0/16:1/18:2)	TG	M+NH4	C52 H98 O6 N1	18.61081733	832.7388655	POS
TG(15:0/17:0/17:0)	TG	M+NH4	C52 H104 O6 N1	20.33298624	838.7858155	POS
TG(15:0/17:0/18:1)	TG	M+NH4	C53 H104 O6 N1	25.753	850.7858155	POS
TG(15:0/18:1/18:1)	TG	M+NH4	C54 H104 O6 N1	21.08837942	862.7858155	POS
TG(15:0/18:1/18:3)	TG	M+NH4	C54 H100 O6 N1	18.72810956	858.7545155	POS
TG(15:0/18:2/18:2)	TG	M+NH4	C54 H100 O6 N1	20.3377558	858.7545155	POS
TG(15:0/18:2/18:3)	TG	M+NH4	C54 H98 O6 N1	19.90975485	856.7388655	POS

TG(15:0/18:3/18:3)	TG	M+NH4	C54 H96 O6 N1	19.47386983	854.7232155	POS
TG(15:0/6:0/10:2)	TG	M+NH4	C34 H64 O6 N1	8.32	582.4728155	POS
TG(15:0/6:0/12:1)	TG	M+NH4	C36 H70 O6 N1	12.01188882	612.5197655	POS
TG(15:0/6:0/12:2)	TG	M+H	C36 H65 O6	5.950115985	593.4775665	POS
TG(15:0/6:0/6:0)	TG	M+Na	C30 H56 O6 Na1	9.544	535.3969115	POS
TG(15:0/8:0/10:1)	TG	M+NH4	C36 H70 O6 N1	10.95456901	612.5197655	POS
TG(16:0/10:0/18:1)	TG	M+NH4	C47 H92 O6 N1	19.95088043	766.6919155	POS
TG(16:0/10:1/10:1)	TG	M+NH4	C39 H74 O6 N1	16.47192852	652.5510655	POS
TG(16:0/10:1/11:4)	TG	M+NH4	C40 H70 O6 N1	10.8886388	660.5197655	POS
TG(16:0/10:2/10:2)	TG	M+H	C39 H67 O6	12.299	631.4932165	POS
TG(16:0/10:2/10:3)	TG	M+H	C39 H65 O6	13.711	629.4775665	POS
TG(16:0/10:2/13:0)	TG	M+NH4	C42 H80 O6 N1	14.83264508	694.5980155	POS
TG(16:0/10:2/16:0)	TG	M+H	C45 H83 O6	17.38874143	719.6184165	POS
TG(16:0/10:2/18:3)	TG	M+NH4	C47 H84 O6 N1	15.72349299	758.6293155	POS
TG(16:0/10:3/11:3)	TG	M+H	C40 H65 O6	9.74883465	641.4775665	POS
TG(16:0/10:3/18:2)	TG	M+NH4	C47 H84 O6 N1	17.76799362	758.6293155	POS
TG(16:0/10:4/11:3)	TG	M+NH4	C40 H66 O6 N1	9.834	656.4884655	POS
TG(16:0/10:4/17:1)	TG	M+H	C46 H79 O6	15.77593496	727.5871165	POS
TG(16:0/11:1/16:0)	TG	M+Na	C46 H86 O6 Na1	17.447	757.6316615	POS
TG(16:0/11:2/16:0)	TG	M+Na	C46 H84 O6 Na1	12.641	755.6160115	POS
TG(16:0/11:2/18:1)	TG	M+H	C48 H87 O6	18.08237454	759.6497165	POS

TG(16:0/11:2/18:2)	TG	M+H	C48 H85 O6	16.513	757.6340665	POS
TG(16:0/11:2/18:3)	TG	M+H	C48 H83 O6	14.99184306	755.6184165	POS
TG(16:0/11:3/16:0)	TG	M+H	C46 H83 O6	19.30846567	731.6184165	POS
TG(16:0/11:3/18:1)	TG	M+Na	C48 H84 O6 Na1	15.04	779.6160115	POS
TG(16:0/11:3/18:2)	TG	M+NH4	C48 H86 O6 N1	14.41965415	772.6449655	POS
TG(16:0/11:3/18:3)	TG	M+H	C48 H81 O6	15.17707985	753.6027665	POS
TG(16:0/11:3/22:3)	TG	M+NH4	C52 H92 O6 N1	15.975	826.6919155	POS
TG(16:0/11:4/16:0)	TG	M+H	C46 H81 O6	17.07267624	729.6027665	POS
TG(16:0/11:4/16:1)	TG	M+H	C46 H79 O6	15.28074979	727.5871165	POS
TG(16:0/11:4/18:1)	TG	M+H	C48 H83 O6	18.21330791	755.6184165	POS
TG(16:0/12:0/14:0)	TG	M+NH4	C45 H90 O6 N1	19.973	740.6762655	POS
TG(16:0/12:1/16:0)	TG	M+Na	C47 H88 O6 Na1	18.784	771.6473115	POS
TG(16:0/12:2/16:0)	TG	M+Na	C47 H86 O6 Na1	18.1924538	769.6316615	POS
TG(16:0/12:2/18:3)	TG	M+H	C49 H85 O6	15.70436347	769.6340665	POS
TG(16:0/12:4/21:0)	TG	M+H	C52 H93 O6	18.095	813.6966665	POS
TG(16:0/13:0/16:0)	TG	M+NH4	C48 H96 O6 N1	20.689967	782.7232155	POS
TG(16:0/13:0/18:1)	TG	M+NH4	C50 H98 O6 N1	20.662039	808.7388655	POS
TG(16:0/13:0/18:2)	TG	M+NH4	C50 H96 O6 N1	20.24775205	806.7232155	POS
TG(16:0/13:0/20:4)	TG	M+NH4	C52 H96 O6 N1	20.57992678	830.7232155	POS
TG(16:0/14:0/18:2)	TG	M+NH4	C51 H98 O6 N1	20.43728178	820.7388655	POS
TG(16:0/14:0/18:3)	TG	M+H	C51 H93 O6	18.11994298	801.6966665	POS

TG(16:0/14:0/22:2)	TG	M+Na	C55 H102 O6 Na1	16.61102164	881.7568615	POS
TG(16:0/14:2/18:2)	TG	M+NH4	C51 H94 O6 N1	19.72787074	816.7075655	POS
TG(16:0/14:3/14:4)	TG	M+NH4	C47 H80 O6 N1	14.143	754.5980155	POS
TG(16:0/14:4/16:0)	TG	M+H	C49 H87 O6	18.74293221	771.6497165	POS
TG(16:0/16:0/16:0)	TG	M+NH4	C51 H102 O6 N1	21.31801666	824.7701655	POS
TG(16:0/16:0/19:0)	TG	M+NH4	C54 H108 O6 N1	21.76610499	866.8171155	POS
TG(16:0/16:0/21:0)	TG	M+NH4	C56 H112 O6 N1	22.05524538	894.8484155	POS
TG(16:0/16:0/22:0)	TG	M+NH4	C57 H114 O6 N1	22.19556185	908.8640655	POS
TG(16:0/16:0/22:2)	TG	M+Na	C57 H106 O6 Na1	19.308	909.7881615	POS
TG(16:0/16:0/22:4)	TG	M+Na	C57 H102 O6 Na1	18.299	905.7568615	POS
TG(16:0/16:0/22:5)	TG	M+H	C57 H101 O6	22.03	881.7592665	POS
TG(16:0/16:0/23:0)	TG	M+NH4	C58 H116 O6 N1	22.31327587	922.8797155	POS
TG(16:0/16:0/23:1)	TG	M+H	C58 H111 O6	18.066	903.8375165	POS
TG(16:0/16:0/24:0)	TG	M+NH4	C59 H118 O6 N1	22.4421559	936.8953655	POS
TG(16:0/16:0/24:1)	TG	M+NH4	C59 H116 O6 N1	22.55173245	934.8797155	POS
TG(16:0/16:1/18:2)	TG	M+NH4	C53 H100 O6 N1	24.02810057	846.7545155	POS
TG(16:0/17:0/18:2)	TG	M+Na	C54 H100 O6 Na1	18.529	867.7412115	POS
TG(16:0/18:1/19:0)	TG	M+NH4	C56 H110 O6 N1	21.744595	892.8327655	POS
TG(16:0/18:1/20:1)	TG	M+NH4	C57 H110 O6 N1	21.95306095	904.8327655	POS
TG(16:0/18:1/20:4)	TG	M+H	C57 H101 O6	7.646034125	881.7592665	POS
TG(16:0/18:1/21:0)	TG	M+NH4	C58 H114 O6 N1	22.02038007	920.8640655	POS

TG(16:0/18:1/22:0)	TG	M+NH4	C59 H116 O6 N1	22.16704386	934.8797155	POS
TG(16:0/18:1/22:2)	TG	M+Na	C59 H108 O6 Na1	18.79331396	935.8038115	POS
TG(16:0/18:1/22:5)	TG	M+H	C59 H103 O6	25.28440544	907.7749165	POS
TG(16:0/18:1/22:6)	TG	M+H	C59 H101 O6	18.29322859	905.7592665	POS
TG(16:0/18:1/23:0)	TG	M+NH4	C60 H118 O6 N1	22.28575563	948.8953655	POS
TG(16:0/18:1/24:0)	TG	M+NH4	C61 H120 O6 N1	22.4084377	962.9110155	POS
TG(16:0/18:1/24:1)	TG	M+NH4	C61 H118 O6 N1	26.045	960.8953655	POS
TG(16:0/18:2/20:2)	TG	M+Na	C57 H102 O6 Na1	17.462	905.7568615	POS
TG(16:0/18:2/22:3)	TG	M+H	C59 H105 O6	17.709	909.7905665	POS
TG(16:0/18:2/22:4)	TG	M+NH4	C59 H106 O6 N1	16.668	924.8014655	POS
TG(16:0/18:2/22:5)	TG	M+H	C59 H101 O6	17.46245423	905.7592665	POS
TG(16:0/18:2/22:6)	TG	M+H	C59 H99 O6	17.90602792	903.7436165	POS
TG(16:0/18:2/24:0)	TG	M+NH4	C61 H118 O6 N1	25.11674683	960.8953655	POS
TG(16:0/18:2/24:1)	TG	M+NH4	C61 H116 O6 N1	22.2531603	958.8797155	POS
TG(16:0/18:3/18:3)	TG	M+H	C55 H95 O6	15.23454176	851.7123165	POS
TG(16:0/18:3/20:3)	TG	M+H	C57 H99 O6	17.809	879.7436165	POS
TG(16:0/18:3/20:5)	TG	M+H	C57 H95 O6	20.186	875.7123165	POS
TG(16:0/18:3/22:5)	TG	M+H	C59 H99 O6	17.93399728	903.7436165	POS
TG(16:0/18:3/24:0)	TG	M+H	C61 H113 O6	20.75162239	941.8531665	POS
TG(16:0/20:3/20:3)	TG	M+H	C59 H103 O6	24.188	907.7749165	POS
TG(16:0/20:4/22:0)	TG	M+H	C61 H111 O6	22.215	939.8375165	POS

TG(16:0/22:4/24:2)	TG	M+H	C65 H115 O6	20.328013 28	991.86881 65	POS
TG(16:0/22:5/24:0)	TG	M+H	C65 H117 O6	21.011	993.88446 65	POS
TG(16:0/23:0/24:0)	TG	M+NH4	C66 H132 O6 N1	23.175642 01	1035.0049 15	POS
TG(16:0/24:0/24:0)	TG	M+NH4	C67 H134 O6 N1	23.260166 36	1049.0205 65	POS
TG(16:0/6:0/11:2)	TG	M+NH4	C36 H68 O6 N1	7.7788383 85	610.50411 55	POS
TG(16:0/6:0/11:3)	TG	M+NH4	C36 H66 O6 N1	10.565	608.48846 55	POS
TG(16:0/6:0/11:4)	TG	M+H	C36 H61 O6	9.1511120 08	589.44626 65	POS
TG(16:0/6:0/12:1)	TG	M+NH4	C37 H72 O6 N1	12.655990 39	626.53541 55	POS
TG(16:0/6:0/12:2)	TG	M+NH4	C37 H70 O6 N1	11.386992 43	624.51976 55	POS
TG(16:0/6:0/12:3)	TG	M+NH4	C37 H68 O6 N1	10.747	622.50411 55	POS
TG(16:0/6:0/13:0)	TG	M+NH4	C38 H76 O6 N1	16.339362 96	642.56671 55	POS
TG(16:0/6:0/14:0)	TG	M+NH4	C39 H78 O6 N1	18.044424 88	656.58236 55	POS
TG(16:0/6:0/14:1)	TG	M+NH4	C39 H76 O6 N1	17.434824 62	654.56671 55	POS
TG(16:0/6:0/14:4)	TG	M+H	C39 H67 O6	12.822375 03	631.49321 65	POS
TG(16:0/6:0/18:1)	TG	M+NH4	C43 H84 O6 N1	18.894227 06	710.62931 55	POS
TG(16:0/6:0/18:2)	TG	M+NH4	C43 H82 O6 N1	18.177472 68	708.61366 55	POS
TG(16:0/6:0/20:5)	TG	M+H	C45 H77 O6	15.993	713.57146 65	POS
TG(16:0/6:0/22:2)	TG	M+Na	C47 H86 O6 Na1	15.604	769.63166 15	POS
TG(16:0/6:0/22:6)	TG	M+H	C47 H79 O6	12.459	739.58711 65	POS
TG(16:0/6:0/6:0)	TG	M+Na	C31 H58 O6 Na1	7.747	549.41256 15	POS
TG(16:0/6:0/9:0)	TG	M+Na	C34 H64 O6 Na1	8.503	591.45951 15	POS

TG(16:0/8:0/10:0)	TG	M+NH4	C37 H74 O6 N1	13.2929073	628.5510655	POS
TG(16:0/8:0/10:1)	TG	M+NH4	C37 H72 O6 N1	13.36394125	626.5354155	POS
TG(16:0/8:0/10:2)	TG	M+H	C37 H67 O6	11.93960683	607.4932165	POS
TG(16:0/8:0/10:3)	TG	M+H	C37 H65 O6	10.4753223	605.4775665	POS
TG(16:0/8:0/11:1)	TG	M+H	C38 H71 O6	14.59261132	623.5245165	POS
TG(16:0/8:0/16:0)	TG	M+NH4	C43 H86 O6 N1	19.48304436	712.6449655	POS
TG(16:0/8:0/18:1)	TG	M+NH4	C45 H88 O6 N1	19.43950768	738.6606155	POS
TG(16:0/8:0/22:6)	TG	M+H	C49 H83 O6	15.2091255	767.6184165	POS
TG(16:0/8:0/8:0)	TG	M+Na	C35 H66 O6 Na1	8.715	605.4751615	POS
TG(16:0/9:0/16:0)	TG	M+Na	C44 H84 O6 Na1	18.91	731.6160115	POS
TG(16:0/9:0/16:1)	TG	M+NH4	C44 H86 O6 N1	19.13916005	724.6449655	POS
TG(16:0/9:0/18:1)	TG	M+NH4	C46 H90 O6 N1	19.71222678	752.6762655	POS
TG(16:0/9:0/18:2)	TG	M+NH4	C46 H88 O6 N1	19.14853768	750.6606155	POS
TG(16:0/9:0/20:4)	TG	M+H	C48 H85 O6	18.185	757.6340665	POS
TG(16:0/9:0/9:0)	TG	M+H	C37 H71 O6	13.63954249	611.5245165	POS
TG(16:0e/10:3/18:2)	TG	M+H	C47 H83 O5	14.577	727.6235015	POS
TG(16:0e/10:3/18:3)	TG	M+NH4	C47 H84 O5 N1	15.102	742.6344005	POS
TG(16:0e/11:4/18:3)	TG	M+NH4	C48 H84 O5 N1	14.072	754.6344005	POS
TG(16:0e/12:4/18:3)	TG	M+Na	C49 H82 O5 Na1	12.142	773.6054465	POS
TG(16:0e/18:4/18:4)	TG	M+H	C55 H93 O5	18.013	833.7017515	POS
TG(16:0e/6:0/12:2)	TG	M+Na	C37 H68 O5 Na1	11.722	615.4958965	POS

TG(16:0e/6:0/18:3)	TG	M+H	C43 H79 O5	15.357	675.5922015	POS
TG(16:0e/6:0/18:4)	TG	M+H	C43 H77 O5	20.34828718	673.5765515	POS
TG(16:0e/8:0/10:1)	TG	M+NH4	C37 H74 O5 N1	16.80719759	612.5561505	POS
TG(16:0e/9:0/10:1)	TG	M+NH4	C38 H76 O5 N1	15.36375216	626.5718005	POS
TG(16:0e/9:0/10:2)	TG	M+Na	C38 H70 O5 Na1	13.16	629.5115465	POS
TG(16:0e/9:0/9:0)	TG	M+Na	C37 H72 O5 Na1	15.717	619.5271965	POS
TG(16:1/11:1/22:6)	TG	M+H	C52 H85 O6	12.606	805.6340665	POS
TG(16:1/11:4/18:1)	TG	M+H	C48 H81 O6	15.74779726	753.6027665	POS
TG(16:1/11:4/18:2)	TG	M+H	C48 H79 O6	14.93087027	751.5871165	POS
TG(16:1/16:1/20:3)	TG	M+NH4	C55 H100 O6 N1	0.031	870.7545155	POS
TG(16:1/17:0/17:0)	TG	M+NH4	C53 H104 O6 N1	23.708	850.7858155	POS
TG(16:1/18:1/18:3)	TG	M+NH4	C55 H100 O6 N1	24.848	870.7545155	POS
TG(16:1/18:1/20:1)	TG	M+NH4	C57 H108 O6 N1	23.33392619	902.8171155	POS
TG(16:1/18:1/20:5)	TG	M+H	C57 H97 O6	15.884	877.7279665	POS
TG(16:1/18:2/18:2)	TG	M+NH4	C55 H100 O6 N1	20.02772519	870.7545155	POS
TG(16:1/18:2/18:3)	TG	M+NH4	C55 H98 O6 N1	19.62723261	868.7388655	POS
TG(16:1/18:2/22:6)	TG	M+H	C59 H97 O6	17.21376006	901.7279665	POS
TG(16:1/20:1/20:1)	TG	M+NH4	C59 H112 O6 N1	10.874	930.8484155	POS
TG(16:1/20:1/20:2)	TG	M+H	C59 H107 O6	19.358	911.8062165	POS
TG(16:1/20:2/20:2)	TG	M+H	C59 H105 O6	18.80488672	909.7905665	POS
TG(16:1/6:0/11:3)	TG	M+H	C36 H61 O6	7.683654519	589.4462665	POS

TG(16:1/6:0/11:4)	TG	M+H	C36 H59 O6	8.617	587.4306165	POS
TG(16:1/6:0/12:2)	TG	M+Na	C37 H64 O6 Na1	7.074113969	627.4595115	POS
TG(16:1/6:0/6:0)	TG	M+NH4	C31 H60 O6 N1	11.372	542.4415155	POS
TG(16:1e/10:2/10:2)	TG	M+H	C39 H67 O5	7.574	615.4983015	POS
TG(16:1e/10:2/10:3)	TG	M+H	C39 H65 O5	19.53694702	613.4826515	POS
TG(16:1e/10:3/18:1)	TG	M+H	C47 H83 O5	18.521	727.6235015	POS
TG(16:1e/17:1/18:1)	TG	M+NH4	C54 H104 O5 N1	19.56677583	846.7909005	POS
TG(16:1e/18:2/18:3)	TG	M+H	C55 H97 O5	18.88282559	837.7330515	POS
TG(16:1e/18:3/18:3)	TG	M+H	C55 H95 O5	18.28114011	835.7174015	POS
TG(16:1e/6:0/11:2)	TG	M+NH4	C36 H68 O5 N1	12.6420131	594.5092005	POS
TG(16:1e/6:0/11:3)	TG	M+NH4	C36 H66 O5 N1	11.45676178	592.4935505	POS
TG(16:1e/6:0/11:4)	TG	M+H	C36 H61 O5	11.681	573.4513515	POS
TG(16:1e/6:0/12:2)	TG	M+Na	C37 H66 O5 Na1	21.656	613.4802465	POS
TG(16:1e/6:0/6:0)	TG	M+Na	C31 H58 O5 Na1	11.441	533.4176465	POS
TG(16:1e/6:0/9:0)	TG	M+Na	C34 H64 O5 Na1	6.47509804	575.4645965	POS
TG(16:1e/8:0/10:0)	TG	M+Na	C37 H70 O5 Na1	12.428	617.5115465	POS
TG(16:1e/8:0/10:1)	TG	M+Na	C37 H68 O5 Na1	20.62561027	615.4958965	POS
TG(16:1e/9:0/10:1)	TG	M+NH4	C38 H74 O5 N1	14.501	624.5561505	POS
TG(16:1e/9:0/9:0)	TG	M+Na	C37 H70 O5 Na1	24.687	617.5115465	POS
TG(16:2e/10:0/10:1)	TG	M+H	C39 H71 O5	17.292	619.5296015	POS
TG(16:2e/10:1/10:1)	TG	M+H	C39 H69 O5	23.67805151	617.5139515	POS

TG(16:2e/10:1/10:2)	TG	M+H	C39 H67 O5	17.471	615.4983015	POS
TG(16:2e/10:2/10:2)	TG	M+H	C39 H65 O5	10.85600257	613.4826515	POS
TG(16:2e/10:2/10:3)	TG	M+H	C39 H63 O5	17.71554879	611.4670015	POS
TG(16:2e/10:2/11:3)	TG	M+H	C40 H65 O5	12.065	625.4826515	POS
TG(16:2e/10:2/11:4)	TG	M+H	C40 H63 O5	13.06083524	623.4670015	POS
TG(16:2e/10:3/10:3)	TG	M+Na	C39 H60 O5 Na1	11.815	631.4332965	POS
TG(16:2e/10:3/18:2)	TG	M+H	C47 H79 O5	16.87239542	723.5922015	POS
TG(16:2e/10:3/18:3)	TG	M+NH4	C47 H80 O5 N1	13.57590808	738.6031005	POS
TG(16:2e/11:3/18:3)	TG	M+NH4	C48 H82 O5 N1	13.8821781	752.6187505	POS
TG(16:2e/12:4/16:0)	TG	M+NH4	C47 H84 O5 N1	17.82822463	742.6344005	POS
TG(16:2e/18:3/18:3)	TG	M+H	C55 H93 O5	19.14584213	833.7017515	POS
TG(16:2e/6:0/10:2)	TG	M+H	C35 H61 O5	18.05	561.4513515	POS
TG(16:2e/6:0/11:1)	TG	M+NH4	C36 H68 O5 N1	11.54259344	594.5092005	POS
TG(16:2e/6:0/11:2)	TG	M+H	C36 H63 O5	7.694934868	575.4670015	POS
TG(16:2e/6:0/11:3)	TG	M+H	C36 H61 O5	11.036	573.4513515	POS
TG(16:2e/6:0/12:1)	TG	M+H	C37 H67 O5	22.112	591.4983015	POS
TG(16:2e/6:0/6:0)	TG	M+Na	C31 H56 O5 Na1	18.303	531.4019965	POS
TG(16:2e/8:0/10:1)	TG	M+H	C37 H67 O5	19.63941375	591.4983015	POS
TG(16:2e/8:0/9:0)	TG	M+H	C36 H67 O5	13.16734828	579.4983015	POS
TG(16:2e/9:0/9:0)	TG	M+Na	C37 H68 O5 Na1	23.631	615.4958965	POS
TG(17:0/10:3/12:4)	TG	M+Na	C42 H66 O6 Na1	11.32684066	689.4751615	POS

TG(17:0/17:1/18:1)	TG	M+NH4	C55 H106 O6 N1	23.449	876.8014655	POS
TG(17:0/6:0/10:0)	TG	M+NH4	C36 H72 O6 N1	9.482515977	614.5354155	POS
TG(17:0/6:0/10:1)	TG	M+H	C36 H67 O6	8.993907885	595.4932165	POS
TG(17:0/6:0/10:2)	TG	M+NH4	C36 H68 O6 N1	10.51862771	610.5041155	POS
TG(17:0/6:0/10:3)	TG	M+NH4	C36 H66 O6 N1	9.99252511	608.4884655	POS
TG(17:0/6:0/12:1)	TG	M+NH4	C38 H74 O6 N1	13.59075557	640.5510655	POS
TG(18:0/16:0/18:0)	TG	M+Na	C55 H106 O6 Na1	19.932	885.7881615	POS
TG(18:0/16:0/22:5)	TG	M+H	C59 H105 O6	19.29947166	909.7905665	POS
TG(18:0/16:0/24:0)	TG	M+NH4	C61 H122 O6 N1	22.67346023	964.9266655	POS
TG(18:0/18:0/18:3)	TG	M+H	C57 H105 O6	19.94349903	885.7905665	POS
TG(18:0/18:2/20:1)	TG	M+NH4	C59 H112 O6 N1	25.185	930.8484155	POS
TG(18:0/18:2/22:0)	TG	M+NH4	C61 H118 O6 N1	9.519	960.8953655	POS
TG(18:0/18:2/22:3)	TG	M+H	C61 H109 O6	25.305	937.8218665	POS
TG(18:0/18:3/20:0)	TG	M+H	C59 H109 O6	20.38513891	913.8218665	POS
TG(18:0/18:3/22:1)	TG	M+NH4	C61 H114 O6 N1	23.921	956.8640655	POS
TG(18:0/6:0/10:1)	TG	M+Na	C37 H68 O6 Na1	17.462	631.4908115	POS
TG(18:0/6:0/14:3)	TG	M+H	C41 H73 O6	15.09889911	661.5401665	POS
TG(18:0/8:0/10:1)	TG	M+NH4	C39 H76 O6 N1	14.1529408	654.5667155	POS
TG(18:0/8:0/10:2)	TG	M+H	C39 H71 O6	13.30331785	635.5245165	POS
TG(18:0/9:0/18:3)	TG	M+H	C48 H87 O6	19.80030204	759.6497165	POS
TG(18:0/9:0/9:0)	TG	M+NH4	C39 H78 O6 N1	15.00098557	656.5823655	POS

TG(18:0e/10:0/17:1)	TG	M+NH4	C48 H96 O5 N1	19.934	766.7283005	POS
TG(18:0e/10:1/10:2)	TG	M+H	C41 H75 O5	13.24368187	647.5609015	POS
TG(18:0e/16:0/18:1)	TG	M+NH4	C55 H110 O5 N1	22.02358969	864.8378505	POS
TG(18:0e/16:0/18:2)	TG	M+NH4	C55 H108 O5 N1	21.74486113	862.8222005	POS
TG(18:0e/16:0/18:4)	TG	M+Na	C55 H100 O5 Na1	18.099	863.7462965	POS
TG(18:0e/18:1/18:1)	TG	M+NH4	C57 H112 O5 N1	22.013	890.8535005	POS
TG(18:0e/18:1/18:2)	TG	M+NH4	C57 H110 O5 N1	21.72246735	888.8378505	POS
TG(18:0e/18:2/18:2)	TG	M+NH4	C57 H108 O5 N1	21.42766758	886.8222005	POS
TG(18:0e/6:0/10:0)	TG	M+Na	C37 H72 O5 Na1	19.991	619.5271965	POS
TG(18:0e/6:0/10:1)	TG	M+Na	C37 H70 O5 Na1	17.756	617.5115465	POS
TG(18:0e/6:0/10:2)	TG	M+Na	C37 H68 O5 Na1	18.95226315	615.4958965	POS
TG(18:0e/6:0/11:1)	TG	M+Na	C38 H72 O5 Na1	14.691	631.5271965	POS
TG(18:0e/6:0/11:2)	TG	M+NH4	C38 H74 O5 N1	15.41343038	624.5561505	POS
TG(18:0e/6:0/11:3)	TG	M+NH4	C38 H72 O5 N1	10.371	622.5405005	POS
TG(18:0e/6:0/18:3)	TG	M+H	C45 H83 O5	16.64723226	703.6235015	POS
TG(18:0e/8:0/10:0)	TG	M+Na	C39 H76 O5 Na1	15.002	647.5584965	POS
TG(18:0e/9:0/9:0)	TG	M+Na	C39 H76 O5 Na1	16.90618958	647.5584965	POS
TG(18:1/10:1/10:1)	TG	M+NH4	C41 H76 O6 N1	16.48109656	678.5667155	POS
TG(18:1/10:1/10:4)	TG	M+H	C41 H67 O6	12.01410061	655.4932165	POS
TG(18:1/10:1/18:1)	TG	M+Na	C49 H88 O6 Na1	18.22166148	795.6473115	POS
TG(18:1/10:1/18:2)	TG	M+NH4	C49 H90 O6 N1	19.09346675	788.6762655	POS

TG(18:1/10:1/23:1)	TG	M+NH4	C54 H102 O6 N1	21.191	860.7701655	POS
TG(18:1/10:1/24:1)	TG	M+NH4	C55 H104 O6 N1	13.30886539	874.7858155	POS
TG(18:1/10:2/13:0)	TG	M+NH4	C44 H82 O6 N1	14.73	720.6136655	POS
TG(18:1/10:2/17:1)	TG	M+H	C48 H85 O6	19.28914391	757.6340665	POS
TG(18:1/10:2/18:1)	TG	M+Na	C49 H86 O6 Na1	15.07401156	793.6316615	POS
TG(18:1/10:3/18:2)	TG	M+H	C49 H83 O6	18.315	767.6184165	POS
TG(18:1/10:4/18:1)	TG	M+H	C49 H83 O6	16.078	767.6184165	POS
TG(18:1/11:1/12:4)	TG	M+NH4	C44 H76 O6 N1	13.59212352	714.5667155	POS
TG(18:1/11:1/18:2)	TG	M+H	C50 H89 O6	17.573	785.6653665	POS
TG(18:1/11:3/12:4)	TG	M+Na	C44 H68 O6 Na1	10.25936221	715.4908115	POS
TG(18:1/11:3/18:1)	TG	M+H	C50 H87 O6	17.47201675	783.6497165	POS
TG(18:1/11:3/18:2)	TG	M+H	C50 H85 O6	15.5962648	781.6340665	POS
TG(18:1/11:4/12:2)	TG	M+Na	C44 H70 O6 Na1	11.28636629	717.5064615	POS
TG(18:1/11:4/18:1)	TG	M+H	C50 H85 O6	14.87467946	781.6340665	POS
TG(18:1/11:4/18:2)	TG	M+H	C50 H83 O6	14.859	779.6184165	POS
TG(18:1/12:0/18:2)	TG	M+NH4	C51 H96 O6 N1	19.97763247	818.7232155	POS
TG(18:1/12:3/18:1)	TG	M+H	C51 H89 O6	18.74227554	797.6653665	POS
TG(18:1/12:4/14:1)	TG	M+NH4	C47 H82 O6 N1	14.86783445	756.6136655	POS
TG(18:1/12:4/18:1)	TG	M+H	C51 H87 O6	18.18436213	795.6497165	POS
TG(18:1/12:4/18:2)	TG	M+H	C51 H85 O6	14.9813416	793.6340665	POS
TG(18:1/13:0/18:1)	TG	M+NH4	C52 H100 O6 N1	20.71871195	834.7545155	POS

TG(18:1/13:0/18:2)	TG	M+NH4	C52 H98 O6 N1	20.24014885	832.7388655	POS
TG(18:1/14:0/14:0)	TG	M+NH4	C49 H96 O6 N1	20.41470013	794.7232155	POS
TG(18:1/14:0/18:2)	TG	M+NH4	C53 H100 O6 N1	24.77424909	846.7545155	POS
TG(18:1/14:0/18:3)	TG	M+H	C53 H95 O6	18.28395874	827.7123165	POS
TG(18:1/14:1/18:1)	TG	M+NH4	C53 H100 O6 N1	7.652	846.7545155	POS
TG(18:1/17:1/18:2)	TG	M+NH4	C56 H104 O6 N1	20.70769006	886.7858155	POS
TG(18:1/18:1/20:2)	TG	M+H	C59 H107 O6	20.183	911.8062165	POS
TG(18:1/18:1/21:0)	TG	M+NH4	C60 H116 O6 N1	22.02803852	946.8797155	POS
TG(18:1/18:1/22:0)	TG	M+H	C61 H115 O6	22.16667191	943.8688165	POS
TG(18:1/18:1/22:1)	TG	M+NH4	C61 H116 O6 N1	21.87167772	958.8797155	POS
TG(18:1/18:1/22:4)	TG	M+H	C61 H107 O6	22.183	935.8062165	POS
TG(18:1/18:1/22:5)	TG	M+H	C61 H105 O6	18.22389093	933.7905665	POS
TG(18:1/18:1/22:6)	TG	M+H	C61 H103 O6	21.778	931.7749165	POS
TG(18:1/18:1/23:0)	TG	M+NH4	C62 H120 O6 N1	21.533	974.9110155	POS
TG(18:1/18:1/24:0)	TG	M+NH4	C63 H122 O6 N1	22.37252771	988.9266655	POS
TG(18:1/18:1/24:1)	TG	M+NH4	C63 H120 O6 N1	25.531	986.9110155	POS
TG(18:1/18:2/20:2)	TG	M+H	C59 H105 O6	24.50029694	909.7905665	POS
TG(18:1/18:2/21:0)	TG	M+NH4	C60 H114 O6 N1	21.739784	944.8640655	POS
TG(18:1/18:2/22:0)	TG	M+NH4	C61 H116 O6 N1	24.07	958.8797155	POS
TG(18:1/18:2/22:1)	TG	M+NH4	C61 H114 O6 N1	21.51524945	956.8640655	POS
TG(18:1/18:2/22:2)	TG	M+Na	C61 H108 O6 Na1	18.57289846	959.8038115	POS

TG(18:1/18:2/22:3)	TG	M+H	C61 H107 O6	19.259060 48	935.80621 65	POS
TG(18:1/18:2/22:4)	TG	M+H	C61 H105 O6	25.642	933.79056 65	POS
TG(18:1/18:2/22:5)	TG	M+H	C61 H103 O6	17.542719 79	931.77491 65	POS
TG(18:1/18:2/22:6)	TG	M+H	C61 H101 O6	18.949426 05	929.75926 65	POS
TG(18:1/18:2/23:0)	TG	M+NH4	C62 H118 O6 N1	22.011916 76	972.89536 55	POS
TG(18:1/18:2/24:0)	TG	M+NH4	C63 H120 O6 N1	22.142842 78	986.91101 55	POS
TG(18:1/18:3/18:3)	TG	M+H	C57 H97 O6	16.588	877.72796 65	POS
TG(18:1/18:3/22:3)	TG	M+H	C61 H105 O6	18.717583 36	933.79056 65	POS
TG(18:1/18:3/22:5)	TG	M+H	C61 H101 O6	17.64	929.75926 65	POS
TG(18:1/20:2/22:3)	TG	M+H	C63 H111 O6	19.919	963.83751 65	POS
TG(18:1/20:5/23:0)	TG	M+H	C64 H113 O6	22.033	977.85316 65	POS
TG(18:1/22:0/22:4)	TG	M+H	C65 H117 O6	20.670671 7	993.88446 65	POS
TG(18:1/22:4/24:2)	TG	M+H	C67 H117 O6	20.193	1017.8844 66	POS
TG(18:1/22:6/24:1)	TG	M+H	C67 H115 O6	20.217311 57	1015.8688 16	POS
TG(18:1/24:0/24:0)	TG	M+NH4	C69 H136 O6 N1	23.253806 58	1075.0362 15	POS
TG(18:1e/10:2/10:2)	TG	M+H	C41 H71 O5	13.406	643.52960 15	POS
TG(18:1e/10:3/18:1)	TG	M+Na	C49 H86 O5 Na1	18.91	777.63674 65	POS
TG(18:1e/12:3/12:3)	TG	M+H	C45 H75 O5	13.978812 64	695.56090 15	POS
TG(18:1e/12:4/14:4)	TG	M+H	C47 H75 O5	11.333	719.56090 15	POS
TG(18:1e/18:2/18:3)	TG	M+Na	C57 H100 O5 Na1	18.058	887.74629 65	POS
TG(18:1e/20:0/22:6)	TG	M+H	C63 H111 O5	22.133	947.84260 15	POS

TG(18:1e/6:0/10:1)	TG	M+Na	C37 H68 O5 Na1	15.707562 43	615.49589 65	POS
TG(18:1e/6:0/10:2)	TG	M+Na	C37 H66 O5 Na1	10.803847 42	613.48024 65	POS
TG(18:1e/6:0/11:0)	TG	M+NH4	C38 H76 O5 N1	16.087	626.57180 05	POS
TG(18:2/10:1/10:1)	TG	M+NH4	C41 H74 O6 N1	15.398918 49	676.55106 55	POS
TG(18:2/10:1/10:4)	TG	M+H	C41 H65 O6	10.693425 15	653.47756 65	POS
TG(18:2/10:1/18:2)	TG	M+Na	C49 H84 O6 Na1	14.139	791.61601 15	POS
TG(18:2/10:2/10:3)	TG	M+H	C41 H65 O6	10.408	653.47756 65	POS
TG(18:2/10:2/21:0)	TG	M+NH4	C52 H96 O6 N1	17.617	830.72321 55	POS
TG(18:2/10:3/10:4)	TG	M+NH4	C41 H64 O6 N1	12.285	666.47281 55	POS
TG(18:2/10:3/18:2)	TG	M+H	C49 H81 O6	14.91	765.60276 65	POS
TG(18:2/11:2/18:2)	TG	M+H	C50 H85 O6	16.712270 11	781.63406 65	POS
TG(18:2/11:3/12:2)	TG	M+Na	C44 H70 O6 Na1	9.121	717.50646 15	POS
TG(18:2/11:3/18:2)	TG	M+H	C50 H83 O6	13.994	779.61841 65	POS
TG(18:2/11:4/12:2)	TG	M+Na	C44 H68 O6 Na1	8.0700551 36	715.49081 15	POS
TG(18:2/12:3/18:2)	TG	M+H	C51 H85 O6	14.496	793.63406 65	POS
TG(18:2/12:4/18:2)	TG	M+H	C51 H83 O6	14.145616 83	791.61841 65	POS
TG(18:2/14:1/18:2)	TG	M+NH4	C53 H96 O6 N1	19.627141 53	842.72321 55	POS
TG(18:2/17:1/18:2)	TG	M+NH4	C56 H102 O6 N1	20.280613 59	884.77016 55	POS
TG(18:2/18:2/21:0)	TG	M+NH4	C60 H112 O6 N1	21.454265 54	942.84841 55	POS
TG(18:2/18:2/22:6)	TG	M+H	C61 H99 O6	16.983	927.74361 65	POS
TG(18:2/18:2/23:0)	TG	M+NH4	C62 H116 O6 N1	21.759920 41	970.87971 55	POS

TG(18:2/18:2/23:1)	TG	M+NH4	C62 H114 O6 N1	21.48341619	968.8640655	POS
TG(18:2e/6:0/10:1)	TG	M+Na	C37 H66 O5 Na1	6.752	613.4802465	POS
TG(18:2e/6:0/10:2)	TG	M+H	C37 H65 O5	18.373	589.4826515	POS
TG(18:2e/6:0/10:3)	TG	M+H	C37 H63 O5	18.487	587.4670015	POS
TG(18:2e/6:0/11:3)	TG	M+H	C38 H65 O5	9.87453969	601.4826515	POS
TG(18:2e/6:0/11:4)	TG	M+H	C38 H63 O5	8.679	599.4670015	POS
TG(18:2e/6:0/9:0)	TG	M+H	C36 H67 O5	14.01500244	579.4983015	POS
TG(18:3/10:1/11:3)	TG	M+Na	C42 H66 O6 Na1	10.703	689.4751615	POS
TG(18:3/10:2/10:4)	TG	M+NH4	C41 H64 O6 N1	13.71361071	666.4728155	POS
TG(18:3/10:2/11:1)	TG	M+Na	C42 H68 O6 Na1	12.48459746	691.4908115	POS
TG(18:3/10:2/23:1)	TG	M+Na	C54 H92 O6 Na1	17.666	859.6786115	POS
TG(18:3/10:4/22:6)	TG	M+NH4	C53 H80 O6 N1	8.13	826.5980155	POS
TG(18:3/11:3/12:2)	TG	M+Na	C44 H68 O6 Na1	10.558	715.4908115	POS
TG(18:3/11:3/12:3)	TG	M+Na	C44 H66 O6 Na1	9.244065077	713.4751615	POS
TG(18:3/12:2/21:0)	TG	M+NH4	C54 H98 O6 N1	17.058	856.7388655	POS
TG(18:3/17:1/18:2)	TG	M+NH4	C56 H100 O6 N1	19.873	882.7545155	POS
TG(18:3/17:1/18:3)	TG	M+Na	C56 H94 O6 Na1	19.753	885.6942615	POS
TG(18:3/17:1/20:5)	TG	M+NH4	C58 H98 O6 N1	20.52474142	904.7388655	POS
TG(18:3/17:1/21:1)	TG	M+Na	C59 H104 O6 Na1	16.57890248	931.7725115	POS
TG(18:3/18:2/18:3)	TG	M+H	C57 H95 O6	17.08543977	875.7123165	POS
TG(18:3/18:2/20:3)	TG	M+H	C59 H99 O6	17.065	903.7436165	POS

TG(18:3/18:2/22:1)	TG	M+NH4	C61 H110 O6 N1	20.99300319	952.8327655	POS
TG(18:3/18:2/22:3)	TG	M+H	C61 H103 O6	16.40059618	931.7749165	POS
TG(18:3/18:2/22:4)	TG	M+H	C61 H101 O6	18.49229428	929.7592665	POS
TG(18:3/18:2/24:2)	TG	M+H	C63 H109 O6	24.762	961.8218665	POS
TG(18:3/18:3/18:3)	TG	M+H	C57 H93 O6	13.6349982	873.6966665	POS
TG(18:3/18:3/22:1)	TG	M+H	C61 H105 O6	17.028	933.7905665	POS
TG(18:3/18:3/22:3)	TG	M+H	C61 H101 O6	14.227	929.7592665	POS
TG(18:3/18:3/22:4)	TG	M+Na	C61 H98 O6 Na1	14.465	949.7255615	POS
TG(18:3/18:3/23:1)	TG	M+H	C62 H107 O6	19.064	947.8062165	POS
TG(18:3/18:3/24:2)	TG	M+Na	C63 H106 O6 Na1	13.049	981.7881615	POS
TG(18:3e/18:3/18:3)	TG	M+H	C57 H95 O5	19.5891003	859.7174015	POS
TG(18:3e/6:0/10:2)	TG	M+H	C37 H63 O5	10.702	587.4670015	POS
TG(18:3e/6:0/12:2)	TG	M+H	C39 H67 O5	13.59313733	615.4983015	POS
TG(18:3e/8:0/10:1)	TG	M+H	C39 H69 O5	16.73825781	617.5139515	POS
TG(18:3e/9:0/9:0)	TG	M+H	C39 H71 O5	18.567	619.5296015	POS
TG(18:4/10:2/16:0)	TG	M+NH4	C47 H82 O6 N1	13.60729589	756.6136655	POS
TG(18:4/11:1/16:0)	TG	M+H	C48 H83 O6	12.73801841	755.6184165	POS
TG(18:4/11:3/23:0)	TG	M+H	C55 H93 O6	17.467	849.6966665	POS
TG(18:4/12:1/16:0)	TG	M+H	C49 H85 O6	18.17606288	769.6340665	POS
TG(18:4/16:0/16:0)	TG	M+H	C53 H95 O6	18.58156152	827.7123165	POS
TG(18:4/16:0/18:1)	TG	M+H	C55 H97 O6	19.01256023	853.7279665	POS

TG(18:4/16:0/18:2)	TG	M+H	C55 H95 O6	17.437	851.7123165	POS
TG(18:4/16:0/18:3)	TG	M+H	C55 H93 O6	16.567	849.6966665	POS
TG(18:4/18:0/18:0)	TG	M+H	C57 H103 O6	19.739852	883.7749165	POS
TG(18:4/18:1/18:2)	TG	M+H	C57 H97 O6	16.30461345	877.7279665	POS
TG(18:4/18:1/18:3)	TG	M+H	C57 H95 O6	11.63	875.7123165	POS
TG(18:4/18:1/20:0)	TG	M+Na	C59 H104 O6 Na1	22.326	931.7725115	POS
TG(18:4/18:2/18:2)	TG	M+H	C57 H95 O6	14.94741206	875.7123165	POS
TG(18:4/18:2/18:3)	TG	M+H	C57 H93 O6	14.132	873.6966665	POS
TG(18:4/18:2/24:2)	TG	M+H	C63 H107 O6	18.56891117	959.8062165	POS
TG(18:4/18:3/22:4)	TG	M+H	C61 H97 O6	15.224	925.7279665	POS
TG(18:4/19:0/19:0)	TG	M+NH4	C59 H110 O6 N1	20.821	928.8327655	POS
TG(18:4/6:0/12:2)	TG	M+H	C39 H63 O6	6.923	627.4619165	POS
TG(18:4/6:0/6:0)	TG	M+H	C33 H55 O6	10.40486848	547.3993165	POS
TG(18:4/8:0/10:2)	TG	M+H	C39 H63 O6	14.841	627.4619165	POS
TG(18:4/9:0/18:1)	TG	M+H	C48 H83 O6	18.70026662	755.6184165	POS
TG(18:4/9:0/18:2)	TG	M+H	C48 H81 O6	18.18041687	753.6027665	POS
TG(18:4/9:0/18:3)	TG	M+H	C48 H79 O6	14.532	751.5871165	POS
TG(19:0/12:4/12:4)	TG	M+NH4	C46 H76 O6 N1	13.55716523	738.5667155	POS
TG(19:0/18:1/18:1)	TG	M+NH4	C58 H112 O6 N1	21.75148853	918.8484155	POS
TG(19:0/18:1/21:1)	TG	M+NH4	C61 H118 O6 N1	24.44635879	960.8953655	POS
TG(19:0/18:2/19:0)	TG	M+NH4	C59 H114 O6 N1	24.946	932.8640655	POS

TG(19:0/18:3/19:0)	TG	M+NH4	C59 H112 O6 N1	24.4003564	930.8484155	POS
TG(19:0/6:0/12:1)	TG	M+NH4	C40 H78 O6 N1	14.67	668.5823655	POS
TG(19:0/6:0/6:0)	TG	M+NH4	C34 H68 O6 N1	10.87975923	586.5041155	POS
TG(19:0/6:0/9:0)	TG	M+Na	C37 H70 O6 Na1	8.746	633.5064615	POS
TG(19:1/10:3/15:0)	TG	M+H	C47 H83 O6	13.041	743.6184165	POS
TG(19:1/12:3/12:4)	TG	M+H	C46 H73 O6	12.054	721.5401665	POS
TG(19:1/18:1/18:2)	TG	M+NH4	C58 H108 O6 N1	21.0895039	914.8171155	POS
TG(19:1/18:1/19:1)	TG	M+NH4	C59 H112 O6 N1	25.63562418	930.8484155	POS
TG(19:1/18:2/18:2)	TG	M+NH4	C58 H106 O6 N1	20.69394778	912.8014655	POS
TG(19:1/18:2/18:3)	TG	M+NH4	C58 H104 O6 N1	20.33617335	910.7858155	POS
TG(19:1/18:2/19:1)	TG	M+NH4	C59 H110 O6 N1	26.052	928.8327655	POS
TG(19:1/19:1/22:2)	TG	M+NH4	C63 H118 O6 N1	25.00610417	984.8953655	POS
TG(19:1/6:0/18:1)	TG	M+Na	C46 H84 O6 Na1	17.14056927	755.6160115	POS
TG(19:1/6:0/18:2)	TG	M+Na	C46 H82 O6 Na1	17.798	753.6003615	POS
TG(19:1/6:0/6:0)	TG	M+NH4	C34 H66 O6 N1	9.247445721	584.4884655	POS
TG(20:0/11:3/14:3)	TG	M+NH4	C48 H84 O6 N1	12.82539102	770.6293155	POS
TG(20:0/11:4/12:3)	TG	M+H	C46 H75 O6	14.47366274	723.5558165	POS
TG(20:0/11:4/12:4)	TG	M+NH4	C46 H76 O6 N1	13.22426183	738.5667155	POS
TG(20:0/14:1/20:0)	TG	M+Na	C57 H108 O6 Na1	21.275	911.8038115	POS
TG(20:0/16:0/16:0)	TG	M+NH4	C55 H110 O6 N1	21.93279255	880.8327655	POS
TG(20:0/16:0/18:1)	TG	M+NH4	C57 H112 O6 N1	21.89342899	906.8484155	POS

TG(20:0/18:1/18:2)	TG	M+NH4	C59 H112 O6 N1	26.19835379	930.8484155	POS
TG(20:0/18:1/18:3)	TG	M+Na	C59 H106 O6 Na1	19.75534266	933.7881615	POS
TG(20:0/18:1/20:5)	TG	M+H	C61 H107 O6	26.341	935.8062165	POS
TG(20:0/18:2/18:2)	TG	M+NH4	C59 H110 O6 N1	21.24749997	928.8327655	POS
TG(20:0/18:2/22:5)	TG	M+H	C63 H109 O6	19.49012096	961.8218665	POS
TG(20:0/18:2/22:6)	TG	M+NH4	C63 H110 O6 N1	18.87498629	976.8327655	POS
TG(20:0/6:0/12:2)	TG	M+H	C41 H75 O6	13.261	663.5558165	POS
TG(20:0/8:0/10:1)	TG	M+NH4	C41 H80 O6 N1	15.33386977	682.5980155	POS
TG(20:0/8:0/12:4)	TG	M+H	C43 H75 O6	15.33740203	687.5558165	POS
TG(20:0/9:0/9:0)	TG	M+NH4	C41 H82 O6 N1	16.27554752	684.6136655	POS
TG(20:0e/10:3/14:2)	TG	M+H	C47 H83 O5	13.89678324	727.6235015	POS
TG(20:0e/11:3/11:3)	TG	M+Na	C45 H76 O5 Na1	10.168	719.5584965	POS
TG(20:0e/11:4/11:4)	TG	M+NH4	C45 H76 O5 N1	15.59153701	710.5718005	POS
TG(20:0e/11:4/12:3)	TG	M+NH4	C46 H80 O5 N1	15.771	726.6031005	POS
TG(20:0e/11:4/12:4)	TG	M+NH4	C46 H78 O5 N1	15.70390796	724.5874505	POS
TG(20:0e/12:2/12:3)	TG	M+NH4	C47 H86 O5 N1	17.56969884	744.6500505	POS
TG(20:0e/12:3/12:3)	TG	M+NH4	C47 H84 O5 N1	16.832	742.6344005	POS
TG(20:0e/12:4/12:4)	TG	M+H	C47 H77 O5	11.872	721.5765515	POS
TG(20:0e/14:3/18:2)	TG	M+H	C55 H99 O5	19.43320335	839.7487015	POS
TG(20:0e/16:0/18:1)	TG	M+NH4	C57 H114 O5 N1	22.11225585	892.8691505	POS
TG(20:0e/16:0/18:2)	TG	M+Na	C57 H108 O5 Na1	19.482	895.8088965	POS

TG(20:0e/18:1/18:2)	TG	M+NH4	C59 H114 O5 N1	22.01994695	916.8691505	POS
TG(20:0e/18:1/18:3)	TG	M+Na	C59 H108 O5 Na1	19.99	919.8088965	POS
TG(20:0e/18:3/18:3)	TG	M+Na	C59 H104 O5 Na1	18.49377812	915.7775965	POS
TG(20:0e/6:0/11:4)	TG	M+NH4	C40 H74 O5 N1	14.07017798	648.5561505	POS
TG(20:0e/6:0/12:3)	TG	M+H	C41 H75 O5	13.694	647.5609015	POS
TG(20:1/11:2/22:6)	TG	M+H	C56 H91 O6	17.669	859.6810165	POS
TG(20:1/14:0/14:0)	TG	M+NH4	C51 H100 O6 N1	24.4955858	822.7545155	POS
TG(20:1/14:2/20:1)	TG	M+H	C57 H103 O6	17.738	883.7749165	POS
TG(20:1/17:1/18:1)	TG	M+NH4	C58 H110 O6 N1	20.249	916.8327655	POS
TG(20:1/18:1/18:1)	TG	M+NH4	C59 H112 O6 N1	21.51273276	930.8484155	POS
TG(20:1/18:1/18:2)	TG	M+NH4	C59 H110 O6 N1	22.253	928.8327655	POS
TG(20:1/18:1/18:3)	TG	M+H	C59 H105 O6	20.18580863	909.7905665	POS
TG(20:1/18:1/22:4)	TG	M+NH4	C63 H114 O6 N1	17.558	980.8640655	POS
TG(20:1/18:1/24:2)	TG	M+Na	C65 H118 O6 Na1	20.202	1017.882061	POS
TG(20:1/18:2/18:2)	TG	M+NH4	C59 H108 O6 N1	21.00411839	926.8171155	POS
TG(20:1/6:0/11:2)	TG	M+NH4	C40 H74 O6 N1	14.13311648	664.5510655	POS
TG(20:1/6:0/12:1)	TG	M+NH4	C41 H78 O6 N1	14.04540083	680.5823655	POS
TG(20:1/6:0/12:2)	TG	M+H	C41 H73 O6	13.395	661.5401665	POS
TG(20:1/6:0/14:4)	TG	M+H	C43 H73 O6	14.01492219	685.5401665	POS
TG(20:1/6:0/8:0)	TG	M+Na	C37 H68 O6 Na1	18.373	631.4908115	POS
TG(20:1/8:0/10:2)	TG	M+H	C41 H73 O6	13.0722908	661.5401665	POS

TG(20:1e/10:2/12:2)	TG	M+Na	C45 H78 O5 Na1	13.18020767	721.5741465	POS
TG(20:1e/10:3/12:2)	TG	M+Na	C45 H76 O5 Na1	11.068	719.5584965	POS
TG(20:1e/11:4/12:4)	TG	M+NH4	C46 H76 O5 N1	15.29	722.5718005	POS
TG(20:1e/12:3/18:1)	TG	M+NH4	C53 H98 O5 N1	20.348	828.7439505	POS
TG(20:1e/16:0/18:2)	TG	M+Na	C57 H106 O5 Na1	18.924	893.7932465	POS
TG(20:1e/18:2/18:3)	TG	M+Na	C59 H104 O5 Na1	17.887	915.7775965	POS
TG(20:1e/18:3/18:3)	TG	M+H	C59 H103 O5	18.99309606	891.7800015	POS
TG(20:1e/6:0/11:3)	TG	M+H	C40 H71 O5	15.31	631.5296015	POS
TG(20:1e/9:0/9:0)	TG	M+H	C41 H79 O5	16.103	651.5922015	POS
TG(20:2)	TG	M+H	C23 H39 O6	7.567	411.2741165	POS
TG(20:2/11:3/12:1)	TG	M+NH4	C46 H80 O6 N1	12.366	742.5980155	POS
TG(20:2/18:2/18:2)	TG	M+H	C59 H103 O6	18.14740398	907.7749165	POS
TG(20:2e/10:1/12:4)	TG	M+H	C45 H75 O5	17.255	695.5609015	POS
TG(20:2e/11:3/11:3)	TG	M+NH4	C45 H76 O5 N1	9.063	710.5718005	POS
TG(20:2e/12:3/12:3)	TG	M+H	C47 H77 O5	12.136	721.5765515	POS
TG(20:2e/16:1/18:2)	TG	M+H	C57 H103 O5	20.01024876	867.7800015	POS
TG(20:2e/18:1/18:2)	TG	M+Na	C59 H106 O5 Na1	18.45993083	917.7932465	POS
TG(20:2e/18:1/24:2)	TG	M+Na	C65 H118 O5 Na1	20.7397575	1001.887146	POS
TG(20:2e/18:2/18:2)	TG	M+H	C59 H105 O5	20.01066289	893.7956515	POS
TG(20:2e/18:2/20:2)	TG	M+H	C61 H109 O5	20.41857543	921.8269515	POS
TG(20:2e/18:3/18:3)	TG	M+H	C59 H101 O5	18.47864035	889.7643515	POS

TG(20:2e/6:0/10:3)	TG	M+H	C39 H67 O5	21.99	615.49830 15	POS
TG(20:2e/6:0/6:0)	TG	M+Na	C35 H64 O5 Na1	14.412	587.46459 65	POS
TG(20:2e/6:0/8:0)	TG	M+Na	C37 H68 O5 Na1	18.328768 79	615.49589 65	POS
TG(20:3e/10:2/12:4)	TG	M+NH4	C45 H74 O5 N1	14.51	708.55615 05	POS
TG(20:3e/10:3/14:3)	TG	M+H	C47 H75 O5	13.042175 08	719.56090 15	POS
TG(20:3e/11:3/18:1)	TG	M+H	C52 H89 O5	16.528	793.67045 15	POS
TG(20:3e/14:0/18:3)	TG	M+Na	C55 H96 O5 Na1	17.775	859.71499 65	POS
TG(20:3e/16:0/18:3)	TG	M+H	C57 H101 O5	19.662911 4	865.76435 15	POS
TG(20:3e/16:0/18:4)	TG	M+H	C57 H99 O5	18.561	863.74870 15	POS
TG(20:3e/18:0/18:2)	TG	M+H	C59 H107 O5	20.432	895.81130 15	POS
TG(20:3e/18:1/18:2)	TG	M+H	C59 H105 O5	20.983	893.79565 15	POS
TG(20:3e/18:2/18:2)	TG	M+H	C59 H103 O5	19.585289 33	891.78000 15	POS
TG(20:3e/18:2/18:3)	TG	M+H	C59 H101 O5	19.149470 74	889.76435 15	POS
TG(20:3e/6:0/10:0)	TG	M+H	C39 H71 O5	21.445769 13	619.52960 15	POS
TG(20:3e/6:0/10:1)	TG	M+H	C39 H69 O5	21.836	617.51395 15	POS
TG(20:3e/6:0/10:2)	TG	M+H	C39 H67 O5	20.269	615.49830 15	POS
TG(20:3e/6:0/11:2)	TG	M+H	C40 H69 O5	15.434	629.51395 15	POS
TG(20:3e/6:0/9:0)	TG	M+NH4	C38 H72 O5 N1	13.383	622.54050 05	POS
TG(20:3e/8:0/10:0)	TG	M+H	C41 H75 O5	15.041	647.56090 15	POS
TG(20:4/17:1/17:1)	TG	M+NH4	C57 H102 O6 N1	4.693	896.77016 55	POS
TG(20:4e/10:3/18:1)	TG	M+NH4	C51 H88 O5 N1	16.71	794.66570 05	POS

TG(20:4e/11:3/18:3)	TG	M+H	C52 H83 O5	15.667	787.62350 15	POS
TG(20:4e/18:2/18:3)	TG	M+H	C59 H99 O5	18.568320 85	887.74870 15	POS
TG(20:4e/18:3/18:3)	TG	M+H	C59 H97 O5	18.095	885.73305 15	POS
TG(20:4e/6:0/10:1)	TG	M+H	C39 H67 O5	8.7365062 68	615.49830 15	POS
TG(20:4e/6:0/10:2)	TG	M+H	C39 H65 O5	14.744	613.48265 15	POS
TG(20:4e/6:0/10:3)	TG	M+H	C39 H63 O5	15.064	611.46700 15	POS
TG(20:4e/6:0/11:3)	TG	M+H	C40 H65 O5	14.795386 86	625.48265 15	POS
TG(20:4e/6:0/16:0)	TG	M+H	C45 H81 O5	18.515894 63	701.60785 15	POS
TG(20:4e/6:0/18:3)	TG	M+NH4	C47 H82 O5 N1	14.774038 93	740.61875 05	POS
TG(20:4e/9:0/12:4)	TG	M+Na	C44 H70 O5 Na1	16.155	701.51154 65	POS
TG(20:4e/9:0/18:3)	TG	M+Na	C50 H84 O5 Na1	9.894	787.62109 65	POS
TG(20:5/10:2/11:2)	TG	M+Na	C44 H66 O6 Na1	9.727	713.47516 15	POS
TG(21:0/10:2/11:3)	TG	M+Na	C45 H76 O6 Na1	10.723	735.55341 15	POS
TG(21:0/10:3/10:3)	TG	M+Na	C44 H72 O6 Na1	9.766	719.52211 15	POS
TG(21:0/11:3/11:3)	TG	M+NH4	C46 H80 O6 N1	14.227308 1	742.59801 55	POS
TG(21:0/11:3/11:4)	TG	M+H	C46 H75 O6	12.419763 19	723.55581 65	POS
TG(21:0/11:3/12:4)	TG	M+NH4	C47 H80 O6 N1	11.610945 87	754.59801 55	POS
TG(21:1/10:2/12:4)	TG	M+NH4	C46 H78 O6 N1	10.117106 96	740.58236 55	POS
TG(21:1/10:4/12:2)	TG	M+H	C46 H75 O6	13.852221 11	723.55581 65	POS
TG(21:1/11:3/11:3)	TG	M+NH4	C46 H78 O6 N1	13.331624 41	740.58236 55	POS
TG(21:3)	TG	M+H	C24 H39 O6	5.799	423.27411 65	POS

TG(22:0/10:2/12:2)	TG	M+NH4	C47 H86 O6 N1	16.38664035	760.6449655	POS
TG(22:0/10:3/10:3)	TG	M+Na	C45 H74 O6 Na1	9.136	733.5377615	POS
TG(22:0/10:3/11:4)	TG	M+H	C46 H75 O6	10.57029033	723.5558165	POS
TG(22:0/10:4/12:4)	TG	M+NH4	C47 H78 O6 N1	13.50267846	752.5823655	POS
TG(22:0/11:3/11:4)	TG	M+NH4	C47 H80 O6 N1	10.1814875	754.5980155	POS
TG(22:0/12:2/18:3)	TG	M+NH4	C55 H100 O6 N1	6.548	870.7545155	POS
TG(22:0/14:2/22:0)	TG	M+NH4	C61 H118 O6 N1	25.63000404	960.8953655	POS
TG(22:0/14:3/22:0)	TG	M+NH4	C61 H116 O6 N1	25.806	958.8797155	POS
TG(22:0/14:4/22:0)	TG	M+H	C61 H111 O6	20.20673062	939.8375165	POS
TG(22:0/18:2/18:2)	TG	M+NH4	C61 H114 O6 N1	21.854	956.8640655	POS
TG(22:0/18:2/22:6)	TG	M+NH4	C65 H114 O6 N1	18.952	1004.864065	POS
TG(22:0/18:3/18:3)	TG	M+Na	C61 H106 O6 Na1	18.527	957.7881615	POS
TG(22:0/22:0/24:0)	TG	M+NH4	C71 H142 O6 N1	23.63354413	1105.083165	POS
TG(22:0/24:0/24:0)	TG	M+NH4	C73 H146 O6 N1	23.801	1133.114465	POS
TG(22:1/10:2/12:4)	TG	M+NH4	C47 H80 O6 N1	13.801	754.5980155	POS
TG(22:1/11:2/11:3)	TG	M+NH4	C47 H82 O6 N1	10.96983061	756.6136655	POS
TG(22:1/18:2/18:2)	TG	M+NH4	C61 H112 O6 N1	21.34619286	954.8484155	POS
TG(22:2/14:1/22:2)	TG	M+Na	C61 H108 O6 Na1	24.025	959.8038115	POS
TG(22:2/18:2/18:2)	TG	M+Na	C61 H106 O6 Na1	17.64113243	957.7881615	POS
TG(22:3/10:2/11:3)	TG	M+H	C46 H73 O6	11.303	721.5401665	POS
TG(22:3/12:4/21:1)	TG	M+H	C58 H97 O6	22.872351	889.7279665	POS

TG(22:3/18:2/18:2)	TG	M+H	C61 H105 O6	16.66	933.7905665	POS
TG(22:5/18:2/18:2)	TG	M+H	C61 H101 O6	16.96671603	929.7592665	POS
TG(22:6/10:3/11:3)	TG	M+NH4	C46 H68 O6 N1	7.642555053	730.5041155	POS
TG(22:6/11:3/12:4)	TG	M+NH4	C48 H70 O6 N1	9.757	756.5197655	POS
TG(23:0)	TG	M+NH4	C26 H52 O6 N1	6.986865894	474.3789155	POS
TG(23:0/10:1/10:1)	TG	M+NH4	C46 H88 O6 N1	17.761026	750.6606155	POS
TG(23:0/10:3/10:3)	TG	M+NH4	C46 H80 O6 N1	13.057	742.5980155	POS
TG(23:0/10:3/11:2)	TG	M+H	C47 H81 O6	10.71828618	741.6027665	POS
TG(23:0/23:0/23:0)	TG	M+NH4	C72 H144 O6 N1	23.691	1119.098815	POS
TG(23:1/10:2/10:2)	TG	M+NH4	C46 H82 O6 N1	11.93879576	744.6136655	POS
TG(23:1/10:2/10:3)	TG	M+NH4	C46 H80 O6 N1	10.97029642	742.5980155	POS
TG(23:1/10:2/11:4)	TG	M+NH4	C47 H80 O6 N1	14.447	754.5980155	POS
TG(23:1/10:3/10:4)	TG	M+H	C46 H73 O6	9.989684533	721.5401665	POS
TG(24:0)	TG	M+NH4	C27 H54 O6 N1	24.677	488.3945655	POS
TG(24:0/10:2/18:3)	TG	M+NH4	C55 H100 O6 N1	21.067	870.7545155	POS
TG(24:0/12:2/24:0)	TG	M+NH4	C63 H122 O6 N1	26.29855589	988.9266655	POS
TG(24:0/12:3/24:0)	TG	M+H	C63 H117 O6	21.134	969.8844665	POS
TG(24:0/12:4/23:1)	TG	M+NH4	C62 H114 O6 N1	21.926	968.8640655	POS
TG(24:0/12:4/24:0)	TG	M+NH4	C63 H118 O6 N1	24.604	984.8953655	POS
TG(24:0/14:3/14:3)	TG	M+Na	C55 H94 O6 Na1	16.735	873.6942615	POS
TG(24:0/18:2/18:2)	TG	M+NH4	C63 H118 O6 N1	21.90527462	984.8953655	POS

TG(24:0/18:2/18:3)	TG	M+H	C63 H113 O6	20.255	965.8531665	POS
TG(24:0/18:2/22:3)	TG	M+H	C67 H121 O6	21.02729575	1021.915766	POS
TG(24:0/18:2/22:5)	TG	M+H	C67 H117 O6	20.56943409	1017.884466	POS
TG(24:0/18:3/18:3)	TG	M+NH4	C63 H114 O6 N1	21.41336631	980.8640655	POS
TG(24:1/10:3/11:2)	TG	M+H	C48 H81 O6	12.20522732	753.6027665	POS
TG(24:1/10:3/11:3)	TG	M+NH4	C48 H82 O6 N1	11.70374791	768.6136655	POS
TG(24:1/12:2/24:1)	TG	M+NH4	C63 H118 O6 N1	22.534	984.8953655	POS
TG(24:1/18:2/18:2)	TG	M+NH4	C63 H116 O6 N1	21.53007299	982.8797155	POS
TG(24:1/18:3/18:3)	TG	M+H	C63 H109 O6	19.09995726	961.8218665	POS
TG(24:2/10:2/10:2)	TG	M+H	C47 H79 O6	11.7857403	739.5871165	POS
TG(24:2/10:2/11:2)	TG	M+NH4	C48 H84 O6 N1	11.411	770.6293155	POS
TG(24:2/10:3/10:3)	TG	M+H	C47 H75 O6	10.876	735.5558165	POS
TG(24:2/18:2/18:2)	TG	M+H	C63 H111 O6	19.58188766	963.8375165	POS
TG(25:0/11:2/18:3)	TG	M+H	C57 H101 O6	8.11	881.7592665	POS
TG(25:0/16:0/16:0)	TG	M+NH4	C60 H120 O6 N1	22.553	950.9110155	POS
TG(25:0/16:0/18:1)	TG	M+NH4	C62 H122 O6 N1	22.53137174	976.9266655	POS
TG(25:0/16:0/24:0)	TG	M+NH4	C68 H136 O6 N1	23.36748162	1063.036215	POS
TG(25:0/16:1/20:2)	TG	M+Na	C64 H118 O6 Na1	20.641	1005.882061	POS
TG(25:0/18:1/18:1)	TG	M+NH4	C64 H124 O6 N1	22.50892653	1002.942315	POS
TG(25:0/18:1/18:3)	TG	M+Na	C64 H116 O6 Na1	20.11718237	1003.866411	POS
TG(25:0/18:1/22:5)	TG	M+H	C68 H121 O6	21.094	1033.915766	POS

TG(25:0/18:1/24:0)	TG	M+NH4	C70 H138 O6 N1	23.34028165	1089.051865	POS
TG(25:0/18:2/18:2)	TG	M+NH4	C64 H120 O6 N1	22.02	998.9110155	POS
TG(25:0/18:2/18:3)	TG	M+NH4	C64 H118 O6 N1	21.69247263	996.8953655	POS
TG(25:0/18:2/22:5)	TG	M+H	C68 H119 O6	20.65399663	1031.900116	POS
TG(25:0/20:0/22:0)	TG	M+NH4	C70 H140 O6 N1	23.536	1091.067515	POS
TG(25:0/6:0/10:2)	TG	M+NH4	C44 H84 O6 N1	16.095	722.6293155	POS
TG(25:0/6:0/18:1)	TG	M+NH4	C52 H102 O6 N1	19.8358398	836.7701655	POS
TG(25:1)	TG	M+NH4	C28 H54 O6 N1	4.87	500.3945655	POS
TG(25:1/18:1/18:1)	TG	M+NH4	C64 H122 O6 N1	22.26549021	1000.926665	POS
TG(25:1/18:1/24:1)	TG	M+NH4	C70 H134 O6 N1	22.331	1085.020565	POS
TG(25:1/18:2/24:2)	TG	M+NH4	C70 H130 O6 N1	22.47342817	1080.989265	POS
TG(25:1/6:0/11:2)	TG	M+Na	C45 H80 O6 Na1	11.718	739.5847115	POS
TG(25:1/6:0/16:0)	TG	M+NH4	C50 H98 O6 N1	19.52977347	808.7388655	POS
TG(25:2)	TG	M+NH4	C28 H52 O6 N1	7.536	498.3789155	POS
TG(25:3)	TG	M+H	C28 H47 O6	4.857	479.3367165	POS
TG(26:0)	TG	M+H	C29 H55 O6	8.624227146	499.3993165	POS
TG(26:0/11:3/11:3)	TG	M+H	C51 H87 O6	16.408	795.6497165	POS
TG(26:0/14:0/22:4)	TG	M+H	C65 H119 O6	21.00217063	995.9001165	POS
TG(26:0/16:0/18:1)	TG	M+NH4	C63 H124 O6 N1	22.64347357	990.9423155	POS
TG(26:0/16:1/16:1)	TG	M+NH4	C61 H118 O6 N1	26.32630279	960.8953655	POS
TG(26:0/18:1/18:1)	TG	M+NH4	C65 H126 O6 N1	22.61180703	1016.957965	POS

TG(26:0/18:1/18:2)	TG	M+NH4	C65 H124 O6 N1	22.716	1014.942315	POS
TG(26:0/18:1/22:6)	TG	M+H	C69 H121 O6	20.7740174	1045.915766	POS
TG(26:0/18:1/24:0)	TG	M+NH4	C71 H140 O6 N1	23.42	1103.067515	POS
TG(26:0/18:1/24:1)	TG	M+NH4	C71 H138 O6 N1	23.23356343	1101.051865	POS
TG(26:0/18:1/24:2)	TG	M+NH4	C71 H136 O6 N1	23.00849601	1099.036215	POS
TG(26:0/18:2/18:3)	TG	M+Na	C65 H116 O6 Na1	19.64	1015.866411	POS
TG(26:0/19:0/24:0)	TG	M+H	C72 H141 O6	23.227	1102.072266	POS
TG(26:1)	TG	M+NH4	C29 H56 O6 N1	7.826623399	514.4102155	POS
TG(26:1/12:2/18:4)	TG	M+Na	C59 H100 O6 Na1	14.12	927.7412115	POS
TG(26:1/18:1/18:1)	TG	M+NH4	C65 H124 O6 N1	22.40085871	1014.942315	POS
TG(26:1/18:1/18:2)	TG	M+NH4	C65 H122 O6 N1	22.1716585	1012.926665	POS
TG(26:1/18:1/23:0)	TG	M+NH4	C70 H136 O6 N1	23.14987635	1087.036215	POS
TG(26:1/18:1/23:1)	TG	M+NH4	C70 H134 O6 N1	22.90924062	1085.020565	POS
TG(26:1/18:2/18:2)	TG	M+NH4	C65 H120 O6 N1	21.82296801	1010.911015	POS
TG(26:1/18:2/24:2)	TG	M+NH4	C71 H132 O6 N1	22.57263903	1095.004915	POS
TG(26:1/18:3/18:3)	TG	M+NH4	C65 H116 O6 N1	19.70295641	1006.879715	POS
TG(26:1/8:0/8:0)	TG	M+NH4	C45 H88 O6 N1	24.695	738.6606155	POS
TG(26:2)	TG	M+H	C29 H51 O6	6.706	495.3680165	POS
TG(26:3)	TG	M+H	C29 H49 O6	5.159	493.3523665	POS
TG(26:4)	TG	M+H	C29 H47 O6	5.028	491.3367165	POS
TG(27:0)	TG	M+NH4	C30 H60 O6 N1	8.93	530.4415155	POS

TG(27:0/16:0/16:0)	TG	M+NH4	C62 H124 O6 N1	22.7795061	978.9423155	POS
TG(27:0/16:0/18:1)	TG	M+NH4	C64 H126 O6 N1	22.737215	1004.957965	POS
TG(27:0/18:1/20:2)	TG	M+NH4	C68 H130 O6 N1	21.79727646	1056.989265	POS
TG(27:0/18:3/18:4)	TG	M+H	C66 H115 O6	20.25989198	1003.868816	POS
TG(27:0/6:0/18:3)	TG	M+NH4	C54 H102 O6 N1	19.2995575	860.7701655	POS
TG(27:1/10:2/18:2)	TG	M+H	C58 H103 O6	19.045	895.7749165	POS
TG(27:1/16:0/18:3)	TG	M+Na	C64 H116 O6 Na1	18.8749769	1003.866411	POS
TG(27:1/18:1/18:1)	TG	M+NH4	C66 H126 O6 N1	22.51603749	1028.957965	POS
TG(27:1/18:1/18:2)	TG	M+NH4	C66 H124 O6 N1	22.28111857	1026.942315	POS
TG(27:1/18:2/18:2)	TG	M+NH4	C66 H122 O6 N1	21.97097077	1024.926665	POS
TG(27:1/6:0/10:2)	TG	M+Na	C46 H82 O6 Na1	12.196	753.6003615	POS
TG(27:2)	TG	M+NH4	C30 H56 O6 N1	10.53289157	526.4102155	POS
TG(27:3)	TG	M+H	C30 H51 O6	7.2	507.3680165	POS
TG(27:4)	TG	M+H	C30 H49 O6	6.387	505.3523665	POS
TG(28:0/10:0/11:2)	TG	M+NH4	C52 H100 O6 N1	19.3973698	834.7545155	POS
TG(28:0/10:1/18:1)	TG	M+NH4	C59 H114 O6 N1	20.973	932.8640655	POS
TG(28:0/16:0/16:0)	TG	M+NH4	C63 H126 O6 N1	22.89240396	992.9579655	POS
TG(28:0/16:0/18:1)	TG	M+NH4	C65 H128 O6 N1	22.8597635	1018.973615	POS
TG(28:0/18:0/18:2)	TG	M+NH4	C67 H130 O6 N1	24.21024314	1044.989265	POS
TG(28:0/18:1/18:1)	TG	M+NH4	C67 H130 O6 N1	23.134	1044.989265	POS
TG(28:0/18:1/18:2)	TG	M+NH4	C67 H128 O6 N1	22.881	1042.973615	POS

TG(28:0/18:1/22:4)	TG	M+H	C71 H129 O6	21.695	1077.978366	POS
TG(28:0/18:1/22:6)	TG	M+H	C71 H125 O6	21.489	1073.947066	POS
TG(28:0/18:1/24:2)	TG	M+NH4	C73 H140 O6 N1	23.17734076	1127.067515	POS
TG(28:0/18:2/18:2)	TG	M+NH4	C67 H126 O6 N1	22.36089156	1040.957965	POS
TG(28:0/18:2/22:4)	TG	M+H	C71 H127 O6	21.412	1075.962716	POS
TG(28:0/19:0/24:0)	TG	M+H	C74 H145 O6	23.389	1130.103566	POS
TG(28:0/6:0/6:0)	TG	M+Na	C43 H82 O6 Na1	12.97126661	717.6003615	POS
TG(28:0/9:0/10:0)	TG	M+NH4	C50 H100 O6 N1	19.85972486	810.7545155	POS
TG(28:1/10:2/18:4)	TG	M+Na	C59 H100 O6 Na1	16.963	927.7412115	POS
TG(28:1/15:0/18:4)	TG	M+H	C64 H115 O6	20.39	979.8688165	POS
TG(28:1/16:0/16:0)	TG	M+NH4	C63 H124 O6 N1	23.07713486	990.9423155	POS
TG(28:1/16:0/18:3)	TG	M+Na	C65 H118 O6 Na1	19.8095946	1017.882061	POS
TG(28:1/18:1/18:1)	TG	M+NH4	C67 H128 O6 N1	22.62115036	1042.973615	POS
TG(28:1/18:1/18:2)	TG	M+Na	C67 H122 O6 Na1	20.77586734	1045.913361	POS
TG(28:1/18:1/18:4)	TG	M+H	C67 H119 O6	20.38263054	1019.900116	POS
TG(28:1/18:1/24:2)	TG	M+NH4	C73 H138 O6 N1	22.984	1125.051865	POS
TG(28:1/18:2/18:2)	TG	M+NH4	C67 H124 O6 N1	22.09536215	1038.942315	POS
TG(28:1/18:2/18:3)	TG	M+H	C67 H119 O6	20.71941232	1019.900116	POS
TG(28:1/18:2/23:0)	TG	M+NH4	C72 H138 O6 N1	23.14645733	1113.051865	POS
TG(28:1/18:2/23:1)	TG	M+NH4	C72 H136 O6 N1	22.88367525	1111.036215	POS
TG(28:1/18:2/24:2)	TG	M+NH4	C73 H136 O6 N1	22.79024503	1123.036215	POS

TG(28:1/24:1/24:1)	TG	M+Na	C79 H148 O6 Na1	22.877	1216.116811	POS
TG(28:1/6:0/22:2)	TG	M+Na	C59 H108 O6 Na1	18.243	935.8038115	POS
TG(28:1e)	TG	M+NH4	C31 H62 O5 N1	11.841	528.4622505	POS
TG(28:2)	TG	M+Na	C31 H54 O6 Na1	6.743	545.3812615	POS
TG(28:3)	TG	M+H	C31 H53 O6	17.471	521.3836665	POS
TG(28:4)	TG	M+NH4	C31 H54 O6 N1	5.383	536.3945655	POS
TG(29:0)	TG	M+Na	C32 H60 O6 Na1	7.666	563.4282115	POS
TG(29:0/16:0/16:0)	TG	M+NH4	C64 H128 O6 N1	22.9875709	1006.973615	POS
TG(29:0/16:0/18:1)	TG	M+H	C66 H127 O6	22.399	1015.962716	POS
TG(29:0/16:0/18:2)	TG	M+NH4	C66 H128 O6 N1	22.73387042	1030.973615	POS
TG(29:0/18:1/20:3)	TG	M+NH4	C70 H132 O6 N1	21.79782547	1083.004915	POS
TG(29:0/18:1/22:4)	TG	M+NH4	C72 H134 O6 N1	21.77945161	1109.020565	POS
TG(29:0/9:0/18:1)	TG	M+NH4	C59 H116 O6 N1	21.30458519	934.8797155	POS
TG(29:1/18:0/18:1)	TG	M+NH4	C68 H132 O6 N1	22.94834968	1059.004915	POS
TG(29:1/18:1/18:1)	TG	M+NH4	C68 H130 O6 N1	22.73386563	1056.989265	POS
TG(29:1/18:1/18:2)	TG	M+NH4	C68 H128 O6 N1	22.4834809	1054.973615	POS
TG(29:1/18:2/18:2)	TG	M+NH4	C68 H126 O6 N1	22.22812444	1052.957965	POS
TG(29:1/18:4/18:4)	TG	M+NH4	C68 H118 O6 N1	20.36063885	1044.895365	POS
TG(29:1/20:1/20:1)	TG	M+NH4	C72 H138 O6 N1	22.598	1113.051865	POS
TG(29:2)	TG	M+NH4	C32 H60 O6 N1	7.202	554.4415155	POS
TG(29:2e)	TG	M+H	C32 H59 O5	14.244	523.4357015	POS

TG(29:3)	TG	M+H	C32 H55 O6	9.34	535.3993165	POS
TG(29:5e)	TG	M+H	C32 H53 O5	10.398	517.3887515	POS
TG(30:0/16:0/16:0)	TG	M+NH4	C65 H130 O6 N1	23.083	1020.989265	POS
TG(30:0/16:0/18:1)	TG	M+NH4	C67 H132 O6 N1	23.0652784	1047.004915	POS
TG(30:0/18:1/18:1)	TG	M+Na	C69 H130 O6 Na1	23.04521531	1077.975961	POS
TG(30:0/18:1/18:2)	TG	M+NH4	C69 H132 O6 N1	24.937	1071.004915	POS
TG(30:0/18:1/20:4)	TG	M+H	C71 H129 O6	23.001	1077.978366	POS
TG(30:0/18:1/22:0)	TG	M+NH4	C73 H144 O6 N1	23.61825712	1131.098815	POS
TG(30:0/18:1/22:1)	TG	M+NH4	C73 H142 O6 N1	23.438	1129.083165	POS
TG(30:0/18:1/22:5)	TG	M+H	C73 H131 O6	21.74615909	1103.994016	POS
TG(30:0/18:1/24:0)	TG	M+NH4	C75 H148 O6 N1	23.605	1159.130115	POS
TG(30:0/18:1/24:2)	TG	M+NH4	C75 H144 O6 N1	23.37666911	1155.098815	POS
TG(30:0/18:2/22:6)	TG	M+H	C73 H127 O6	21.48234233	1099.962716	POS
TG(30:0/6:0/18:1)	TG	M+Na	C57 H108 O6 Na1	19.259	911.8038115	POS
TG(30:1/11:2/22:2)	TG	M+H	C66 H119 O6	20.774	1007.900116	POS
TG(30:1/16:0/18:1)	TG	M+NH4	C67 H130 O6 N1	22.84710306	1044.989265	POS
TG(30:1/18:1/18:1)	TG	M+NH4	C69 H132 O6 N1	24.617	1071.004915	POS
TG(30:1/18:1/18:2)	TG	M+NH4	C69 H130 O6 N1	22.55493403	1068.989265	POS
TG(30:1/18:1/23:0)	TG	M+NH4	C74 H144 O6 N1	23.50108279	1143.098815	POS
TG(30:1/18:2/18:2)	TG	M+NH4	C69 H128 O6 N1	22.34135517	1066.973615	POS
TG(30:1/18:2/24:2)	TG	M+NH4	C75 H140 O6 N1	22.99838247	1151.067515	POS

TG(30:1/22:1/22:1)	TG	M+Na	C77 H144 O6 Na1	22.65	1188.0855 11	POS
TG(30:1/6:0/18:2)	TG	M+Na	C57 H104 O6 Na1	18.297	907.77251 15	POS
TG(30:2)	TG	M+NH4	C33 H62 O6 N1	11.41	568.45716 55	POS
TG(30:3)	TG	M+H	C33 H57 O6	9.891	549.41496 65	POS
TG(30:4)	TG	M+H	C33 H55 O6	7.124	547.39931 65	POS
TG(30:4e)	TG	M+H	C33 H57 O5	14.855	533.42005 15	POS
TG(30:5)	TG	M+H	C33 H53 O6	12.499	545.38366 65	POS
TG(30:5e)	TG	M+H	C33 H55 O5	7.754	531.40440 15	POS
TG(31:0)	TG	M+NH4	C34 H68 O6 N1	9.1954666 4	586.50411 55	POS
TG(31:1e)	TG	M+NH4	C34 H68 O5 N1	11.732318 04	570.50920 05	POS
TG(31:2e)	TG	M+H	C34 H63 O5	10.630094 73	551.46700 15	POS
TG(31:3)	TG	M+Na	C34 H58 O6 Na1	11.361	585.41256 15	POS
TG(31:4)	TG	M+H	C34 H57 O6	9.963	561.41496 65	POS
TG(32:3)	TG	M+NH4	C35 H64 O6 N1	9.321	594.47281 55	POS
TG(33:1e)	TG	M+NH4	C36 H72 O5 N1	13.952473 08	598.54050 05	POS
TG(33:2e)	TG	M+H	C36 H67 O5	12.114	579.49830 15	POS
TG(33:3)	TG	M+NH4	C36 H66 O6 N1	2.172	608.48846 55	POS
TG(33:3e)	TG	M+Na	C36 H64 O5 Na1	12.92	599.46459 65	POS
TG(33:4e)	TG	M+H	C36 H63 O5	16.468	575.46700 15	POS
TG(33:5e)	TG	M+H	C36 H61 O5	3.845	573.45135 15	POS
TG(33:6)	TG	M+Na	C36 H56 O6 Na1	4.395	607.39691 15	POS

TG(33:6e)	TG	M+H	C36 H59 O5	5.444	571.4357015	POS
TG(34:0)	TG	M+NH4	C37 H74 O6 N1	12.051	628.5510655	POS
TG(34:1)	TG	M+Na	C37 H68 O6 Na1	15.836	631.4908115	POS
TG(34:1e)	TG	M+Na	C37 H70 O5 Na1	22.2374667	617.5115465	POS
TG(34:2)	TG	M+Na	C37 H66 O6 Na1	15.8	629.4751615	POS
TG(34:3)	TG	M+NH4	C37 H68 O6 N1	10.19097028	622.5041155	POS
TG(34:4e)	TG	M+H	C37 H65 O5	10.034	589.4826515	POS
TG(35:0)	TG	M+NH4	C38 H76 O6 N1	14.421	642.5667155	POS
TG(35:0e)	TG	M+H	C38 H75 O5	15.34129158	611.5609015	POS
TG(35:2)	TG	M+NH4	C38 H72 O6 N1	12.766	638.5354155	POS
TG(35:3)	TG	M+NH4	C38 H70 O6 N1	10.722247	636.5197655	POS
TG(35:3e)	TG	M+NH4	C38 H72 O5 N1	14.028	622.5405005	POS
TG(35:4)	TG	M+H	C38 H65 O6	7.697	617.4775665	POS
TG(35:5)	TG	M+H	C38 H63 O6	6.435413237	615.4619165	POS
TG(35:6)	TG	M+H	C38 H61 O6	10.152	613.4462665	POS
TG(35:7)	TG	M+Na	C38 H58 O6 Na1	2.385	633.4125615	POS
TG(36:3)	TG	M+NH4	C39 H72 O6 N1	10.50330949	650.5354155	POS
TG(36:4)	TG	M+H	C39 H67 O6	13.621	631.4932165	POS
TG(36:4e)	TG	M+H	C39 H69 O5	22.125	617.5139515	POS
TG(36:5)	TG	M+H	C39 H65 O6	4.775009651	629.4775665	POS
TG(36:5e)	TG	M+H	C39 H67 O5	16.673	615.4983015	POS

TG(36:6)	TG	M+H	C39 H63 O6	13.105	627.4619165	POS
TG(36:8)	TG	M+NH4	C39 H62 O6 N1	14.121	640.4571655	POS
TG(37:1)	TG	M+NH4	C40 H78 O6 N1	15.76384149	668.5823655	POS
TG(37:10)	TG	M+H	C40 H57 O6	2.003	633.4149665	POS
TG(37:2)	TG	M+Na	C40 H72 O6 Na1	14.69738332	671.5221115	POS
TG(37:4)	TG	M+NH4	C40 H72 O6 N1	15.12938921	662.5354155	POS
TG(37:5)	TG	M+NH4	C40 H70 O6 N1	11.26	660.5197655	POS
TG(37:9)	TG	M+NH4	C40 H62 O6 N1	8.731	652.4571655	POS
TG(38:1)	TG	M+NH4	C41 H80 O6 N1	13.71446025	682.5980155	POS
TG(38:10)	TG	M+NH4	C41 H62 O6 N1	24.71401267	664.4571655	POS
TG(38:2)	TG	M+NH4	C41 H78 O6 N1	11.609	680.5823655	POS
TG(38:3)	TG	M+NH4	C41 H76 O6 N1	11.77579962	678.5667155	POS
TG(38:9)	TG	M+NH4	C41 H64 O6 N1	11.941	666.4728155	POS
TG(39:2)	TG	M+NH4	C42 H80 O6 N1	17.84650654	694.5980155	POS
TG(39:3e)	TG	M+NH4	C42 H80 O5 N1	16.147	678.6031005	POS
TG(39:4e)	TG	M+Na	C42 H74 O5 Na1	13.591	681.5428465	POS
TG(39:7e)	TG	M+Na	C42 H68 O5 Na1	14.161	675.4958965	POS
TG(40:0)	TG	M+NH4	C43 H86 O6 N1	17.406	712.6449655	POS
TG(40:1e)	TG	M+NH4	C43 H86 O5 N1	18.48465235	696.6500505	POS
TG(40:2)	TG	M+NH4	C43 H82 O6 N1	15.34861566	708.6136655	POS
TG(40:4e)	TG	M+NH4	C43 H80 O5 N1	17.133	690.6031005	POS

TG(40:5e)	TG	M+Na	C43 H74 O5 Na1	10.459	693.5428465	POS
TG(41:1)	TG	M+Na	C44 H82 O6 Na1	16.627	729.6003615	POS
TG(41:2)	TG	M+NH4	C44 H84 O6 N1	16.6794243	722.6293155	POS
TG(41:3)	TG	M+NH4	C44 H82 O6 N1	15.15	720.6136655	POS
TG(41:4)	TG	M+Na	C44 H76 O6 Na1	12.442	723.5534115	POS
TG(41:5e)	TG	M+NH4	C44 H80 O5 N1	17.075	702.6031005	POS
TG(41:6)	TG	M+H	C44 H73 O6	11.076	697.5401665	POS
TG(41:6e)	TG	M+Na	C44 H74 O5 Na1	11.793	705.5428465	POS
TG(41:7e)	TG	M+H	C44 H73 O5	13.534	681.5452515	POS
TG(41:8e)	TG	M+Na	C44 H70 O5 Na1	14.24116816	701.5115465	POS
TG(42:4e)	TG	M+Na	C45 H80 O5 Na1	12.266	723.5897965	POS
TG(42:5)	TG	M+NH4	C45 H80 O6 N1	16.533	730.5980155	POS
TG(42:5e)	TG	M+NH4	C45 H82 O5 N1	16.88	716.6187505	POS
TG(42:6e)	TG	M+NH4	C45 H80 O5 N1	15.65202614	714.6031005	POS
TG(43:1)	TG	M+Na	C46 H86 O6 Na1	18.963	757.6316615	POS
TG(43:10)	TG	M+NH4	C46 H72 O6 N1	10.702	734.5354155	POS
TG(43:11)	TG	M+NH4	C46 H70 O6 N1	10.876	732.5197655	POS
TG(43:12)	TG	M+H	C46 H65 O6	9.244	713.4775665	POS
TG(43:4)	TG	M+NH4	C46 H84 O6 N1	16.486	746.6293155	POS
TG(43:4e)	TG	M+H	C46 H83 O5	15.895	715.6235015	POS
TG(43:5)	TG	M+NH4	C46 H82 O6 N1	16.663	744.6136655	POS

TG(43:5e)	TG	M+Na	C46 H80 O5 Na1	13.908470 71	735.58979 65	POS
TG(44:3)	TG	M+NH4	C47 H88 O6 N1	6.115	762.66061 55	POS
TG(44:5)	TG	M+NH4	C47 H84 O6 N1	17.076	758.62931 55	POS
TG(44:5e)	TG	M+NH4	C47 H86 O5 N1	16.502	744.65005 05	POS
TG(44:6)	TG	M+NH4	C47 H82 O6 N1	16.683076 25	756.61366 55	POS
TG(44:7)	TG	M+NH4	C47 H80 O6 N1	15.452	754.59801 55	POS
TG(44:7e)	TG	M+H	C47 H79 O5	11.264	723.59220 15	POS
TG(44:9e)	TG	M+H	C47 H75 O5	10.138	719.56090 15	POS
TG(45:0)	TG	M+NH4	C48 H96 O6 N1	20.074	782.72321 55	POS
TG(45:1)	TG	M+Na	C48 H90 O6 Na1	18.229	785.66296 15	POS
TG(45:6)	TG	M+NH4	C48 H84 O6 N1	13.921572 08	770.62931 55	POS
TG(45:6e)	TG	M+NH4	C48 H86 O5 N1	15.321	756.65005 05	POS
TG(45:7e)	TG	M+H	C48 H81 O5	15.383	737.60785 15	POS
TG(45:8e)	TG	M+NH4	C48 H82 O5 N1	13.212006 48	752.61875 05	POS
TG(46:6)	TG	M+NH4	C49 H86 O6 N1	17.259774 35	784.64496 55	POS
TG(46:7e)	TG	M+NH4	C49 H86 O5 N1	16.721	768.65005 05	POS
TG(47:2e)	TG	M+NH4	C50 H98 O5 N1	18.955	792.74395 05	POS
TG(47:6e)	TG	M+H	C50 H87 O5	17.323	767.65480 15	POS
TG(47:7)	TG	M+NH4	C50 H86 O6 N1	18.228	796.64496 55	POS
TG(48:4e)	TG	M+NH4	C51 H96 O5 N1	19.811	802.72830 05	POS
TG(48:6)	TG	M+H	C51 H87 O6	14.361	795.64971 65	POS

TG(48:8)	TG	M+H	C51 H83 O6	13.712631	791.6184165	POS
TG(49:0e)	TG	M+NH4	C52 H106 O5 N1	22.105	824.8065505	POS
TG(49:10e)	TG	M+H	C52 H83 O5	11.646	787.6235015	POS
TG(49:2e)	TG	M+NH4	C52 H102 O5 N1	19.53902386	820.7752505	POS
TG(49:4e)	TG	M+NH4	C52 H98 O5 N1	18.617	816.7439505	POS
TG(4:0/10:3/11:4)	TG	M+H	C28 H39 O6	1.19	471.2741165	POS
TG(4:0/10:3/16:0)	TG	M+H	C33 H57 O6	8.599201805	549.4149665	POS
TG(4:0/10:3/18:1)	TG	M+H	C35 H59 O6	8.754	575.4306165	POS
TG(4:0/11:1/12:4)	TG	M+H	C30 H47 O6	6.884	503.3367165	POS
TG(4:0/11:1/14:0)	TG	M+Na	C32 H58 O6 Na1	9.166	561.4125615	POS
TG(4:0/11:1/16:0)	TG	M+H	C34 H63 O6	11.36286249	567.4619165	POS
TG(4:0/11:1/18:2)	TG	M+H	C36 H63 O6	10.31084034	591.4619165	POS
TG(4:0/11:2/12:2)	TG	M+H	C30 H49 O6	6.870785456	505.3523665	POS
TG(4:0/11:2/12:4)	TG	M+H	C30 H45 O6	1.659	501.3210665	POS
TG(4:0/11:2/15:0)	TG	M+H	C33 H59 O6	8.993169687	551.4306165	POS
TG(4:0/11:3/14:1)	TG	M+H	C32 H53 O6	5.157698839	533.3836665	POS
TG(4:0/11:3/16:0)	TG	M+H	C34 H59 O6	9.003419697	563.4306165	POS
TG(4:0/11:3/18:1)	TG	M+H	C36 H61 O6	8.652	589.4462665	POS
TG(4:0/14:0/14:0)	TG	M+H	C35 H67 O6	12.21422989	583.4932165	POS
TG(4:0/14:0/18:1)	TG	M+Na	C39 H72 O6 Na1	12.57457472	659.5221115	POS
TG(4:0/14:1/18:1)	TG	M+Na	C39 H70 O6 Na1	11.56588549	657.5064615	POS

TG(4:0/14:1/18:2)	TG	M+Na	C39 H68 O6 Na1	10.50330949	655.4908115	POS
TG(4:0/14:2/18:2)	TG	M+Na	C39 H66 O6 Na1	9.76034573	653.4751615	POS
TG(4:0/6:0/10:2)	TG	M+NH4	C23 H42 O6 N1	1.292	428.3006655	POS
TG(4:0/6:0/11:3)	TG	M+NH4	C24 H42 O6 N1	1.38195481	440.3006655	POS
TG(4:0/6:0/14:2)	TG	M+H	C27 H47 O6	6.118034373	467.3367165	POS
TG(4:0/6:0/16:0)	TG	M+NH4	C29 H58 O6 N1	8.062731243	516.4258655	POS
TG(4:0/6:0/18:0)	TG	M+NH4	C31 H62 O6 N1	9.585	544.4571655	POS
TG(4:0/6:0/18:1)	TG	M+NH4	C31 H60 O6 N1	8.080306002	542.4415155	POS
TG(4:0/6:0/18:2)	TG	M+NH4	C31 H58 O6 N1	8.599360487	540.4258655	POS
TG(4:0/6:0/6:0)	TG	M+Na	C19 H34 O6 Na1	2.073416129	381.2247615	POS
TG(4:0/8:0/14:1)	TG	M+NH4	C29 H56 O6 N1	10.42631058	514.4102155	POS
TG(50:1)	TG	M+NH4	C53 H104 O6 N1	24.036	850.7858155	POS
TG(50:11)	TG	M+NH4	C53 H84 O6 N1	9.239	830.6293155	POS
TG(50:12)	TG	M+NH4	C53 H82 O6 N1	14.818	828.6136655	POS
TG(50:13)	TG	M+NH4	C53 H80 O6 N1	9.107	826.5980155	POS
TG(50:3)	TG	M+NH4	C53 H100 O6 N1	26.322	846.7545155	POS
TG(50:5)	TG	M+Na	C53 H92 O6 Na1	16.439	847.6786115	POS
TG(51:10e)	TG	M+NH4	C54 H90 O5 N1	15.578	832.6813505	POS
TG(51:11)	TG	M+NH4	C54 H86 O6 N1	15.148	844.6449655	POS
TG(51:4)	TG	M+H	C54 H97 O6	18.72810956	841.7279665	POS
TG(51:4e)	TG	M+NH4	C54 H102 O5 N1	19.07996558	844.7752505	POS

TG(51:5)	TG	M+NH4	C54 H98 O6 N1	18.372	856.7388655	POS
TG(51:5e)	TG	M+NH4	C54 H100 O5 N1	18.487	842.7596005	POS
TG(51:6)	TG	M+NH4	C54 H96 O6 N1	5.709	854.7232155	POS
TG(51:7)	TG	M+H	C54 H91 O6	16.55681898	835.6810165	POS
TG(51:7e)	TG	M+Na	C54 H92 O5 Na1	17.89	843.6836965	POS
TG(51:9e)	TG	M+H	C54 H89 O5	16.564	817.6704515	POS
TG(52:0)	TG	M+Na	C55 H106 O6 Na1	21.163	885.7881615	POS
TG(52:11)	TG	M+NH4	C55 H88 O6 N1	12.238	858.6606155	POS
TG(52:12)	TG	M+NH4	C55 H86 O6 N1	15.935	856.6449655	POS
TG(52:13)	TG	M+NH4	C55 H84 O6 N1	11.273	854.6293155	POS
TG(52:14)	TG	M+NH4	C55 H82 O6 N1	8.284	852.6136655	POS
TG(52:2)	TG	M+Na	C55 H102 O6 Na1	23.89285357	881.7568615	POS
TG(52:3)	TG	M+Na	C55 H100 O6 Na1	17.805	879.7412115	POS
TG(52:6)	TG	M+H	C55 H95 O6	16.374	851.7123165	POS
TG(52:7e)	TG	M+Na	C55 H94 O5 Na1	18.582	857.6993465	POS
TG(53:6)	TG	M+H	C56 H97 O6	17.93194073	865.7279665	POS
TG(53:8)	TG	M+NH4	C56 H96 O6 N1	16.021	878.7232155	POS
TG(54:12)	TG	M+Na	C57 H86 O6 Na1	9.788	889.6316615	POS
TG(54:13)	TG	M+NH4	C57 H88 O6 N1	11.144	882.6606155	POS
TG(54:14)	TG	M+NH4	C57 H86 O6 N1	10.792	880.6449655	POS
TG(54:2)	TG	M+Na	C57 H106 O6 Na1	18.794	909.7881615	POS

TG(54:3)	TG	M+NH4	C57 H108 O6 N1	9.767	902.81711<br>55	POS
TG(54:6)	TG	M+Na	C57 H98 O6 Na1	14.903234<br>66	901.72556<br>15	POS
TG(55:10)	TG	M+NH4	C58 H96 O6 N1	20.097	902.72321<br>55	POS
TG(55:11)	TG	M+NH4	C58 H94 O6 N1	19.659	900.70756<br>55	POS
TG(55:14)	TG	M+NH4	C58 H88 O6 N1	12.094	894.66061<br>55	POS
TG(55:15)	TG	M+NH4	C58 H86 O6 N1	9.35	892.64496<br>55	POS
TG(55:9)	TG	M+H	C58 H95 O6	22.745837<br>57	887.71231<br>65	POS
TG(56:14)	TG	M+NH4	C59 H90 O6 N1	11.115	908.67626<br>55	POS
TG(56:2)	TG	M+NH4	C59 H114 O6 N1	26.045	932.86406<br>55	POS
TG(56:3)	TG	M+NH4	C59 H112 O6 N1	6.9608978<br>05	930.84841<br>55	POS
TG(56:4)	TG	M+Na	C59 H106 O6 Na1	18.185616<br>33	933.78816<br>15	POS
TG(56:5)	TG	M+NH4	C59 H108 O6 N1	23.408	926.81711<br>55	POS
TG(56:6)	TG	M+Na	C59 H102 O6 Na1	15.869	929.75686<br>15	POS
TG(57:1)	TG	M+NH4	C60 H118 O6 N1	21.424	948.89536<br>55	POS
TG(58:1)	TG	M+NH4	C61 H120 O6 N1	23.782	962.91101<br>55	POS
TG(58:11e)	TG	M+H	C61 H99 O5	18.127	911.74870<br>15	POS
TG(58:13e)	TG	M+NH4	C61 H98 O5 N1	16.335	924.74395<br>05	POS
TG(58:3)	TG	M+NH4	C61 H116 O6 N1	26.318	958.87971<br>55	POS
TG(58:5)	TG	M+H	C61 H109 O6	20.600303<br>83	937.82186<br>65	POS
TG(58:5e)	TG	M+H	C61 H111 O5	20.803	923.84260<br>15	POS
TG(58:7)	TG	M+Na	C61 H104 O6 Na1	14.536	955.77251<br>15	POS

TG(58:7e)	TG	M+H	C61 H107 O5	19.443	919.8113015	POS
TG(58:8)	TG	M+H	C61 H103 O6	15.438	931.7749165	POS
TG(58:9e)	TG	M+H	C61 H103 O5	20.86677739	915.7800015	POS
TG(59:10)	TG	M+NH4	C62 H104 O6 N1	18.245	958.7858155	POS
TG(59:8)	TG	M+H	C62 H105 O6	18.575	945.7905665	POS
TG(60:10e)	TG	M+NH4	C63 H108 O5 N1	18.912	958.8222005	POS
TG(60:3)	TG	M+NH4	C63 H120 O6 N1	8.554	986.9110155	POS
TG(60:5)	TG	M+NH4	C63 H116 O6 N1	21.034	982.8797155	POS
TG(60:6)	TG	M+H	C63 H111 O6	24.943	963.8375165	POS
TG(60:6e)	TG	M+Na	C63 H112 O5 Na1	19.646	971.8401965	POS
TG(60:7e)	TG	M+H	C63 H111 O5	20.44732606	947.8426015	POS
TG(60:8)	TG	M+NH4	C63 H110 O6 N1	17.972	976.8327655	POS
TG(60:8e)	TG	M+H	C63 H109 O5	19.653	945.8269515	POS
TG(60:9)	TG	M+NH4	C63 H108 O6 N1	17.28	974.8171155	POS
TG(60:9e)	TG	M+H	C63 H107 O5	19.026	943.8113015	POS
TG(61:12)	TG	M+NH4	C64 H104 O6 N1	12.977	982.7858155	POS
TG(61:13)	TG	M+NH4	C64 H102 O6 N1	13.10389134	980.7701655	POS
TG(61:7)	TG	M+NH4	C64 H114 O6 N1	22.163	992.8640655	POS
TG(62:13)	TG	M+NH4	C65 H104 O6 N1	15.484	994.7858155	POS
TG(62:3)	TG	M+NH4	C65 H124 O6 N1	24.633	1014.942315	POS
TG(62:3e)	TG	M+Na	C65 H122 O5 Na1	21.383	1005.918446	POS

TG(62:4)	TG	M+H	C65 H119 O6	21.48	995.9001165	POS
TG(62:4e)	TG	M+Na	C65 H120 O5 Na1	20.784	1003.902796	POS
TG(62:6)	TG	M+NH4	C65 H118 O6 N1	21.04736946	1008.895365	POS
TG(62:6e)	TG	M+NH4	C65 H120 O5 N1	21.73624345	994.9161005	POS
TG(62:7)	TG	M+NH4	C65 H116 O6 N1	20.769	1006.879715	POS
TG(62:7e)	TG	M+NH4	C65 H118 O5 N1	21.50021921	992.9004505	POS
TG(62:8)	TG	M+NH4	C65 H114 O6 N1	19.32260338	1004.864065	POS
TG(62:8e)	TG	M+NH4	C65 H116 O5 N1	21.38604621	990.8848005	POS
TG(62:9)	TG	M+Na	C65 H108 O6 Na1	15.46	1007.803811	POS
TG(62:9e)	TG	M+H	C65 H111 O5	20.34	971.8426015	POS
TG(63:9)	TG	M+NH4	C66 H114 O6 N1	18.936	1016.864065	POS
TG(64:10)	TG	M+NH4	C67 H114 O6 N1	18.674	1028.864065	POS
TG(64:7e)	TG	M+H	C67 H119 O5	21.447	1003.905201	POS
TG(64:8)	TG	M+H	C67 H115 O6	18.436	1015.868816	POS
TG(64:8e)	TG	M+H	C67 H117 O5	21.01	1001.889551	POS
TG(64:9e)	TG	M+H	C67 H115 O5	20.36252104	999.8739015	POS
TG(65:7)	TG	M+NH4	C68 H122 O6 N1	20.268	1048.926665	POS
TG(66:0)	TG	M+NH4	C69 H138 O6 N1	23.449	1077.051865	POS
TG(66:11e)	TG	M+H	C69 H115 O5	23.62826354	1023.873901	POS
TG(66:5)	TG	M+H	C69 H125 O6	21.355	1049.947066	POS
TG(66:7)	TG	M+NH4	C69 H124 O6 N1	20.7740174	1062.942315	POS

TG(66:8)	TG	M+H	C69 H119 O6	20.779	1043.9001 16	POS
TG(66:9)	TG	M+NH4	C69 H120 O6 N1	20.091	1058.9110 15	POS
TG(67:10)	TG	M+NH4	C70 H120 O6 N1	20.386	1070.9110 15	POS
TG(67:12)	TG	M+NH4	C70 H116 O6 N1	17.712	1066.8797 15	POS
TG(67:3)	TG	M+NH4	C70 H134 O6 N1	22.049875 03	1085.0205 65	POS
TG(67:4)	TG	M+NH4	C70 H132 O6 N1	22.679027 32	1083.0049 15	POS
TG(67:7)	TG	M+Na	C70 H122 O6 Na1	18.948	1081.9133 61	POS
TG(67:8)	TG	M+Na	C70 H120 O6 Na1	18.31	1079.8977 11	POS
TG(68:10)	TG	M+NH4	C71 H122 O6 N1	20.197	1084.9266 65	POS
TG(68:3)	TG	M+Na	C71 H132 O6 Na1	22.014	1103.9916 11	POS
TG(68:4)	TG	M+NH4	C71 H134 O6 N1	22.857	1097.0205 65	POS
TG(68:8)	TG	M+NH4	C71 H126 O6 N1	20.696	1088.9579 65	POS
TG(69:1)	TG	M+NH4	C72 H142 O6 N1	23.083	1117.0831 65	POS
TG(69:2)	TG	M+NH4	C72 H140 O6 N1	23.329671 54	1115.0675 15	POS
TG(69:4)	TG	M+NH4	C72 H136 O6 N1	22.018469 63	1111.0362 15	POS
TG(6:0/10:0/10:1)	TG	M+Na	C29 H52 O6 Na1	9.8690918 87	519.36561 15	POS
TG(6:0/10:0/10:2)	TG	M+H	C29 H51 O6	3.354	495.36801 65	POS
TG(6:0/10:0/11:2)	TG	M+H	C30 H53 O6	9.6191458 58	509.38366 65	POS
TG(6:0/10:0/12:2)	TG	M+H	C31 H55 O6	7.0273229 54	523.39931 65	POS
TG(6:0/10:0/18:1)	TG	M+Na	C37 H68 O6 Na1	11.491178 44	631.49081 15	POS
TG(6:0/10:1/10:1)	TG	M+NH4	C29 H54 O6 N1	8.4534303 33	512.39456 55	POS

TG(6:0/10:1/11:1)	TG	M+H	C30 H53 O6	12.90487893	509.3836665	POS
TG(6:0/10:1/11:2)	TG	M+H	C30 H51 O6	14.964	507.3680165	POS
TG(6:0/10:1/12:1)	TG	M+Na	C31 H54 O6 Na1	12.49184544	545.3812615	POS
TG(6:0/10:1/17:1)	TG	M+H	C36 H65 O6	7.425586846	593.4775665	POS
TG(6:0/10:2/10:2)	TG	M+H	C29 H47 O6	4.407423774	491.3367165	POS
TG(6:0/10:2/11:1)	TG	M+H	C30 H51 O6	12.89245586	507.3680165	POS
TG(6:0/10:2/11:2)	TG	M+H	C30 H49 O6	5.023127926	505.3523665	POS
TG(6:0/10:2/11:3)	TG	M+H	C30 H47 O6	1.288	503.3367165	POS
TG(6:0/10:2/12:0)	TG	M+H	C31 H55 O6	8.0837446	523.3993165	POS
TG(6:0/10:2/14:3)	TG	M+H	C33 H53 O6	14.13120715	545.3836665	POS
TG(6:0/10:2/17:1)	TG	M+NH4	C36 H66 O6 N1	8.530844126	608.4884655	POS
TG(6:0/10:2/18:1)	TG	M+H	C37 H65 O6	16.053	605.4775665	POS
TG(6:0/10:2/20:3)	TG	M+H	C39 H65 O6	16.273	629.4775665	POS
TG(6:0/10:2/20:4)	TG	M+H	C39 H63 O6	4.779	627.4619165	POS
TG(6:0/10:2/21:0)	TG	M+NH4	C40 H76 O6 N1	14.098	666.5667155	POS
TG(6:0/10:3/10:3)	TG	M+H	C29 H43 O6	4.117	487.3054165	POS
TG(6:0/10:3/10:4)	TG	M+H	C29 H41 O6	3.597	485.2897665	POS
TG(6:0/10:3/11:1)	TG	M+H	C30 H49 O6	15.006	505.3523665	POS
TG(6:0/10:3/11:2)	TG	M+H	C30 H47 O6	7.687	503.3367165	POS
TG(6:0/10:3/12:0)	TG	M+H	C31 H53 O6	6.5986285	521.3836665	POS
TG(6:0/10:3/14:1)	TG	M+H	C33 H55 O6	8.833	547.3993165	POS

TG(6:0/10:3/17:1)	TG	M+NH4	C36 H64 O6 N1	5.871408704	606.4728155	POS
TG(6:0/11:1/11:2)	TG	M+H	C31 H53 O6	7.216199445	521.3836665	POS
TG(6:0/11:1/11:3)	TG	M+H	C31 H51 O6	5.667485595	519.3680165	POS
TG(6:0/11:1/18:1)	TG	M+NH4	C38 H72 O6 N1	12.08977278	638.5354155	POS
TG(6:0/11:1/18:2)	TG	M+H	C38 H67 O6	9.279067239	619.4932165	POS
TG(6:0/11:2/11:2)	TG	M+H	C31 H51 O6	11.63	519.3680165	POS
TG(6:0/11:2/12:4)	TG	M+H	C32 H49 O6	4.68	529.3523665	POS
TG(6:0/11:2/14:2)	TG	M+H	C34 H57 O6	8.442244913	561.4149665	POS
TG(6:0/11:2/18:2)	TG	M+H	C38 H65 O6	9.582	617.4775665	POS
TG(6:0/11:2/18:3)	TG	M+H	C38 H63 O6	7.724767134	615.4619165	POS
TG(6:0/11:3/18:1)	TG	M+H	C38 H65 O6	9.285829602	617.4775665	POS
TG(6:0/11:3/18:3)	TG	M+H	C38 H61 O6	9.167091445	613.4462665	POS
TG(6:0/11:4/14:0)	TG	M+H	C34 H57 O6	9.214273528	561.4149665	POS
TG(6:0/11:4/18:3)	TG	M+H	C38 H59 O6	7.739	611.4306165	POS
TG(6:0/12:0/12:3)	TG	M+NH4	C33 H60 O6 N1	10.37387923	566.4415155	POS
TG(6:0/12:0/18:1)	TG	M+H	C39 H73 O6	11.74	637.5401665	POS
TG(6:0/12:0/18:2)	TG	M+H	C39 H71 O6	9.672794478	635.5245165	POS
TG(6:0/12:0/24:0)	TG	M+NH4	C45 H90 O6 N1	18.26705054	740.6762655	POS
TG(6:0/12:1/12:2)	TG	M+H	C33 H57 O6	11.47825196	549.4149665	POS
TG(6:0/12:1/12:3)	TG	M+H	C33 H55 O6	14.76138186	547.3993165	POS
TG(6:0/12:1/14:0)	TG	M+NH4	C35 H68 O6 N1	11.16453555	598.5041155	POS

TG(6:0/12:1/18:1)	TG	M+NH4	C39 H74 O6 N1	12.90633896	652.5510655	POS
TG(6:0/12:1/18:2)	TG	M+H	C39 H69 O6	16.4926719	633.5088665	POS
TG(6:0/12:1/22:0)	TG	M+NH4	C43 H84 O6 N1	16.59850236	710.6293155	POS
TG(6:0/12:2/12:2)	TG	M+H	C33 H55 O6	16.401	547.3993165	POS
TG(6:0/12:2/12:3)	TG	M+H	C33 H53 O6	6.66100018	545.3836665	POS
TG(6:0/12:2/14:0)	TG	M+H	C35 H63 O6	8.433	579.4619165	POS
TG(6:0/12:2/18:1)	TG	M+H	C39 H69 O6	11.57557325	633.5088665	POS
TG(6:0/12:2/18:2)	TG	M+Na	C39 H66 O6 Na1	10.45978159	653.4751615	POS
TG(6:0/12:2/18:3)	TG	M+H	C39 H65 O6	15.19923386	629.4775665	POS
TG(6:0/12:2/24:0)	TG	M+NH4	C45 H86 O6 N1	16.55280531	736.6449655	POS
TG(6:0/12:3/18:1)	TG	M+H	C39 H67 O6	9.41	631.4932165	POS
TG(6:0/12:3/18:2)	TG	M+H	C39 H65 O6	6.778464679	629.4775665	POS
TG(6:0/12:3/18:3)	TG	M+H	C39 H63 O6	5.887266901	627.4619165	POS
TG(6:0/12:4/23:1)	TG	M+Na	C44 H74 O6 Na1	12.041	721.5377615	POS
TG(6:0/13:0/18:1)	TG	M+NH4	C40 H78 O6 N1	16.33297402	668.5823655	POS
TG(6:0/13:0/18:2)	TG	M+NH4	C40 H76 O6 N1	13.62015838	666.5667155	POS
TG(6:0/13:0/18:3)	TG	M+NH4	C40 H74 O6 N1	12.65699503	664.5510655	POS
TG(6:0/14:0/14:4)	TG	M+H	C37 H63 O6	11.164	603.4619165	POS
TG(6:0/14:0/18:1)	TG	M+NH4	C41 H80 O6 N1	12.557	682.5980155	POS
TG(6:0/14:0/18:3)	TG	M+NH4	C41 H76 O6 N1	10.47738919	678.5667155	POS
TG(6:0/14:1/18:1)	TG	M+NH4	C41 H78 O6 N1	17.44192159	680.5823655	POS

TG(6:0/14:2/14:2)	TG	M+H	C37 H63 O6	9.688851456	603.4619165	POS
TG(6:0/14:2/14:3)	TG	M+H	C37 H61 O6	8.537	601.4462665	POS
TG(6:0/14:3/14:3)	TG	M+Na	C37 H58 O6 Na1	5.257272798	621.4125615	POS
TG(6:0/14:3/18:1)	TG	M+H	C41 H71 O6	12.60188465	659.5245165	POS
TG(6:0/14:3/18:2)	TG	M+H	C41 H69 O6	11.0541219	657.5088665	POS
TG(6:0/14:4/18:1)	TG	M+H	C41 H69 O6	11.473	657.5088665	POS
TG(6:0/14:4/18:2)	TG	M+H	C41 H67 O6	10.477322	655.4932165	POS
TG(6:0/14:4/18:3)	TG	M+H	C41 H65 O6	9.536	653.4775665	POS
TG(6:0/17:1/18:1)	TG	M+NH4	C44 H84 O6 N1	18.53108391	722.6293155	POS
TG(6:0/18:1/18:1)	TG	M+Na	C45 H82 O6 Na1	16.02	741.6003615	POS
TG(6:0/18:1/18:2)	TG	M+H	C45 H81 O6	16.51195711	717.6027665	POS
TG(6:0/18:1/20:4)	TG	M+H	C47 H81 O6	16.631	741.6027665	POS
TG(6:0/18:1/23:0)	TG	M+NH4	C50 H98 O6 N1	20.323	808.7388655	POS
TG(6:0/18:2/18:2)	TG	M+NH4	C45 H82 O6 N1	17.39785205	732.6136655	POS
TG(6:0/18:2/23:0)	TG	M+NH4	C50 H96 O6 N1	18.35036423	806.7232155	POS
TG(6:0/18:3/22:3)	TG	M+H	C49 H83 O6	14.82909282	767.6184165	POS
TG(6:0/6:0/10:0)	TG	M+Na	C25 H46 O6 Na1	4.487	465.3186615	POS
TG(6:0/6:0/10:2)	TG	M+H	C25 H43 O6	2.986120866	439.3054165	POS
TG(6:0/6:0/10:3)	TG	M+H	C25 H41 O6	4.475	437.2897665	POS
TG(6:0/6:0/10:4)	TG	M+H	C25 H39 O6	7.806	435.2741165	POS
TG(6:0/6:0/11:1)	TG	M+Na	C26 H46 O6 Na1	3.646	477.3186615	POS

TG(6:0/6:0/11:2)	TG	M+Na	C26 H44 O6 Na1	1.970090966	475.3030115	POS
TG(6:0/6:0/11:3)	TG	M+H	C26 H43 O6	2.971	451.3054165	POS
TG(6:0/6:0/11:4)	TG	M+H	C26 H41 O6	2.295	449.2897665	POS
TG(6:0/6:0/12:0)	TG	M+NH4	C27 H54 O6 N1	5.169422307	488.3945655	POS
TG(6:0/6:0/12:1)	TG	M+Na	C27 H48 O6 Na1	5.947	491.3343115	POS
TG(6:0/6:0/12:2)	TG	M+NH4	C27 H50 O6 N1	2.996091521	484.3632655	POS
TG(6:0/6:0/12:3)	TG	M+H	C27 H45 O6	3.203995307	465.3210665	POS
TG(6:0/6:0/12:4)	TG	M+H	C27 H43 O6	2.071207679	463.3054165	POS
TG(6:0/6:0/14:0)	TG	M+Na	C29 H54 O6 Na1	6.564014365	521.3812615	POS
TG(6:0/6:0/14:1)	TG	M+Na	C29 H52 O6 Na1	12.422	519.3656115	POS
TG(6:0/6:0/14:2)	TG	M+H	C29 H51 O6	5.969	495.3680165	POS
TG(6:0/6:0/14:4)	TG	M+H	C29 H47 O6	3.788	491.3367165	POS
TG(6:0/6:0/18:2)	TG	M+NH4	C33 H62 O6 N1	9.489	568.4571655	POS
TG(6:0/6:0/21:1)	TG	M+NH4	C36 H70 O6 N1	10.31199473	612.5197655	POS
TG(6:0/6:0/22:1)	TG	M+Na	C37 H68 O6 Na1	7.78863667	631.4908115	POS
TG(6:0/6:0/22:2)	TG	M+Na	C37 H66 O6 Na1	6.757	629.4751615	POS
TG(6:0/6:0/23:0)	TG	M+Na	C38 H72 O6 Na1	15.65463142	647.5221115	POS
TG(6:0/6:0/6:0)	TG	M+NH4	C21 H42 O6 N1	1.793450352	404.3006655	POS
TG(6:0/8:0/10:2)	TG	M+NH4	C27 H50 O6 N1	4.818908777	484.3632655	POS
TG(6:0/8:0/10:4)	TG	M+H	C27 H43 O6	6.492873818	463.3054165	POS
TG(6:0/8:0/11:3)	TG	M+H	C28 H47 O6	5.797	479.3367165	POS

TG(6:0/8:0/12:1)	TG	M+NH4	C29 H56 O6 N1	9.568	514.4102155	POS
TG(6:0/8:0/8:0)	TG	M+NH4	C25 H50 O6 N1	3.995220938	460.3632655	POS
TG(6:0/8:0/9:0)	TG	M+NH4	C26 H52 O6 N1	4.521316719	474.3789155	POS
TG(6:0/9:0/10:3)	TG	M+NH4	C28 H50 O6 N1	1.628	496.3632655	POS
TG(6:0/9:0/11:3)	TG	M+H	C29 H49 O6	2.62	493.3523665	POS
TG(6:0/9:0/12:2)	TG	M+H	C30 H53 O6	8.661547165	509.3836665	POS
TG(6:0/9:0/18:2)	TG	M+NH4	C36 H68 O6 N1	11.08537211	610.5041155	POS
TG(6:0/9:0/18:3)	TG	M+NH4	C36 H66 O6 N1	7.171352752	608.4884655	POS
TG(6:0/9:0/21:1)	TG	M+H	C39 H73 O6	12.13182023	637.5401665	POS
TG(6:0/9:0/9:0)	TG	M+NH4	C27 H54 O6 N1	6.254	488.3945655	POS
TG(70:3)	TG	M+Na	C73 H136 O6 Na1	22.115	1132.022911	POS
TG(70:5)	TG	M+Na	C73 H132 O6 Na1	21.617	1127.991611	POS
TG(70:6)	TG	M+H	C73 H131 O6	22.07840766	1103.994016	POS
TG(71:2)	TG	M+NH4	C74 H144 O6 N1	22.755	1143.098815	POS
TG(71:3)	TG	M+NH4	C74 H142 O6 N1	23.278	1141.083165	POS
TG(71:4)	TG	M+NH4	C74 H140 O6 N1	23.069	1139.067515	POS
TG(72:2)	TG	M+NH4	C75 H146 O6 N1	23.072	1157.114465	POS
TG(72:3)	TG	M+Na	C75 H140 O6 Na1	22.458	1160.054211	POS
TG(72:4)	TG	M+NH4	C75 H142 O6 N1	23.17995734	1153.083165	POS
TG(72:5)	TG	M+Na	C75 H136 O6 Na1	21.973	1156.022911	POS
TG(72:6)	TG	M+Na	C75 H134 O6 Na1	20.961	1154.007261	POS

TG(74:3)	TG	M+NH4	C77 H148 O6 N1	23.56	1183.130115	POS
TG(76:1)	TG	M+NH4	C79 H156 O6 N1	24.05	1215.192715	POS
TG(78:4)	TG	M+Na	C81 H150 O6 Na1	22.873	1242.132461	POS
TG(8:0/10:0/10:0)	TG	M+NH4	C31 H62 O6 N1	12.77924025	544.4571655	POS
TG(8:0/10:0/11:3)	TG	M+Na	C32 H54 O6 Na1	7.220924862	557.3812615	POS
TG(8:0/10:0/18:1)	TG	M+H	C39 H73 O6	13.70183464	637.5401665	POS
TG(8:0/10:0/18:2)	TG	M+H	C39 H71 O6	10.36775048	635.5245165	POS
TG(8:0/10:0/24:1)	TG	M+NH4	C45 H88 O6 N1	17.12420179	738.6606155	POS
TG(8:0/10:1/10:1)	TG	M+H	C31 H55 O6	9.978175668	523.3993165	POS
TG(8:0/10:1/11:3)	TG	M+H	C32 H53 O6	15.30039629	533.3836665	POS
TG(8:0/10:1/12:3)	TG	M+H	C33 H55 O6	7.760333472	547.3993165	POS
TG(8:0/10:1/18:1)	TG	M+H	C39 H71 O6	12.63696638	635.5245165	POS
TG(8:0/10:1/18:2)	TG	M+H	C39 H69 O6	12.49710236	633.5088665	POS
TG(8:0/10:1/18:3)	TG	M+H	C39 H67 O6	7.846399975	631.4932165	POS
TG(8:0/10:1/24:0)	TG	M+NH4	C45 H88 O6 N1	17.64513443	738.6606155	POS
TG(8:0/10:2/12:3)	TG	M+H	C33 H53 O6	14.90577787	545.3836665	POS
TG(8:0/10:2/14:0)	TG	M+NH4	C35 H66 O6 N1	9.996883818	596.4884655	POS
TG(8:0/10:2/18:1)	TG	M+NH4	C39 H72 O6 N1	15.45191597	650.5354155	POS
TG(8:0/10:2/18:2)	TG	M+H	C39 H67 O6	8.975895409	631.4932165	POS
TG(8:0/10:2/18:3)	TG	M+H	C39 H65 O6	7.983	629.4775665	POS
TG(8:0/10:3/12:3)	TG	M+H	C33 H51 O6	8.929482475	543.3680165	POS

TG(8:0/10:3/18:2)	TG	M+H	C39 H65 O6	9.742039992	629.4775665	POS
TG(8:0/10:3/18:3)	TG	M+H	C39 H63 O6	8.838933961	627.4619165	POS
TG(8:0/10:4/17:1)	TG	M+H	C38 H63 O6	8.384432114	615.4619165	POS
TG(8:0/11:1/18:1)	TG	M+NH4	C40 H76 O6 N1	14.69738332	666.5667155	POS
TG(8:0/11:2/11:2)	TG	M+H	C33 H55 O6	6.609930824	547.3993165	POS
TG(8:0/11:2/12:3)	TG	M+H	C34 H55 O6	11.37391062	559.3993165	POS
TG(8:0/11:3/17:1)	TG	M+H	C39 H67 O6	16.84282445	631.4932165	POS
TG(8:0/11:3/23:1)	TG	M+Na	C45 H78 O6 Na1	12.22446739	737.5690615	POS
TG(8:0/12:4/24:1)	TG	M+NH4	C47 H84 O6 N1	15.35394283	758.6293155	POS
TG(8:0/17:1/22:5)	TG	M+H	C50 H85 O6	13.383	781.6340665	POS
TG(8:0/18:1/18:1)	TG	M+NH4	C47 H90 O6 N1	19.42673663	764.6762655	POS
TG(8:0/18:1/18:2)	TG	M+NH4	C47 H88 O6 N1	18.85536123	762.6606155	POS
TG(8:0/18:2/18:2)	TG	M+NH4	C47 H86 O6 N1	18.19307833	760.6449655	POS
TG(8:0/18:3/18:3)	TG	M+NH4	C47 H82 O6 N1	15.5	756.6136655	POS
TG(8:0/8:0/10:0)	TG	M+NH4	C29 H58 O6 N1	11.33324461	516.4258655	POS
TG(8:0/8:0/10:1)	TG	M+Na	C29 H52 O6 Na1	5.992	519.3656115	POS
TG(8:0/8:0/10:3)	TG	M+H	C29 H49 O6	5.455	493.3523665	POS
TG(8:0/8:0/11:1)	TG	M+NH4	C30 H58 O6 N1	9.135957895	528.4258655	POS
TG(8:0/8:0/11:2)	TG	M+NH4	C30 H56 O6 N1	11.27227599	526.4102155	POS
TG(8:0/8:0/11:3)	TG	M+H	C30 H51 O6	7.68	507.3680165	POS
TG(8:0/8:0/11:4)	TG	M+NH4	C30 H52 O6 N1	1.937	522.3789155	POS

TG(8:0/8:0/14:4)	TG	M+H	C33 H55 O6	12.042482 73	547.39931 65	POS
TG(8:0/8:0/17:1)	TG	M+H	C36 H67 O6	8.6463997	595.49321 65	POS
TG(8:0/8:0/18:1)	TG	M+Na	C37 H68 O6 Na1	8.966	631.49081 15	POS
TG(8:0/8:0/9:0)	TG	M+NH4	C28 H56 O6 N1	9.101	502.41021 55	POS
TG(8:0/9:0/10:2)	TG	M+H	C30 H53 O6	7.7118548 85	509.38366 65	POS
TG(8:0/9:0/10:3)	TG	M+H	C30 H51 O6	7.974	507.36801 65	POS
TG(8:0/9:0/10:4)	TG	M+H	C30 H49 O6	2.385	505.35236 65	POS
TG(8:0/9:0/18:1)	TG	M+NH4	C38 H74 O6 N1	13.020666 13	640.55106 55	POS
TG(9:0/10:0/10:0)	TG	M+NH4	C32 H64 O6 N1	7.327	558.47281 55	POS
TG(9:0/10:0/14:0)	TG	M+H	C36 H69 O6	9.761	597.50886 65	POS
TG(9:0/10:1/10:1)	TG	M+NH4	C32 H60 O6 N1	8.5615094 37	554.44151 55	POS
TG(9:0/10:1/11:3)	TG	M+H	C33 H55 O6	16.677546 78	547.39931 65	POS
TG(9:0/10:1/18:1)	TG	M+NH4	C40 H76 O6 N1	15.165282 64	666.56671 55	POS
TG(9:0/10:1/18:2)	TG	M+NH4	C40 H74 O6 N1	16.207130 35	664.55106 55	POS
TG(9:0/10:2/11:3)	TG	M+H	C33 H53 O6	5.9916201 33	545.38366 65	POS
TG(9:0/10:2/12:3)	TG	M+H	C34 H55 O6	8.093	559.39931 65	POS
TG(9:0/10:2/14:0)	TG	M+H	C36 H65 O6	5.4058036 47	593.47756 65	POS
TG(9:0/10:3/11:3)	TG	M+H	C33 H51 O6	10.612	543.36801 65	POS
TG(9:0/11:3/11:3)	TG	M+H	C34 H53 O6	7.3934991 15	557.38366 65	POS
TG(9:0/11:3/14:4)	TG	M+Na	C37 H56 O6 Na1	4.5230734 41	619.39691 15	POS
TG(9:0/12:2/12:2)	TG	M+H	C36 H61 O6	9.6947300 97	589.44626 65	POS

TG(9:0/14:2/18:1)	TG	M+NH4	C44 H82 O6 N1	17.80467634	720.6136655	POS
TG(9:0/14:2/18:2)	TG	M+NH4	C44 H80 O6 N1	16.916	718.5980155	POS
TG(9:0/18:1/18:1)	TG	M+Na	C48 H88 O6 Na1	17.553	783.6473115	POS
TG(9:0/18:1/18:2)	TG	M+Na	C48 H86 O6 Na1	17.39387378	781.6316615	POS
TG(9:0/18:1/18:3)	TG	M+H	C48 H85 O6	18.94971858	757.6340665	POS
TG(9:0/18:1/22:5)	TG	M+H	C52 H89 O6	16.73851792	809.6653665	POS
TG(9:0/18:2/18:2)	TG	M+NH4	C48 H88 O6 N1	18.54611316	774.6606155	POS
TG(9:0/18:2/18:3)	TG	M+NH4	C48 H86 O6 N1	17.92314328	772.6449655	POS
TG(9:0/18:3/18:3)	TG	M+H	C48 H81 O6	14.86974708	753.6027665	POS
TG(9:0/9:0/10:0)	TG	M+Na	C31 H58 O6 Na1	8.708	549.4125615	POS
TG(9:0/9:0/10:1)	TG	M+Na	C31 H56 O6 Na1	7.808	547.3969115	POS
TG(9:0/9:0/10:3)	TG	M+Na	C31 H52 O6 Na1	11.38584834	543.3656115	POS
TG(9:0/9:0/11:2)	TG	M+Na	C32 H56 O6 Na1	11.358	559.3969115	POS
TG(9:0/9:0/11:3)	TG	M+H	C32 H55 O6	6.839379785	535.3993165	POS
TG(9:0/9:0/18:1)	TG	M+H	C39 H73 O6	15.24725209	637.5401665	POS
WE(26:0/18:3)	WE	M+H	H83 C44 O2	19.857	643.6387565	POS
WE(28:0/18:3)	WE	M+H	H87 C46 O2	22.556	671.6700565	POS
WE(29:0/18:3)	WE	M+H	H89 C47 O2	22.957	685.6857065	POS
WE(29:1/16:0)	WE	M+H	H89 C45 O2	23.229	661.6857065	POS
WE(2:0/20:2)	WE	M+NH4	H44 C22 O2 N1	4.11	354.3366555	POS
WE(30:0/18:3)	WE	M+H	H91 C48 O2	20.777	699.7013565	POS

WE(3:0/18:1)	WE	M+NH4	H44 C21 O2 N1	3.688035798	342.3366555	POS
WE(3:0/20:1)	WE	M+NH4	H48 C23 O2 N1	5.49269791	370.3679555	POS
WE(3:0/20:2)	WE	M+NH4	H46 C23 O2 N1	3.906310895	368.3523055	POS
WE(3:0/20:3)	WE	M+NH4	H44 C23 O2 N1	2.868663496	366.3366555	POS
WE(5:0/18:3)	WE	M+H	H41 C23 O2	6.388	349.3101065	POS
WE(7:0/18:3)	WE	M+H	H45 C25 O2	9.411	377.3414065	POS
WE(8:0/19:3)	WE	M+H	H49 C27 O2	10.971	405.3727065	POS
WE(9:0/18:3)	WE	M+H	H49 C27 O2	12.63	405.3727065	POS
ZyE(0:0)	ZyE	M+H	C27 H45 O1	11.5390663	385.3464915	POS
ZyE(19:1)	ZyE	M+H	C46 H79 O2	19.1013976	663.6074565	POS
ZyE(19:2)	ZyE	M+H	C46 H77 O2	21.119	661.5918065	POS
ZyE(19:3)	ZyE	M+H	C46 H75 O2	17.82762464	659.5761565	POS
ZyE(20:1)	ZyE	M+H	C47 H81 O2	19.39	677.6231065	POS
ZyE(20:2)	ZyE	M+H	C47 H79 O2	18.952	675.6074565	POS
ZyE(20:3)	ZyE	M+H	C47 H77 O2	17.89607771	673.5918065	POS
ZyE(20:4)	ZyE	M+H	C47 H75 O2	20.21035498	671.5761565	POS
ZyE(21:2)	ZyE	M+NH4	C48 H84 O2 N1	21.10167586	706.6496555	POS
ZyE(21:3)	ZyE	M+H	C48 H79 O2	18.5125485	687.6074565	POS
ZyE(21:4)	ZyE	M+H	C48 H77 O2	13.95475263	685.5918065	POS
ZyE(21:5)	ZyE	M+NH4	C48 H78 O2 N1	14.522	700.6027055	POS
ZyE(22:2)	ZyE	M+NH4	C49 H86 O2 N1	21.10058735	720.6653055	POS

ZyE(23:6)	ZyE	M+NH4	C50 H80 O2 N1	16.625	726.61835 55	POS
ZyE(24:6)	ZyE	M+NH4	C51 H82 O2 N1	16.554132 68	740.63400 55	POS
ZyE(2:0)	ZyE	M+H	C29 H47 O2	14.39	427.35705 65	POS
ZyE(33:6)	ZyE	M+H	C60 H97 O2	19.973	849.74830 65	POS
ZyE(35:6)	ZyE	M+NH4	C62 H104 O2 N1	19.453	894.80615 55	POS
ZyE(37:6)	ZyE	M+NH4	C64 H108 O2 N1	20.819	922.83745 55	POS
ZyE(4:0)	ZyE	M+H	C31 H51 O2	8.163	455.38835 65	POS
cPA(16:0)	cPA	M-H	C19 H36 O6 N0 P1	3.5586177 37	391.22550 15	NEG
cPA(18:0)	cPA	M-H	C21 H40 O6 N0 P1	5.4018944 57	419.25680 15	NEG
cPA(18:1)	cPA	M-H	C21 H38 O6 N0 P1	3.7790375 03	417.24115 15	NEG
cPA(18:2)	cPA	M-H	C21 H36 O6 N0 P1	2.7651437 02	415.22550 15	NEG
cPA(20:0)	cPA	M-H	C23 H44 O6 N0 P1	7.5309615 22	447.28810 15	NEG
cPA(24:0)	cPA	M-H	C27 H52 O6 N0 P1	10.828	503.35070 15	NEG
dMePE(16:0/18:1)	dMePE	M-H	C41 H79 O8 N1 P1	14.616315 48	744.55488 05	NEG
dMePE(16:0/18:2)	dMePE	M-H	C41 H77 O8 N1 P1	13.500140 89	742.53923 05	NEG
dMePE(16:1/18:1)	dMePE	M-H	C41 H77 O8 N1 P1	12.400651 48	742.53923 05	NEG
dMePE(18:0/18:1)	dMePE	M-H	C43 H83 O8 N1 P1	15.927	772.58618 05	NEG
dMePE(18:1/14:0)	dMePE	M-H	C39 H75 O8 N1 P1	13.198645 17	716.52358 05	NEG
dMePE(18:1/18:1)	dMePE	M-H	C43 H81 O8 N1 P1	14.676651 03	770.57053 05	NEG
dMePE(18:1/18:2)	dMePE	M-H	C43 H79 O8 N1 P1	13.555566 29	768.55488 05	NEG
dMePE(18:2/18:2)	dMePE	M-H	C43 H77 O8 N1 P1	12.409814 76	766.53923 05	NEG

dMePE(19:2)	dMePE	M-H	C26 H47 O8 N1 P1	3.255652342	532.3044805	NEG
dMePE(29:0)	dMePE	M-H	C36 H71 O8 N1 P1	7.67876322	676.4922805	NEG
dMePE(32:1)	dMePE	M-H	C39 H75 O8 N1 P1	12.459	716.5235805	NEG
dMePE(33:2)	dMePE	M-H	C40 H75 O8 N1 P1	13.838	728.5235805	NEG
dMePE(35:2)	dMePE	M-H	C42 H79 O8 N1 P1	14.97850805	756.5548805	NEG
dMePE(35:3)	dMePE	M-H	C42 H77 O8 N1 P1	13.87781621	754.5392305	NEG
phSM(d33:1)	phSM	M+HCOO	C39 H78 O9 N2 P1	14.921	749.5450445	POS
phSM(d34:2)	phSM	M+CH3COO	C41 H80 O9 N2 P1	14.892	775.5606945	POS
phSM(d36:3)	phSM	M+CH3COO	C43 H82 O9 N2 P1	14.908	801.5763445	POS
phSM(d37:3)	phSM	M+CH3COO	C44 H84 O9 N2 P1	14.243	815.5919945	POS
phSM(d38:1)	phSM	M+CH3COO	C45 H90 O9 N2 P1	16.161	833.6389445	POS
